# Steroid hormones regulate genome-wide epigenetic programming and gene transcription in human endometrial cells with marked aberrancies in endometriosis

**DOI:** 10.1371/journal.pgen.1008601

**Published:** 2020-06-17

**Authors:** Sahar Houshdaran, Ashwini B. Oke, Jennifer C. Fung, Kim Chi Vo, Camran Nezhat, Linda C. Giudice

**Affiliations:** 1 University of California San Francisco, Dept. of Obstetrics, Gynecology and Reproductive Sciences, San Francisco, California, United States of America; 2 Camran Nezhat Institute, Palo Alto, California, United States of America; University of Pennsylvania, UNITED STATES

## Abstract

Programmed cellular responses to cycling ovarian-derived steroid hormones are central to normal endometrial function. Abnormalities therein, as in the estrogen-dependent, progesterone-“resistant” disorder, endometriosis, predispose to infertility and poor pregnancy outcomes. The endometrial stromal fibroblast (eSF) is a master regulator of pregnancy success. However, the complex hormone-epigenome-transcriptome interplay in eSF by each individual steroid hormone, estradiol (E_2_) and/or progesterone (P_4_), under physiologic and pathophysiologic conditions, is poorly understood and was investigated herein. Genome-wide analysis in normal, early and late stage eutopic eSF revealed: i) In contrast to P_4_, E_2_ extensively affected the eSF DNA methylome and transcriptome. Importantly, E_2_ resulted in a more open versus closed chromatin, confirmed by histone modification analysis. Combined E_2_ with P_4_ affected a totally different landscape than E_2_ or P_4_ alone. ii) P_4_ responses were aberrant in early and late stage endometriosis, and mapping differentially methylated CpG sites with progesterone receptor targets from the literature revealed different but not decreased P_4_-targets, leading to question the P_4_-“resistant” phenotype in endometriosis. Interestingly, an aberrant E_2_-response was noted in eSF from endometriosis women; iii) Steroid hormones affected specific genomic contexts and locations, significantly enriching enhancers and intergenic regions and minimally involving proximal promoters and CpG islands, regardless of hormone type and eSF disease state. iv) In eSF from women with endometriosis, aberrant hormone-induced methylation signatures were mainly due to existing DNA methylation marks prior to hormone treatments and involved known endometriosis genes and pathways. v) Distinct DNA methylation and transcriptomic signatures revealed early and late stage endometriosis comprise unique disease subtypes. Taken together, the data herein, for the first time, provide significant insight into the hormone-epigenome-transcriptome interplay of each steroid hormone in *normal* eSF, and aberrant E_2_ response, distinct disease subtypes, and pre-existing epigenetic aberrancies in the setting of endometriosis, provide mechanistic insights into how endometriosis affects endometrial function/dysfunction.

## Author summary

Steroid hormones regulate genes in select tissues, including the lining of the uterus (“endometrium”). If the hormone response in the endometrium is abnormal, infertility and poor pregnancy outcomes can result. Interaction of steroid hormones with the second genome (“epigenome”) to regulate gene expression is poorly understood. Thus, we studied the response to estrogen (E_2_), progesterone (P_4_) and their combination of the endometrial stromal fibroblast (eSF), a cell that is pivotal in pregnancy. The cells were derived from normal woman and those with endometriosis, an E_2_-responsive, P_4_-resistant, inflammatory disorder with implantation-based infertility and poor pregnancy outcomes. Hormone-regulated genome-wide DNA methylation (a key epigenetic mechanism) and gene expression patterns and profiles were distinct for each hormone and in normal versus disease. Advanced stage disease had the most blunted response, and novel, not previously reported E_2_ responses were observed in eSF from women with endometriosis. Intergenic regions and enhancers were significantly enriched, with minimal involvement of gene proximal promoters, indicating a broad, genome-wide hormone effect. Unexpectedly, pre-existing aberrant DNA methylation signatures in eSF from women with endometriosis were found and revealed distinct disease sub-types. These data advance our understanding of genome-wide hormone-epigenome dynamics critical in hormone-responsive tissues with downstream impact on physiologic processes.

## Introduction

Endometrium is a dynamic tissue whose cellular components undergo cyclic proliferation and differentiation, preparing for embryo implantation by highly coordinated spatiotemporal actions of ovarian-derived estradiol (E_2_) and progesterone (P_4_) [1,2]. These hormones bind cognate receptors [estrogen receptor (ER) and progesterone receptor (PR)], whose activities are tightly regulated by post-translational modifications and interactions with cell- and tissue-specific co-regulators [3–5]. Binding ER and PR leads to their nuclear translocation, complexing with nuclear response elements, remodeling chromatin by co-modulator recruitment [3], and alteration of the transcriptional machinery. In endometrium, dynamic circulating E_2_ and P_4_ levels drive the normal functionality of the tissue. Moreover, environmental and inflammatory signals can alter steroid hormone-driven endometrial gene transcription and cellular function resulting in tissue dyshomeostasis [6], including endometrial hyperplasia and cancer, endometrial-based infertility, endometriosis, and poor pregnancy outcomes [7,8]. While changes in chromatin accessibility, PR targets and changes in histones and gene expression in eSF decidualization by E_2_, cAMP and MPA have been shown [9–11], how E_2_ and P_4_ individually interact with the endometrial epigenome normally or in inflammatory disorders that compromise endometrial function, e.g., as in the disorder endometriosis, are incompletely understood. We *hypothesized* that these steroid hormones induce unique genome-wide signatures in normal human endometrial stromal fibroblasts with aberrant signatures in endometrial cells from women with endometriosis, and their effect on the epigenome is directed by specific genomic sequences and locations.

Endometriosis is a common, chronic disorder wherein endometrial tissue, shed into the pelvis at menses, elicits an inflammatory response, neuroangiogenesis and fibrosis, resulting in infertility and chronic pelvic pain [12]. Hallmarks of the disorder are its dependence on E_2_ for growth, disrupted P_4_ signaling caused by chronic inflammation in endometriosis lesions and in the eutopic endometrium (uterine lining) [13], and epigenetic chromatin changes that determine endometrial cellular responses to mitogenic and differentiative signals [6,12,14]. Normally, the eutopic endometrial DNA methylome varies according to the hormonal milieu, with greatest differences in the E_2_-dominant (proliferative) versus P_4_-dominant (secretory) phase of the cycle and associated with gene expression changes [10,13–16]. Chronic inflammation affects the chromatin landscape in endometrium of women and animal models of endometriosis [6]. These observations on bulk tissue raise fundamental questions about steroid hormone-epigenome interactions in cellular components of the endometrium normally and in women with disease, how steroid hormones affect the epigenome, how the epigenome affects steroid hormone response and action, and if there are epigenetic differences in endometrium of women with endometriosis and different stages of disease, and if so, what role they play in these processes.

Herein, we studied responses of endometrial stromal fibroblasts (eSF) isolated from *normal* women (controls) and those with endometriosis. eSF comprise a major endometrial cell type whose programmed responses to E_2_ and P_4_ are essential for pregnancy success and whose responses are compromised in inflammatory disorders [17], including endometriosis [18,19]. Given the centrality of a normal eSF P_4_ response for pregnancy and that women with endometriosis have infertility and poor pregnancy outcomes reported by some to be due, in part, to altered eSF basal gene expression and abnormal response to P_4_ [18–21]_,_ understanding steroid hormone-signaling and regulation of transcription in this cell type is paramount. Moreover, controls for this study were women with no known gynecologic or systemic disorders, thereby enabling establishment of a normative platform for steroid hormone effects on the epigenome and gene transcription in this cell type in endometrium, the tissue that is the anatomic pre-requisite for continuation of the species.

## Results

### Distinct Effects of Ovarian Steroid Hormones in *Normal Human Endometrium*

We assessed the genome-wide effect of individual steroid hormones, E_2_ and P_4_, and their combination (E_2_+ P_4_) on endometrial stromal fibroblasts (eSF) after 14 days of exposure mimicking the timeframe in the menstrual cycle for maximal hormone responsiveness. Since the effects of E_2_ and P_4_ individually and together on the hormone-epigenome interplay in *normal* endometrial cells was unknown and was a main aim of this study, we applied stringent criteria and utilized only endometrial samples from extensively screened volunteers without any gynecologic disorders and no uterine pathology (NUP), with confirmed *in vitro* eSF progesterone responsiveness (see methods and **S1 Fig**).

**Fig 1:**
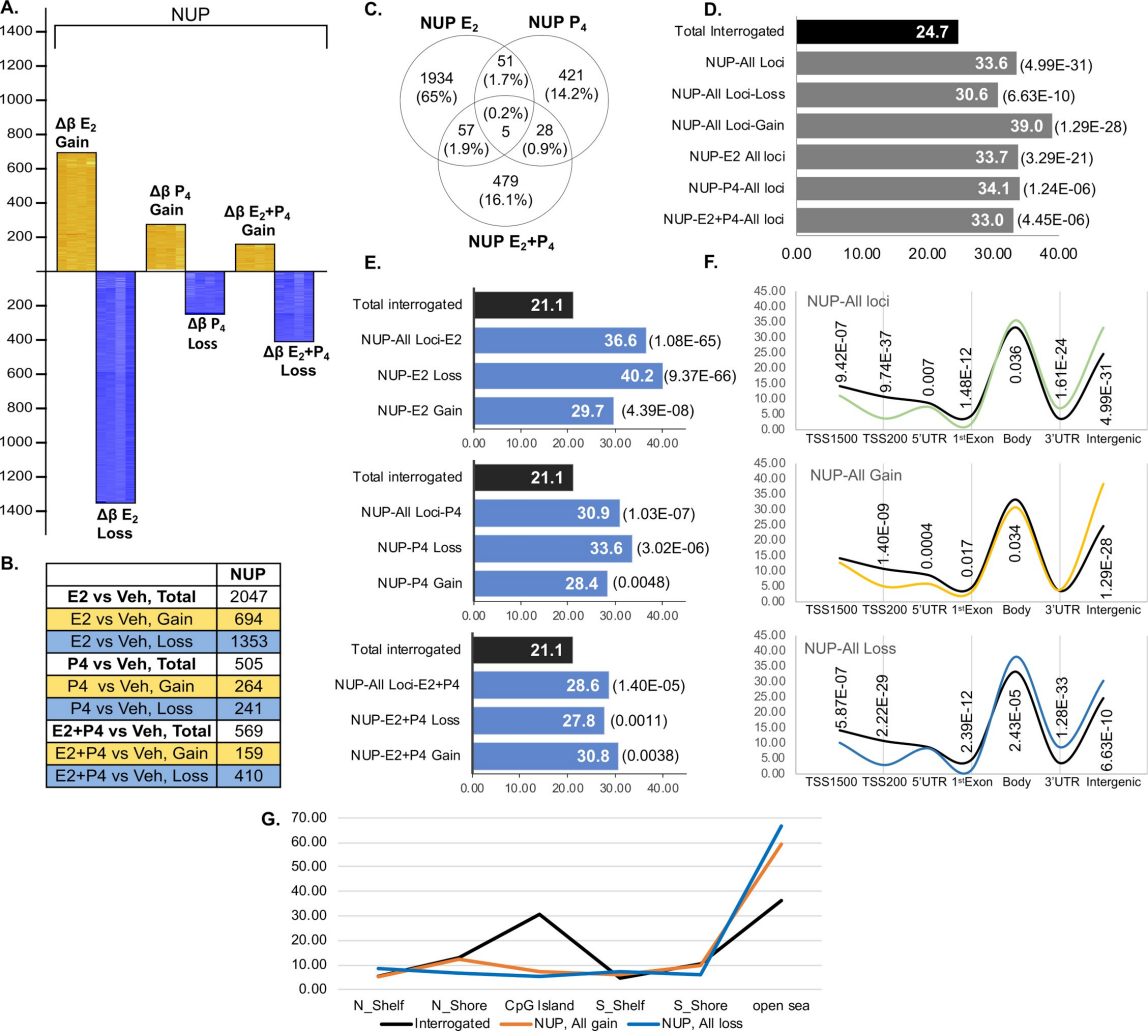
Hormone induced differentially methylated CpG sites in normal eSF (NUP). **1A.** Differentially methylated CpG sites induced by E_2_, P_4_ and E_2_+P_4_ versus vehicle. Each heatmap reflects differential methylation of each sample in each hormone treatment versus its corresponding non-treated vehicle control (Δβ: Hormone treated minus vehicle control). Yellow heatmaps above the X-axis reflect gain of methylation vs. vehicle; blue heatmaps below the X-axis reflect Δβ loss of methylation. In each heatmap, rows show Δβ of differentially methylated loci, columns indicate samples. Y-axis shows the number of differentially methylated loci for either gain or loss of methylation for each hormone treatment. **1B.** Number of differentially methylated CpG sites and in gain/loss of methylation for each hormone treatment. **1C.** Venn diagram of unique and common differentially methylated CpG sites for each hormone shows little overlap between differentially methylated loci in each hormone treatment **1D.** Enrichment of intergenic regions in % in each hormone treatment for all differentially methylated loci (All Loci), those with gain or loss of methylation (Gain, Loss) and by individual hormones (E_2_, P_4_, E_2_+P_4_). Enrichment is assessed by Z-test and p<0.05 are shown in parentheses. Black bar represents percentage of intergenic loci of total interrogated CpG sites. **1E.** Statistically significant involvement of enhancers by hormones and gain or loss of methylation. Enrichment is assessed by Z-test and p<0.05 are shown in parentheses. Black bar represents percentage of enhancers of total interrogated CpG sites. **1F.** Genomic distribution of all differentially methylated CpG sites in each hormone and by gain or loss of methylation, assessed at TSS1500, TSS200, 5’UTR, 1^st^ exon, gene body, 3’UTR, and intergenic regions. Black line represents the percentage of interrogated CpG site at each location, green line (top panel) shows all differentially methylated loci in NUP for all hormones, yellow line (middle panel) shows all loci with gain of methylation in NUP, and blue line (bottom panel) shows all loci with loss of methylation for all hormones. Enrichment is assessed by Z-test and p<0.05 are shown in parentheses for each genomic location. **1G.** Distribution of differentially methylated CpGs by CpG islands (CGI), CGI north/south shores and shelves for all loci with gain of methylation in all hormone treatments (orange lines) or with loss of methylation (blue line) in comparison to the distribution of the interrogated CpG sites in each of these genomic locations (black line). N Shelf: North Shelf; S Shelf: South Shelf; N Shore: North Shore; S Shore: South Shore. NUP: normal (no uterine pathology).

Interrogation of 485,577 methylation targets across the genome revealed that E_2_ and P_4_ and their combination affected the DNA methylome to different extents and with distinct patterns in eSF_normal_. (Note: throughout the Results section steroid responses of eSF DNA methylomes are compared to untreated (vehicle) cells for each group.) E_2_ induced the most extensive changes in the eSF_normal_ DNA methylome (2047 CpG sites), followed by combined E_2_+P_4_ (569 CpG sites), and P_4_ alone had the least effect (505 CpG sites) (**Fig 1A and 1B, S1 Table**). Importantly, combined E_2_+P_4_ resulted in dramatically reduced numbers of differentially methylated loci compared to E_2_ alone (**Fig 1A**). While individual hormone treatments elicited hormone-specific DNA methylome changes, the simultaneous presence of both hormones altered their individual effects. The pattern of loss and gain of methylation is also distinct for each hormone. E_2_, and E_2_+P_4_ induced more loss than gain of methylation (**Fig 1A,** yellow: gain of methylation, blue: loss of methylation vs vehicle), while P_4_ induced similar numbers of loss and gain of methylation. Concordant with differential patterns and extents of methylation changes, we found minimal overlap in the differentially methylated CpG sites affected by each hormone and the majority was unique (**Fig 1C**, **S1 Table**). In particular, loci differentially methylated in response to E_2_+P_4_ were mostly unique compared to those in response to P_4_ or E_2_ alone and were not a combination of the response to each hormone individually (**Fig 1C**).

Pathways and biofunctions (**Table 1**) as well as functional enrichment clustering **(Table 2)** were also unique to each hormone with E_2_ pathways enriching for gap junctions, melanogenesis, and glutamatergic and dopaminergic synapses pathways, and zinc and ion binding, cell membrane, glycoprotein and signal peptide functional clusters, with fewer and unique statistically significant pathways and functional clusters affected by P_4_ and E_2_+P_4_. Together these data indicate that each hormone affects different regions and E_2_+P_4_ targets are not a combination of E_2_ and P_4_. Differentially methylated loci in all hormonally treated eSF_normal_ involved several pathways, many important in normal endometrial function and dysfunction. Important pathways affected by each hormone and the differentially methylated genes in each pathway are shown in **S1 Data.** The data were further mined for differences in DNA methylation patterns, profiles, and genomic locations, regulatory elements, transcribed genes and biofunctions induced by each hormonal treatment in cells from normal and endometriosis women (see below).

**Table 1:** Pathways associated with differentially methylated (DM) transcribed loci in each hormonal treatment (E_2_, P_4_, E_2_+P_4_) vs. vehicle in normal (NUP), stage I (Endo I) and stage IV (Endo IV).

Pathways in each hormone treatment	NUP (P-Values <0.05, *Enriched but P>0.05*)	Endo I (P-Values <0.05, *Enriched but P>0.05*)	Endo IV (P-Values <0.05, *Enriched but P>0.05*)
E_2_ vs. Veh	Gap junction (0.015), Long-term potentiation (0.016), Long-term depression (0.025), Melanogenesis (0.035), Glutamatergic synapse (0.038), Dopaminergic synapse (0.041), *Sphingolipid signaling pathway (0.053), Ubiquitin mediated proteolysis (0.064), cGMP-PKG signaling pathway (0.07), Thyroid hormone signaling pathway (0.077), Retrograde endocannabinoid signaling (0.078), Cell adhesion molecules (CAMs) (0.08), mRNA surveillance pathway (0.092), Vascular smooth muscle contraction (0.097)*	MAPK signaling pathway (0.00052), cGMP-PKG signaling pathway (0.0014), PI3K-Akt signaling pathway (0.0019), Focal adhesion (0.0025), Axon guidance (0.0049), ECM-receptor interaction (0.0058), Oxytocin signaling pathway (0.012), Melanoma 0.015 Platelet activation (0.015), ErbB signaling pathway (0.017), Gap junction (0.018), Long-term depression (0.018), Amoebiasis (0.022), AMPK signaling pathway (0.022), Olfactory transduction (0.024), Renin secretion (0.025), Choline metabolism in cancer (0.039), Arrhythmogenic right ventricular cardiomyopathy (ARVC) (0.041), T cell receptor signaling pathway (0.044), Protein digestion and absorption (0.046), *VEGF signaling pathway (0.057), Cell adhesion molecules (CAMs) (0.058), Inflammatory bowel disease (IBD) (0.069), Proteoglycans in cancer (0.071), Renal cell carcinoma (0.074), HIF-1 signaling pathway (0.077), Ubiquitin mediated proteolysis (0.093), Regulation of actin cytoskeleton (0.098)*	Term P-Value Melanoma (0.00043), Signaling pathways regulating pluripotency of stem cells (0.00069), Proteoglycans in cancer (0.0018), Estrogen signaling pathway (0.012), Glioma (0.012), Choline metabolism in cancer (0.013), Olfactory transduction (0.022), ErbB signaling pathway (0.032), Prostate cancer (0.034), Endometrial cancer (0.035), Ras signaling pathway (0.037), GnRH signaling pathway (0.037), FoxO signaling pathway (0.038), Non-small cell lung cancer (0.042), *MAPK signaling pathway (0.064), Endocytosis (0.067), Cholinergic synapse (0.068), Fc epsilon RI signaling pathway (0.068), Oxytocin signaling pathway (0.068), Rap1 signaling pathway (0.071), Neurotrophic signaling pathway (0.085), RNA degradation (0.091), Pathways in cancer (0.095)*
P_4_ vs. Veh	*Staphylococcus aureus infection (0.071), MAPK signaling pathway (0.084), Tuberculosis (0.095), Tight junction (0.097)*	Pathways in cancer (0.015), Cell adhesion molecules (CAMs) (0.02), Glutamatergic synapse (0.028), Toxoplasmosis (0.032), Cocaine addiction (0.037), *Steroid hormone biosynthesis (0.056), Phototransduction (0.062), Inflammatory bowel disease (IBD) (0.071), Serotonergic synapse (0.086), Chronic myeloid leukemia (0.094)*	Non-small cell lung cancer (0.00031), Prostate cancer (0.00033), Glioma (0.00062), Endometrial cancer (0.0022), Thyroid hormone synthesis (0.0065), Melanoma (0.0069), Glutamatergic synapse (0.0073), Calcium signaling pathway (0.012), ErbB signaling pathway (0.014), MAPK signaling pathway (0.019), Proteoglycans in cancer (0.02), Estrogen signaling pathway (0.021), Acute myeloid leukemia (0.022), Pathways in cancer (0.024), Long-term depression (0.026), Cholinergic synapse (0.031), Pancreatic cancer (0.032), Amphetamine addiction (0.033), Chronic myeloid leukemia (0.042), Dopaminergic synapse (0.048), *FoxO signaling pathway (0.055), cAMP signaling pathway (0.06), Gap junction (0.068), Viral carcinogenesis (0.068), Hepatitis B (0.069) Adrenergic signaling in cardiomyocytes (0.071), Bladder cancer (0.071), GnRH signaling pathway (0.073), Rap1 signaling pathway (0.074), Oxytocin signaling pathway (0.089), Choline metabolism in cancer (0.094), Cocaine addiction (0.096)*
E_2_+ P4 vs Veh	Morphine addiction (0.022), *Steroid hormone biosynthesis (0.082), Long-term depression (0.089)*	Focal adhesion (0.00097), ECM-receptor interaction (0.0019), PI3K-Akt signaling pathway (0.0087), Glutamatergic synapse (0.0099), Toxoplasmosis (0.012), FoxO signaling pathway (0.024), Dorso-ventral axis formation (0.033), Arrhythmogenic right ventricular cardiomyopathy (ARVC) (0.038), *Hypertrophic cardiomyopathy (HCM) (0.053), Dilated cardiomyopathy (0.069), NF-kappa B signaling pathway (0.077)*	Transcriptional mis-regulation in cancer (0.012), PI3K-Akt signaling pathway (0.043), *AMPK signaling pathway (0.051), Epstein-Barr virus infection (0.066), Prostate cancer (0.079), Type I diabetes mellitus (0.083), Taste transduction (0.093)*

**Table 2:** Comparison of Functional Enrichment clusters in normal, stage I and stage IV in each hormone treatment (E_2_, P_4_, E_2_+P_4_) vs. vehicle.

	Normal	Stage I	Stage IV
**E**_**2**_ **Treatment**	4 clusters (Enrichment Score ≥2) 1) 255 loci: zinc/zinc ion binding, metal binding 2) 576 loci: disulfide bond, signal peptide, Cell membrane, Signal, Extracellular, Glycoprotein 3) 14 loci: TK 4) 19 loci: Spectrins, Spectrin/alpha-actinin, Calponin homology domain, CHs, Actin-binding, Actinin-type, DNA replication, recombination, and repair	6 clusters (Enrichment Score ≥2) 1) 449 loci: Cell membrane, cytoplasmic, glycoprotein, membrane, transmembrane 2) 13 loci: SAM 3) 48 loci: synapse/cell junction 4) 20 loci: Fibronectin types 5) 301 loci: disulfide bond, signal peptide, Signal, Glycoprotein 6) 16 loci: PDZ	Only 1 cluster (Enrichment Score ≥2) 1) 135 loci: signal peptide, Receptor, Signal, disulfide bond, Cell membrane, Extracellular, Cytoplasmic, plasma membrane, glycosylation site: N-linked, Glycoprotein, integral component of membrane, transmembrane region, Transmembrane helix, Membrane
**P**_**4**_ **Treatment**	2 (+ 1) clusters (Enrichment Score ≥2) 1) 18 loci: Pleckstrin homology domain, PH 2) 31 loci: PH1, PH2, guanyl-nucleotide exchange factor activity, regulation of small GTPase mediated signal transduction, positive regulation of apoptotic process, Dbl homology (DH) domain, RhoGEF, Rho guanyl-nucleotide exchange factor activity, regulation of Rho protein signal transduction, DH, Guanine-nucleotide releasing factor, positive regulation of GTPase activity, Pleckstrin homology-like domain 3) (ES = 1.99) 100 loci: topological domain: Cytoplasmic, Cell membrane, plasma membrane	4 clusters (Enrichment Score ≥2) 1) 25 loci: Synapse, cell junction 2) 34: Cadherins, cell adhesion 3) 149: in Extracellular, Cell membrane, plasma membrane, Cytoplasmic, glycosylation site: N-linked, transmembrane region, integral component of membrane, Transmembrane, membrane 4) 7: SAM	2 clusters (ES 2.9 and ES 1.99) 1) 24 loci: Cadherins, calcium ion binding, homophilic cell adhesion via plasma membrane adhesion molecules, Calcium, Cell adhesion 2) 14 loci: postsynaptic density, Postsynaptic cell membrane, cell junction, Synapse, Cell junction
**E**_**2**_**+P**_**4**_ **treatment**	NO clusters of ES≥2; 2 clusters of 1.61 1) 126 loci: transcription factor activity, sequence-specific DNA binding, DNA binding, regulation of transcription, DNA-templated, nucleoplasm, Transcription/T regulation, Nucleus 2) 3 loci: Diacylglycerol kinase, catalytic domain, ATP-NAD kinase-like domain, DAGKc	6 clusters (Enrichment Score ≥2) 1) 14: PDZ, PDZ domain 2) 49: EGF, EGF-like, EGF-like Ca binding 3) 21: ECM-receptor interaction; Focal adhesion; PI3K-Akt signaling pathway 4) 43: postsynaptic density, Postsynaptic cell membrane, Synapse, cell junction, 5) 24: Spectrins, Actin/actin-binding, Spectrin/alpha-actinin 6) 264:: Extracellular, cell membrane, Glycoprotein, transmembrane/ transmembrane region	E2/P4: 1 cluster (Enrichment Score ≥2) 1) 24 loci: Fibronectins, Immunoglobulin-like fold

### Genomic locations, regulatory elements, CpG islands and neighborhood context

Interestingly, while the patterns, profiles, differentially methylated CpG sites, pathways and biofunctions were unique to each hormone, the genome-wide distribution of their affected CpG sites shows specific enrichments and depletions. All hormones (E_2_, P_4_ and E_2_+P_4_) were statistically significantly enriched in intergenic regions (**Fig 1D)** and in enhancers (**Fig 1E)**, albeit with different extents and in gain vs loss of methylation and some variations based on hormones. These may reflect hormone binding sites in these regions, as had been reported in breast cancer cell lines [22,23]. There was a marked depletion of differential methylation in close proximity to transcription start sites (TSS) up to -200 nt upstream (TSS200) for all hormonal treatments in both of gain or loss of methylation (**Fig 1F**; **S2 Fig** for gain/loss for each hormone**; S2 Table**). But, CpG sites with gain of methylation in all hormonal treatments exhibited low enrichment at 5’UTRs, 1^st^ exons and gene bodies, while in loss of methylation TSS1500 and 1^st^ exons were less involved but gene bodies and 3’UTRs were more enriched. Greatest differences in gain versus loss of methylation (**Fig 1F**) involved gene bodies, and 3’UTRs, and less at TSS1500, TSS200, 5’UTRs, and 1^st^ exons.

*CpG islands (CGI)*, *CGI shores and shelves*. For all hormones, there was low involvement of CGIs and CGI shelves and shores, compared to interrogated loci on the HM450 platform (**Fig 1G** and **S3 Table**). Most DNA methylation changes in eSF_normal_ did not involve CGIs. Indeed, while 31% of all interrogated loci were at CGIs and 33% at CGI shores and shelves (total CGI-related 64%), only 5–7% of the differentially methylated loci for any hormonal treatment were located at CGIs (33–40% overall in CGIs, shores and shelves, compared to 64% arrayed on the platform). The majority of differentially methylated CpG sites involved non-CGIs (59–66%) versus 36% non-CGI CpG sites on the platform. However, there were more differentially methylated CpG sites at CGI shores and shelves than in CGIs, in both gain and loss of methylation. Notably, more CGIs, less CGI shelves (north and south shelves), more CGI shores (north and south shores), and less non-CGI CpG sites were involved in gain versus loss of methylation.

### Changes in gene expression in response to hormones

Steroid hormones affect their target genes through various mechanisms and as such, changes in DNA methylation may not fully reflect their effect on changes in gene expression, particularly in the case of those loci whose transcriptional regulation does not involve chromatin modifiers. To elucidate a more complete effect of hormones, transcriptomic profiles were determined in the same steroid hormone-treated eSF used for DNA methylation analysis and was compared to its corresponding gene expression profiles in untreated eSF. E_2_ induced more up- than down-regulated genes (**Table 3,** top up- and down-regulated loci; **S4 Table,** full gene list), and P_4_ elicited similar numbers of up- and down-regulated genes, (**S4 Table**). However, more genes were differentially expressed when E_2_ and P_4_ were combined, with more genes up- than down-regulated (**S4 Table**).

**Table 3:** Top up/down regulated differentially expressed genes in response to each hormone treatment (E_2_, P_4_, E_2_+P_4_) vs. vehicle in normal (NUP), stage I (Endo I) and stage IV (Endo IV).

Transcripts Cluster Id	Gene symbol	Fold change	Regulation	Chr.	Entrez gene	Gene Description
**NUP_E2 vs Veh**						
7965873	IGF1	21.46	up	chr12	3479	insulin-likegrowthfactor1(somatomedin C)
8040292	GREB1	8.19	up	chr2	9687	growthregulationbyestrogeninbreastcancer1
7951165	**PGR**	4.78	up	chr11	5241	Progesterone receptor
7971461	LCP1	4.70	up	chr13	3936	lymphocytecytosolicprotein1(L-plastin)
8101659	SPARCL1	4.60	up	chr4	8404	SPARC-like1(hevin)
8088560	ADAMTS9	3.81	up	chr3	56999	ADAMmetallopeptidasewiththrombospondintype1motif,9
7987315	ACTC1	3.79	up	chr15	70	actin, alpha, cardiacmuscle1
8107823	ADAMTS19	3.13	up	chr5	171019	ADAMmetallopeptidasewiththrombospondintype1motif,19
8086352	ULK4	3.00	up	chr3	54986	unc-51likekinase4
8080562	IL17RB	2.91	up	chr3	55540	interleukin17receptorB
7965335	DUSP6	-2.08	down	chr12	1848	dualspecificityphosphatase6
7917561	GBP4	-2.09	down	chr1	115361	guanylatebindingprotein4
8146863	SULF1	-2.11	down	chr8	23213	sulfatase1
8097692	EDNRA	-2.18	down	chr4	1909	Endothelin receptor type A
7921916	RGS5	-2.25	down	chr1	8490	regulatorofG-proteinsignaling5
7968417	FRY	-2.32	down	chr13	10129	Furry homolog (Drosophila)
7909503	SERTAD4	-2.41	down	chr1	56256	SERTAdomaincontaining4
8067969	CHODL	-2.46	down	chr21	140578	chondrolectin
8006433	CCL2	-2.84	down	chr17	6347	chemokine(C-C motif) ligand2
7906919	RGS4	-3.58	down	chr1	5999	regulatorofG-proteinsignaling4
**NUP_P4 vs Veh**						
8089145	ABI3BP	14.27	up	chr3	25890	ABI family, member3(NESH)binding protein
8101659	SPARCL1	13.19	up	chr4	8404	SPARC-like1(hevin)
8155864	RORB	5.17	up	chr9	6096	RAR-related orphan receptor B
7965873	IGF1	4.61	up	chr12	3479	insulin-likegrowthfactor1(somatomedin C)
7977933	SLC7A8	4.31	up	chr14	23428	solutecarrierfamily7(amino acid transporter light chain, L system), member 8
8138231	THSD7A	4.11	up	chr7	221981	thrombospondin, type I, domaincontaining7A
8132694	IGFBP1	3.87	up	chr7	3484	insulin-likegrowthfactorbindingprotein1
7971461	LCP1	3.70	up	chr13	3936	lymphocytecytosolicprotein1(L-plastin)
8122099	ENPP1	3.22	up	chr6	5167	Ectonucleotide pyrophosphatase/phosphodiesterase1
8043995	IL1R1	3.21	up	chr2	3554	interleukin1receptor, type I
8046048	CSRNP3	-2.07	down	chr2	80034	cysteine-serine-richnuclearprotein3
7923978	CD34	-2.09	down	chr1	947	
8166747	SYTL5	-2.10	down	chrX	94122	synaptotagmin-like5
7945680	H19	-2.17	down	chr11	283120///100033819///6206	H19, imprintedmaternallyexpressedtranscript(non-proteincoding)|microRNA675|ribosomalproteinS12
7917561	GBP4	-2.25	down	chr1	115361	guanylatebindingprotein4
8150428	SFRP1	-2.29	down	chr8	6422	secretedfrizzled-relatedprotein1
8146863	SULF1	-2.30	down	chr8	23213	sulfatase1
8102587	NDNF	-2.31	down	chr4	79625	neuron-derived neurotrophic factor
8138289	ETV1	-2.66	down	chr7	2115	etsvariant1
8006433	CCL2	-2.73	down	chr17	6347	Chemokine (C-C motif) ligand2
**NUP_E2+P4 vs Veh**						
8101659	SPARCL1	53.10	up	chr4	8404	SPARC-like1(hevin)
8089145	ABI3BP	37.91	up	chr3	25890	ABI family, member3 (NESH) binding protein
8132694	IGFBP1	24.11	up	chr7	3484	insulin-likegrowthfactorbindingprotein1
8155864	RORB	17.01	up	chr9	6096	RAR-related orphan receptor B
8040292	GREB1	15.10	up	chr2	9687	growthregulationbyestrogeninbreastcancer1
7977933	SLC7A8	13.95	up	chr14	23428	solutecarrierfamily7(amino acid transporter light chain, L system), member 8
7965873	IGF1	13.61	up	chr12	3479	insulin-likegrowthfactor1(somatomedin C)
8138231	THSD7A	10.44	up	chr7	221981	thrombospondin, type I,domaincontaining7A
8144917	LPL	8.89	up	chr8	4023	Lipoprotein lipase
7971461	LCP1	8.88	up	chr13	3936	lymphocytecytosolicprotein1(L-plastin)
8055323	NCKAP5	-3.31	down	chr2	344148	NCK-associatedprotein5
8121916	RSPO3	-3.39	down	chr6	84870	R-spondin3
8129573	MOXD1	-3.49	down	chr6	26002	monooxygenase, DBH-like1
7917561	GBP4	-3.80	down	chr1	115361	guanylatebindingprotein4
7945680	H19	-3.95	down	chr11	283120///100033819///6206	H19, imprinted maternally expressed transcript (non-protein coding) |microRNA675|ribosomalproteinS12
8138289	ETV1	-4.12	down	chr7	2115	etsvariant1
8131803	IL6	-4.34	down	chr7	3569	interleukin6
8006433	CCL2	-5.52	down	chr17	6347	Chemokine (C-C motif) ligand2
8150428	SFRP1	-6.05	down	chr8	6422	secretedfrizzled-relatedprotein1
7933194	CXCL12	-6.08	down	chr10	6387	chemokine(C-X-C motif) ligand12
**Endo I_E2 vs Veh**						
7965873	IGF1	11.37	up	chr12	3479	insulin-likegrowthfactor1(somatomedin C)
7951165	**PGR**	3.82	up	chr11	5241	Progesterone receptor
8117020	MYLIP	3.34	up	chr6	29116	Myosin regulatory light chain interacting protein
8145766	NRG1	2.94	up	chr8	3084	neuregulin1
8111490	PRLR	2.82	up	chr5	5618	Prolactin receptor
8102950	INPP4B	2.77	up	chr4	8821	inositolpolyphosphate-4-phosphatase, type II,105kDa
7942674	TSKU	2.67	up	chr11	25987	tsukushi, small leucine rich proteoglycan
8043995	IL1R1	2.55	up	chr2	3554	interleukin1receptor, type I
8088560	ADAMTS9	2.45	up	chr3	56999	ADAMmetallopeptidasewiththrombospondintype1motif,9
8106516	JMY	2.26	up	chr5	133746	Junction mediating and regulatory protein, p53 cofactor
8135069	SERPINE1	-1.61	down	chr7	5054	Serpin peptidase inhibitor, clade E (nexin,plasminogenactivatorinhibitortype1),member1
7902565	LPHN2	-1.66	down	chr1	23266///101927458	latrophilin2|uncharacterizedLOC101927458
7921916	RGS5	-1.74	down	chr1	8490	regulatorofG-proteinsignaling5
7909503	SERTAD4	-1.74	down	chr1	56256	SERTAdomaincontaining4
7968417	FRY	-1.75	down	chr13	10129	Furry homolog(Drosophila)
7922343	TNFSF4	-1.79	down	chr1	7292	Tumor necrosis factor(ligand)superfamily,member4
8006433	CCL2	-1.90	down	chr17	6347	chemokine(C-C motif) ligand2
7906919	RGS4	-2.02	down	chr1	5999	regulatorofG-proteinsignaling4
8021081	SLC14A1	-2.37	down	chr18	6563	solutecarrierfamily14(urea transporter), member1(Kidd blood group)
7922337	TNFSF18	-2.53	down	chr1	8995	Tumor necrosis factor(ligand) superfamily, member18
**Endo I_P4 vs Veh (All DE loci)**						
7964834	CPM	2.06	up	chr12	1368	Carboxypeptidase M
8089145	ABI3BP	1.90	up	chr3	25890	ABI family, member3(NESH) binding protein
8043995	IL1R1	1.89	up	chr2	3554	interleukin1receptor, type I
8157524	TLR4	1.88	up	chr9	7099	toll-likereceptor4
8101659	SPARCL1	1.83	up	chr4	8404	SPARC-like1(hevin)
7907271	FMO2	1.83	up	chr1	2327	flavincontainingmonooxygenase2(non-functional)
7933204	C10orf10	1.65	up	chr10	11067	chromosome10openreadingframe10
7965873	IGF1	1.65	up	chr12	3479	insulin-likegrowthfactor1(somatomedin C)
8122660	UST	1.59	up	chr6	10090	uronyl-2-sulfotransferase
8052355	EFEMP1	1.55	up	chr2	2202	EGFcontainingfibulin-likeextracellularmatrixprotein1
7969861	ITGBL1	1.51	up	chr13	9358	integrin, beta-like1(with EGF-like repeat domains)
8100154	CORIN	1.50	up	chr4	10699	corin, serine peptidase
7922337	TNFSF18	-1.56	down	chr1	8995	Tumor necrosis factor (ligand)superfamily, member18
**Endo I_E2+P4 vs Veh**						
8101659	SPARCL1	23.12	up	chr4	8404	SPARC-like1(hevin)
7965873	IGF1	12.49	up	chr12	3479	insulin-likegrowthfactor1(somatomedin C)
8089145	ABI3BP	10.15	up	chr3	25890	ABI family, member3 (NESH) binding protein
8155864	RORB	8.92	up	chr9	6096	RAR-related orphan receptor B
8147030	STMN2	7.62	up	chr8	11075	stathmin2
8043995	IL1R1	6.73	up	chr2	3554	interleukin1receptor, type I
8125919	FKBP5	5.05	up	chr6	2289	FK506bindingprotein5
7977933	SLC7A8	4.04	up	chr14	23428	solutecarrierfamily7(amino acid transporter light chain, L system), member 8
8122099	ENPP1	4.04	up	chr6	5167	Ectonucleotide pyrophosphatase/phosphodiesterase1
7964834	CPM	3.96	up	chr12	1368	Carboxypeptidase M
8138289	ETV1	-2.32	down	chr7	2115	etsvariant1
7921916	RGS5	-2.34	down	chr1	8490	regulatorofG-proteinsignaling5
8081001	ROBO2	-2.34	down	chr3	6092	roundabout,axonguidancereceptor,homolog2(Drosophila)
8136248	MEST	-2.44	down	chr7	4232	Mesoderm specific transcript
7922343	TNFSF4	-2.51	down	chr1	7292	Tumor necrosis factor (ligand)superfamily, member 4
7906919	RGS4	-2.76	down	chr1	5999	regulatorofG-proteinsignaling4
8006433	CCL2	-3.14	down	chr17	6347	chemokine(C-C motif) ligand 2
7922337	TNFSF18	-3.29	down	chr1	8995	Tumor necrosis factor (ligand) superfamily, member 18
8021081	SLC14A1	-3.33	down	chr18	6563	solutecarrierfamily14(urea transporter), member 1(Kidd blood group)
7945680	H19	-3.62	down	chr11	283120///100033819///6206	H19,imprintedmaternallyexpressedtranscript(non-proteincoding)|microRNA675|ribosomalproteinS12
**Endo IV_E2 vs Veh**						
7965873	IGF1	12.71	up	chr12	3479	insulin-likegrowthfactor1(somatomedin C)
7951165	**PGR**	5.19	up	chr11	5241	Progesterone receptor
8117020	MYLIP	4.13	up	chr6	29116	Myosin regulatory light chain interacting protein
8111490	PRLR	3.44	up	chr5	5618	Prolactin receptor
8102950	INPP4B	2.88	up	chr4	8821	inositolpolyphosphate-4-phosphatase, typeII,105kDa
8040292	GREB1	2.77	up	chr2	9687	growthregulationbyestrogeninbreastcancer1
8107823	ADAMTS19	2.71	up	chr5	171019	ADAMmetallopeptidasewiththrombospondintype1motif,19
8088560	ADAMTS9	2.51	up	chr3	56999	ADAMmetallopeptidasewiththrombospondintype1motif,9
8145361	NEFM	2.48	up	chr8	4741	neurofilament, medium polypeptide
8106516	JMY	2.43	up	chr5	133746	Junction mediating and regulatory protein, p53 cofactor
7920123	S100A10	-1.94	down	chr1	6281	S100calciumbindingproteinA10
8131803	IL6	-2.03	down	chr7	3569	interleukin6
8135218	LRRC17	-2.11	down	chr7	10234	leucinerichrepeatcontaining17
7906919	RGS4	-2.15	down	chr1	5999	regulatorofG-proteinsignaling4
7921916	RGS5	-2.15	down	chr1	8490	regulatorofG-proteinsignaling5
7997139	CALB2	-2.20	down	chr16	794	calbindin2
8021081	SLC14A1	-2.23	down	chr18	6563	solutecarrierfamily14(urea transporter), member 1(Kidd blood group)
7922343	TNFSF4	-2.51	down	chr1	7292	Tumor necrosis factor (ligand) superfamily, member4
7922337	TNFSF18	-2.58	down	chr1	8995	Tumor necrosis factor(ligand) superfamily, member 18
8006433	CCL2	-3.30	down	chr17	6347	chemokine(C-C motif) ligand 2
**Endo IV_P4 vs Veh (All DE loci)**						
7907271	FMO2	1.99	up	chr1	2327	flavincontainingmonooxygenase2(non-functional)
7908459	CFH	1.93	up	chr1	3075	Complement factor H
7965873	IGF1	1.85	up	chr12	3479	insulin-likegrowthfactor1(somatomedin C)
8043995	IL1R1	1.82	up	chr2	3554	interleukin1receptor, type I
8157524	TLR4	1.61	up	chr9	7099	toll-likereceptor4
7961514	MGP	1.59	up	chr12	4256	Matrix Gla protein
8089145	ABI3BP	1.58	up	chr3	25890	ABI family, member3 (NESH)binding protein
7933204	C10orf10	1.58	up	chr10	11067	chromosome10openreadingframe10
8101659	SPARCL1	1.51	up	chr4	8404	SPARC-like1(hevin)
8111490	PRLR	1.50	up	chr5	5618	Prolactin receptor
8111941	HMGCS1	-1.49	down	chr5	3157	3-hydroxy-3-methylglutaryl-CoAsynthase1(soluble)
**Endo IV_E2+P4 vs Veh**						
8101659	SPARCL1	29.40	up	chr4	8404	SPARC-like1(hevin)
8089145	ABI3BP	17.62	up	chr3	25890	ABI family, member3 (NESH)binding protein
7965873	IGF1	17.30	up	chr12	3479	insulin-likegrowthfactor1(somatomedin C)
8122099	ENPP1	10.98	up	chr6	5167	Ectonucleotide pyrophosphatase/phosphodiesterase1
8155864	RORB	10.33	up	chr9	6096	RAR-related orphan receptor B
7977933	SLC7A8	8.57	up	chr14	23428	solutecarrierfamily7(amino acid transporter light chain, L system), member8
8043995	IL1R1	8.54	up	chr2	3554	interleukin1receptor, type I
7908459	CFH	6.96	up	chr1	3075	Complement factor H
8138231	THSD7A	6.18	up	chr7	221981	thrombospondin, type I, domaincontaining7A
8125919	FKBP5	6.12	up	chr6	2289	FK506bindingprotein5
7997139	CALB2	-3.00	down	chr16	794	calbindin2
8150428	SFRP1	-3.12	down	chr8	6422	secretedfrizzled-relatedprotein1
7922337	TNFSF18	-3.26	down	chr1	8995	Tumor necrosis factor (ligand) superfamily, member 18
7917561	GBP4	-3.27	down	chr1	115361	guanylatebindingprotein4
8055323	NCKAP5	-3.36	down	chr2	344148	NCK-associatedprotein5
8138289	ETV1	-3.59	down	chr7	2115	etsvariant1
8021081	SLC14A1	-3.81	down	chr18	6563	solutecarrierfamily14 (urea transporter), member1(Kidd blood group)
7922343	TNFSF4	-4.00	down	chr1	7292	Tumor necrosis factor (ligand) superfamily, member4
7906919	RGS4	-4.21	down	chr1	5999	Regulator of G-proteinsignaling4
8006433	CCL2	-6.47	down	chr17	6347	Chemokine (C-C motif)ligand2

E_2_+P_4_ induced the largest and P_4_ the smallest changes in gene expression. Almost all the genes differentially induced by P_4_ were shared with E_2_+P_4_ and some were shared with the E_2_ treatment. Notably, half of the E_2_-induced and the majority of E_2_+P_4_ induced differentially expressed genes were unique. However, in commonly up-regulated genes between E_2_ and E_2_+P_4_, the variable fold changes indicate inhibitory or stimulatory effects when E_2_ is combined with P_4_. For example, *PGR* is upregulated by both E_2_ and E_2_+P_4_ (FC = 4.5 vs 1.7, respectively), indicating that the addition of P_4_ limited up-regulation of *PGR* compared to E_2_ alone. Among other up-regulated gene in common in E_2_, P_4_, E_2_+P_4_, are *IGF1* and *SPARCL1* with known roles in endometrial biology. But, the FCs were different (*IGF1*: E_2_ = 21.5, P_4_ = 4.6, E_2_+P_4_ = 13.6; *SPARCL1*: E_2_ = 4.6, P_4_ = 13.2; E_2_+P_4_ = 53.1) indicating potentially different mechanisms for up- or down-regulation and for different genes, potentially affected by genomic location and other regulatory factors, modifiers and gene/region-specific mechanisms involved in hormone-induced gene expression regulation.

#### Pathways and biofunctions

E_2_ increased tissue and cellular development, growth and maintenance, and downregulated cell-to-cell signaling, immune cell trafficking, inflammatory response, apoptosis and cellular migration (**S5 Table**). P_4_ elicited down-regulation of cellular regeneration and proliferation and cell-cell signaling and adhesion. E_2_+P_4_ upregulated cell death and molecular transport and downregulated cell growth and proliferation, carbohydrate metabolism and molecular transport. The genes commonly upregulated by E_2_, P_4_, E_2_+P_4_ involved catalytic activity, receptor and signal transduction, binding, transporter and structural molecule activity. The main biofunctions of differentially expressed genes that were shared with differentially methylated loci involved cell membrane and signaling in response to E_2_.

### Association of gene expression with DNA methylation

Hormonally-induced differentially methylated CpG sites were assessed for association with differential gene expression for each corresponding locus, noting that not all transcribed loci are included in both platforms and many intergenic regions in DNA methylation platform were not represented on the gene expression array used in this study. Only loci with a strong positive or negative association (by Spearman rho, and corrected p<0.05, see Methods) were considered. There was a large number of functional gene clusters with strong association of DNA methylation and gene expression for E_2_ in eSF_normal_ (**Table 5**), which was not observed for P_4_ or E_2_+P_4_ treatments.

### Effects of Ovarian Steroid Hormones in *Endometrium of Women with Endometriosis*

**Fig 2:**
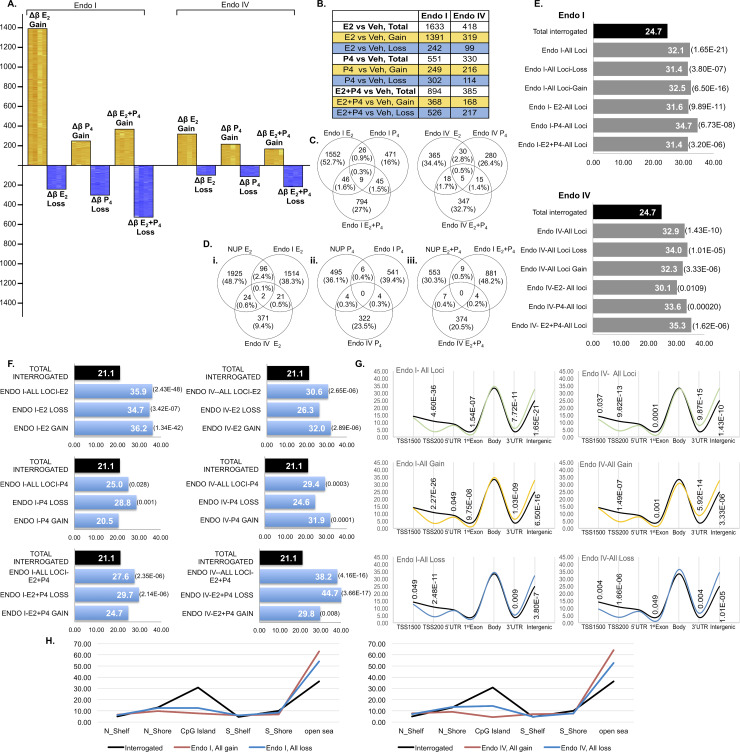
Hormone induced differentially methylated CpG sites in stage I eSF (Endo I) and stage IV eSF (Endo IV). **2A.** Differentially methylated CpG sites induced by E_2_, P_4_ and E_2_+P_4_ versus vehicle. Heatmaps reflect the differential methylation of each sample in each hormone treatment versus its corresponding non-treated vehicle control (Δβ: Hormone treated minus vehicle control) (see Fig 1A legend for details). **2B.** Number of differentially methylated CpG sites and in gain/loss of methylation for each hormone treatment in Endo I and Endo IV. **C.** Unique and common differentially methylated CpG sites for each hormone in Endo I (left) and Endo IV (right) indicating mostly unique loci for each hormone. 2**D**. Unique and common differentially methylated CpG sites across normal (NUP), Endo I and Endo IV, for each hormone: E_2_: left, P_4_: middle and E_2_+P_4_: right. **2E.** Enrichment of intergenic regions; Endo I, top and Endo IV bottom charts (see Fig 1D legend for details). **2F.** Enrichment of enhancers for each hormone and based on loss or gain of methylation in Endo I (left panel) and Endo IV (right panel) (see Fig 1E legend for details). **2G.** Genomic distribution of all differentially methylated CpG sites in each group (Endo I, left, Endo IV right panel) and by gain or loss of methylation (see Fig 1F legend for details). **2H.** Distribution of differentially methylated CpGs by CpG islands (CGI), CGI north/south shores and shelves for Endo I (left) and Endo IV (right) based on gain or loss of methylation for all hormones. For details see Fig 1G legend. N Shelf: North Shelf; S Shelf: South Shelf; N Shore: North Shore; S Shore: South Shore. Endo I: stage I; Endo IV: stage IV.

We next aimed to determine the effect of hormones on the endometrium of endometriosis patient, known to have abnormal P_4_ response. We applied strict criteria using eSF from patients with only endometriosis and no other uterine, pelvic or gynecologic disorders and those that show P_4_-resistance confirmed by microscopy and IGFBP1 assay (**S1 Fig**). Furthermore, to understand the effect of disease stage on the hormone-epigenome interplay, we used early (stage I) and late stage (stage IV) disease. Similar to normal, E_2_ induced the most and P_4_ the least DNA methylation changes in eSF_stage-I_ and eSF_stage-IV_ (**Fig 2A and 2B, S6 and S7 Tables**, respectively). But, in eSF_endo_, the extent of E_2_-induced changes was less than in eSF_normal_ specifically in eSF_stage-IV_ (418 CpG sites) exhibiting significantly less methylation alterations compared to eSF_normal_ and eSF_stage-I_ (2047 and 1633 CpG sites, respectively) (**Fig 2B)**. Opposite to that of eSF_normal_ the majority of changes in both stages were gain of methylation (**Fig 2A** heatmap). The extent of E_2_-induced differentially methylated loci differed considerably between the two stages (**Fig 2A and 2B**): stage I showed extensive changes induced by E_2_, much reduced in stage IV. The considerable difference in the extent of E_2_- induced methylation in eSF_stage-IV_ and in the gain/loss pattern (**Fig 2A and 2B**), indicate an aberrant response to E_2_ in both stages of disease and more extensively in stage IV, not previously reported. Progesterone, similar to eSF_normal_ induced the least DNA methylome alterations in both eSF_stage-I_ and eSF_stage-IV_ despite the difference in the robust decidualization response to P_4_ in eSF_normal_ and the refractory decidualization response to P_4_ in eSF_endo_ (**S1 Fig)**. While P_4_ induced similar numbers of loss and gain of methylation in eSF_normal_ in eSF_stage-I_ there was more loss than gain of methylation and in eSF_stage-IV_ more gain than loss of methylation. Interestingly, despite complete lack of decidualization, eSF_stage-I_ exhibited more E_2_+P_4_-induced differentially methylated loci versus eSF_normal_, and in both disease stages there was more loss than gain of methylation (**Fig 2A and 2B**). eSF_stage-IV_ showed the fewest methylome changes in all three hormone treatments, suggesting extensive aberrancies in hormone-methylome interactions in late stage disease. Particularly important is the novel observation of an aberrant response to E_2_ and not just to P_4_ and E_2_+P_4,_ as previously believed [24].

Differentially methylated loci were unique in response to different hormones in each disease stage and between the two stages. (**Fig 2C**). As in normal, E_2_+P_4_ induced methylation were mostly unique and not a combination of the response to E_2_ or P_4_ individually (**Fig 2C**), reaffirming that E_2_ and P_4_ interact differently with the epigenome when combined than when individually administered (**Fig 2C, S6 and S7 Tables**). Moreover, the majority of loci differentially methylated in response to each specific hormone was also unique in eSF_normal_ vs eSF_stage-I_ vs eSF_stage-IV_ (**Fig 2D**). These data suggest that the hormone-DNA methylome dynamics differ under normal and disease conditions, and furthermore that the stage of disease affect the hormone-methylome response.

Despite distinct profile differences with normal, hormone-induced differentially methylated CpGs for E_2_, P_4_ and E_2_+P_4_in both stages of eSF_endo_ were also statistically significantly enriched in intergenic regions (**Fig 2E**) although the extent of this enrichment differed among hormone treatments, and between disease stages (**Fig 2E**, **S2 Fig** for gain/loss for each hormone**; S2 Table**). Similar to normal and in both gain and loss of methylation, there was marked enrichment of enhancers, although the extent differed with specific hormone treatments and disease stage (**Fig 2F**). In both E_2_ and P_4_ treatment, eSF_normal_ involved more enhancers than eSF_stage-I_ and eSF_stage-IV_, but E_2_+P_4_ treatment induced involvement of more enhancers in eSF_stage-IV_, particularly in loss of methylation with nearly 50% of CpGs associated with enhancers (**Fig 2F**).

In eSF_stage-I_, the genomic distribution of loci with gain or loss of methylation differed from eSF_normal_ at 1^st^ exons, gene bodies, and 3’UTRs. In eSF_stage-IV_ the genomic distribution was mostly similar in gain and loss of methylation. These differed at 5’UTRs, gene bodies and 3’UTRs compared to eSF_normal_ and at 5’UTRs and 1^st^ exons compared to eSF_stage-I_ (**Fig 2G**). Overall, these data demonstrate that: hormone treatments regardless of disease and its stage affected CpG sites more at the 3’UTR and intergenic regions and much less at proximal promoters/TSS; genomic locations of CpG sites differentially methylated in response to hormones differed based on loss/gain of methylation; while decreased proximal promoter (TSS200) involvement and increased intergenic region involvement were common in eSF_normal,_ eSF_stage-I_ and eSF_stage-IV_. Low involvement of promoters/TSS and increased involvement of 3’UTR and intergenic regions were remarkable, considering vast differences in patterns, profiles and loci differentially methylated in eSF under the different hormonal treatments and in normal versus disease. These observations underscore key roles for genomic locations and potentially chromatin configurations further directing hormonal effects.

*CpG islands (CGI)*, *CGI shores and shelves*. There was low involvement of CGIs and CGI shelves and shores in both disease stages **Fig 2H** (and **S3 Table**), similar to normal. But, in both disease stages loss of methylation involved more CGIs than gain of methylation.

### Pathways and biofunctions associated with differentially methylated loci

Interestingly, hormone treatments significantly enriched more pathways in eSF_stage-I_ and eSF_stage-IV_ versus eSF_normal_ (**Table 1**) (**S1 Data** shows important pathways affected by each hormone in each eSF group and marking differentially methylated genes in those pathways*)*. Thus, while there were fewer loci in eSF_stage-I_ and eSF_stage-IV_ compared to eSF_normal_, more specific pathways were affected; whereas, with eSF_normal_ hormone effects did not particularly affect specific canonical pathways and likely involved broader targets across the genome. E_2_ affected pathways in eSF_stage-I_ involved endometrial function/dysfunction and endometriosis (e.g. MAPK, PI3K-Akt, ErbB signaling, focal adhesion, gap junctions, among others (**Table 1**)). E_2_ elicited pathways in eSF_stage-IV_ associated with proteoglycans and estrogen, ErbB, Ras, GnRH, and FoxO signaling (**Table 1**)–all relevant to endometriosis pathophysiology [8,14]. While P_4_ did not significantly enrich specific pathways in eSF_normal,_ indicating a more genome-wide effect instead of limited effect at specific canonical pathways, several statistically significant pathways were enriched in eSF_stage-I_ and even more in eSF_stage-IV_. These data suggest an aberrant response to P_4_ in eSF from women with disease, which is enhanced in stage IV (**Table 1**) involving specific pathways including estrogen, MAPK and ErbB signaling, confirming pathways associated with transcriptomic data [19,20]. Similarly, in response to E_2_+P_4_, there were more enriched pathways in eSF_endo_ than in eSF_normal_ relevant to endometrial function (adhesion, and disease/cancer (**Table 1**)).

Gene functional enrichments in eSF_normal_, eSF_stage-I_ and eSF_stage-IV_ for each hormone treatment are shown in **Table 2.** While there was little overlap in genes or functional clusters in eSF_endo_ compared to eSF_normal_, the greatest number of genes in the same functional cluster in all eSF groups induced by E_2_ involved those with signal peptide, membrane, and glycoprotein functions. eSF_stage-I_ had more gene functional clusters with specific functions in all hormone treatments compared to eSF_stage-IV_ or eSF_normal_. eSF_stage-IV_ had the fewest functional clusters in all treatments compared to eSF_stage-I_ and eSF_normal_, and the genes affected specific pathways involved in endometriosis and cancer as observed in the pathway analysis (above). Importantly, P_4_ treatment affected specific gene functions in disease (adhesion, synapse, cell junction, cadherins), different from eSF_normal_. E_2_+P_4_ elicited, only in eSF_stage-I_, several distinct functional clusters with specific functions in endometrial biology and endometriosis, including EGF/EGF-like genes, ECM receptor interaction, focal adhesion, PI3K-Akt pathway, synapse, cell junctions, spectrins and others. The most enriched cluster elicited by E_2_+P_4_ in eSF_stage-IV_ included fibronectins (large glycoproteins in ECM that bind integrins and other matrix components with major roles in cell adhesion, growth, migration differentiation, fibrosis and cancer).

Functional enrichment differences did not show a gradual change from eSF_normal_ to eSF_stage-I_ and then to eSF_stage-IV_, rather showed distinct enrichments, suggesting inherent differences between disease stages. These data support that stage I and stage IV belong to distinct disease subtypes.

### Aberrant hormone-induced methylation in eSF_endo_ are due to pre-existing methylation abnormalities

As patterns, profiles, pathways and gene functions differed in responses of eSF_stage-I,_ and eSF_stage-IV_ to E_2_, P_4_ and E_2_+P_4_ in comparison to those of eSF_normal,_ the question arose whether these could be due, in part, to aberrant DNA methylation signatures present *prior to* hormone treatment (referred to “pre-existing differences” herein). The DNA methylation status of untreated (vehicle) eSF_endo_ from women with endometriosis were assessed for loci with aberrant methylation changes in response to each hormone, compared to eSF_normal_ and whether they differed from untreated (vehicle) eSF_normal_ (see Methods). Numerous aberrantly differentially methylated loci in disease were found to be due to *pre-existing* DNA methylation differences across the genome (**S3 Fig**), including up to 53% of aberrant E_2_+P_4_ loss of methylation in eSF_stage-IV_, showing an aberrant methylation pattern from that of the untreated normal eSF, further resulting in aberrant response to hormone treatments. These data are supported by previous gene expression analysis of eSF_normal_ and eSF_endo_ at t = 0 in culture [19], demonstrating intrinsic and pre-existing abnormalities in the eSF_endo_ cells, although unclear whether these aberrancies are due to disease or are contributing to its progression/pathogenesis.

### Changes in gene expression in response to hormones

As in eSF_normal_, transcriptomic profiles were determined for both stages of disease for each hormone (versus control). Whether these responses were abnormal was investigated compared to normal. In eSF_endo_, all treatments resulted in fewer differentially expressed genes versus eSF_normal_ (**Table 3, S8 and S9 Tables)**. P_4_ alone induced the fewest differentially expressed genes in eSF_stage-I,_ and eSF_stage-IV_, consistent with the DNA methylation changes, an aberrant response to P_4_ and abnormal decidualization in eSF from women with endometriosis (**Fig 2A**) [18,19]. In general, genes differentially expressed in response to each hormonal treatment were different in each disease stage versus normal, although some were in common (**Table 4**). Of interest to endometrial function and in disease, *PGR* was also upregulated in response to E_2_ in eSF_stage-I,_ and eSF_stage-IV_ similar to eSF_normal_ (**Table 4**) and was also among the top up-regulated genes in all groups despite the aberrant and very limited P_4_ response observed in the DNA methylation, gene expression and IGFBP1 production in eSF from women with disease (**Table 3, S1 Fig**). E_2_, P_4_ and E_2_+P_4_ up-regulated *IGF1* and IL1R1 in all eSF and *SPARCL1* was up-regulated in all E_2_+P_4_ treated eSF (**Table 4, S10 Table** (full list and Venn diagrams of unique and common genes within each group and each hormone treatment across groups)). Gene expression profiles in response to hormones, similar to the DNA methylome, demonstrated distinct and aberrant molecular signatures in eSF_stage-I_ versus eSF_stage-IV_ and compared to eSF_normal_. Moreover, these were not limited to P_4_ and E_2_+P_4_ treatments and, importantly, included an abnormal response of eSF_stage-I_ and eSF_stage-IV_ to E_2._

**Table 4:** Differentially expressed loci common in each hormone treatment across normal (NUP), stage I (Endo I) and stage IV (Endo IV).

E2-induced upregulated loci common in NUP_Endo I_ Endo IV	E2-induced downregulated loci common in NUP_Endo I_ Endo IV	P4-induced upregulated loci common in NUP_Endo I_ Endo IV	E2+P4-induced upregulated loci common in NUP_Endo I_Endo IV		E2+P4-induced downregulated loci common in NUP_Endo I_ Endo IV
IGF1*^	NUAK1	ABI3BP	SPARCL1	SLC29A1	LIMCH1
GREB1^	NCAM1	SPARCL1	ABI3BP	ZBTB16	ROBO2
PGR^	SERPINE1	IGF1*	RORB	RAB31^	KRT18
LCP1^	TNFSF18	IL1R1*	GREB1^	KLF6	PIK3R3
ADAMTS9	SLC14A1U***	C10orf10	SLC7A8	PEMT	PTCHD4
ADAMTS19	FLT1	TLR4	**IGF1**^	RGS9	RASSF2
IL17RB	TNFSF4		THSD7A	PCBP3	GALNT5
SNCA^	MEST***		LCP1^	ITPR1	FRY***
ISOC1	LMO7***		FBXO32	AHNAK2	RNA5SP104
MYLIP	EDNRA***		MAOB	MAP3K4	FAM46C
TSKU^	RGS5***		FKBP5	SOD2	TYMS
NRG1^	FRY***		ENPP1	CNTN3	LYPD1
SEMA6A	SERTAD4***		CRISPLD2	ADAMTS1	SLC14A1***
GUCY1A2	CCL2***		ULK4	SPTSSA	ARHGAP18
PRLR^	RGS4***		**IL1R1**^	SORBS1	PLK2
PRICKLE2^			IMPA2	TSC22D3	ENC1
JMY			MUM1L1	LAMA3	SULF1
MIR503			GPX3	ITGBL1	MYO1B
KLF4			STMN2	SYTL4	SYTL5
IL1R1*^			CRYAB	PMP22	ATP8B1
AFAP1L2			SPSB1	SH3PXD2B	AMIGO2
ASPN^			TMEM37	MFGE8	UGCG
INPP4B			CPM	PIK3R1	KRTAP1-5
PXK			GALNT15	NID1	PLEKHG1
TMEM120B			MYOCD	EVA1C	GPR39
MIR503HG			ST6GALNAC2	APOD	LOXL4**
CPXM1			THBD	ABCC9	ATP2B1
LIN7A			FAM134B	CD151	TMEM130
NEFM			LPAR1	EPS8	MGAT5
BMP2			GPRC5B	SEPP1	GLT8D2
SLC35F6^			PRLR^	LAMA2	CCDC14
FAM102A			CERS6	FOXO1	MEST***
CPZ			MGST1	ARHGAP20	PPP1R3C
OSBPL3			ABHD5	PPAP2B	RARRES2
SNCAIP			C10orf10	NRG1^	RASGRF2
LOXL4**			CD68	TXNIP	PGRMC1
RNU2-6P			CXCR4	RAP2A	ACKR4
ACER3			GLULP4	FBLN2	LMO7***
RAB31^			EFEMP1	ETS2	GBP2
PAPSS2			SESTD1	TMOD1^	ARHGAP29
ASCC3			NLGN4X	HAND2-AS1	SERTAD4***
TMOD1^			PRUNE2	PTGER2	CSRNP3
C16orf45			OLFML2B	LHFP	GABRE
CDH4			SLC40A1	PPP1R14A	FJX1
			HAND2	LAMB1	RGS5***
			ADAMTS2	TLR4	FHOD3
			TSKU^	LMCD1	CD200
			GLUL	RAP1B	TNFRSF19
			ABLIM3	CFH	DUSP6
			CORIN	YBX3	PTN
			ACSL1	PGR^	TGFBI
			IRS2	RPS6KA2	KRT19
			MEDAG	SLC35F6^	F2RL2
			HOMER1	PPP1R3B	DACH1
			ADRA2C	NPC1	NCAM2
			DPT	PLIN2	MXRA5
			ASPN^	PRICKLE2^	RGS4***
			MOB3B	HSPA2	EDNRA***
			NFIL3	DPP4	NCKAP5
			INSR	ITGB1BP1	MOXD1
			ARRDC4	PQLC3	GBP4
			DKK1	APCDD1	H19
			SNCA^	ENDOD1	ETV1
			SRPX	ANTXR2	IL6
			UST	ANG	CCL2***
			ELMO1	GRIA1	SFRP1

* in both common E2 and common P4 up-regulated genes

^ in both E2 and E2+P4 up-regulated; **BOLD,** in E2, P4 and E2+P4 common loci

** Up regulated in E2 and down regulated in E2+P4

*** Down-regulated in E2 and E+P. *NOTE*: *There are no P4-induced downregulated loci in common in NUP*, *Endo I*, *Endo IV*.

### Changes in gene expression in stage I disease

In eSF_stage I_, similar to eSF_normal_, all hormone treatments resulted in more gene up-regulation than down-regulation, but unlike eSF_normal_, all hormones, including E_2_ affected fewer differentially expressed genes, with a marked minimal effect with P_4_ (**S8 Table**, full gene list; **S10 Table**, common genes and Venn diagrams). Also, in E_2_ and in E_2_+P_4_ half and the majority of the genes, respectively, were unique, but in P_4_, the majority of differentially expressed genes were in common with E_2_+P_4_. Note that the number of P_4_-induced differentially expressed genes were very limited in stage I, while the combination of E_2_+P_4_ in stage I disease resulted in more differentially expressed genes than with E_2_ or P_4_ treatments alone (**S8 Table**). Similar to eSF_normal_, where addition of E_2_ minimally affected P_4_ target genes, E_2_ combined with P_4_ affected the target genes of E_2_ alone. While there were commonly upregulated P_4_ target genes eSF_stage I_ and eSF_normal_ including *IGF1*, *GREB1*, and *PGR*, *many key genes were missing in* eSF_stage I_, such as, *SPARCL1* which was upregulated in normal but not in stage I disease, further indicating an aberrant E_2_ response in disease.

### Changes in gene expression in stage IV

Similar to eSF_stage I_ the number of differentially expressed genes in response to E_2_ as well as to P_4_ treatments were far fewer than what was observed in eSF_normal_ (**S9 Table**, full gene list). Similar to eSF_normal_ and eSF_stage I_ there were more differentially expressed genes by E_2_+P_4_. Among commonly up-regulated E_2_+P_4_ induced eSF_stageIV_ and eSF_normal_ were *SPARCL1*, *IGF1*, and *LAMA3*. Among the 103 genes commonly down-regulated were *CCL2*, *RGS4*, *RGS5*, *IL-6*, *MEST*, *KRT19*, *KRT18 and H19*. The overlap in differential expression of specific up- and down-regulated genes with E_2_+P_4_ treatment of eSF from women with and without endometriosis is remarkable, since stage IV disease eSF cells did not decidualize and are not considered to be P_4_-responsive [24,25].

### Pathways and biofunctions

Pathways and biofunctions, derived from the gene expression data, underscored distinct differences between eSF_endo_ and eSF_normal_ and between stages of disease, similar to the DNA methylation data. In eSF_stage-I,_ with more limited E_2_ effects, pathways included activation of cellular proliferation and viability (**S5 Table**), and eSF_stage-IV_ involved increased tissue and cellular development (as with eSF_normal_), proliferation, cell-cell signaling and adhesion (unlike eSF_normal_). Note that E_2_-induced biofunctions and pathways were different in eSF_stage-I_ and eSF_stage-IV_ and both differed from eSF_normal_ (**S5 Table**), consistent with the DNA methylation data. There were no enriched pathways in eSF_stage-I_ and moderately enriched (Z score = 1.9) up-regulation of cell growth and proliferation in eSF_stage-IV_ in reponse to P4 (far fewer loci). Similar to eSF_normal_, carbohydrate metabolism and molecular transport was also seen in eSF_stage-I_ in response to E_2_+P_4_, which also showed up-regulation of cell invasion and viability. Importantly, E_2_+P_4_ increased cell survival, cell movement and invasion, cell-to-cell signaling and adhesion, and downregulated cellular proliferation and growth in eSF_stage-IV_. Genes involved in these pathways and their upstream regulators are shown in **S5 Table**. Similar to normal, the main biofunctions of differentially expressed genes that were shared with differentially methylated loci involved cell membrane and signaling in response to E_2_ in eSF_stage-IV_.

### Association of gene expression with DNA methylation

In loci with a strong positive or negative association of DNA methylation and gene expression (by Spearman rho, and corrected p<0.05) distinct differences were found in eSF_stage-I_ and eSF_stage-IV_ and versus eSF_normal_ (full lists, **S11–S13 Tables; S14 Table** for unique and common loci between each group). Functional enrichment analyses revealed distinct differences in numbers and types of gene functional clusters in each stage of disease versus normal. While there was a large number of functional gene clusters with strong association for E_2_ in eSF_normal_ (**Table 5**), eSF_stage-I_ and eSF_stage-IV_ showed different and more limited functional clusters. This result further indicates that the E_2_ response is aberrant in eSF_endo_ compared to normal eSF.

**Table 5:** Functional enrichment cluster analysis for loci with strong association of differential methylation and gene expression changes in each hormone treatment in normal (NUP), stage I (Endo I) and stage IV (Endo IV) disease.

Enrichment score>2 and P<0.05	E_2_ vs. Veh	P_4_ vs. Veh	E_2_+P_4_ vs. Veh
NUP, pos/neg rho (gain and loss)	Cluster 1: Spectrins, Spectrin/alpha-actinin, Actinin-type, actin-binding, Calponin homology (CH) domain, Cell division and chromosome partitioning, DNA replication, recombination, and repair. Cluster 2: ATP-binding, Protein kinase-like domain, protein kinase activity, Nucleotide-binding, protein phosphorylation, intracellular signal transduction, Serine/threonine-protein kinase. Cluster 3: Pleckstrin homology-like domain, PH. Cluster 5: Metal-binding, Zinc, Zinc-finger, zinc ion binding. Cluster 6: Cell adhesion, Cadherins. Cluster 7: Biological rhythms, regulation of circadian rhythm, rhythmic process. Cluster 8: postsynaptic membrane, Cell junction, Synapse. Cluster 9: GTPase activator activity, Rho GTPase activation protein, regulation of small GTPase mediated signal transduction. Cluster 10: Fibronectins. Cluster 11: zinc finger region: ZZ-type.	Cluster 1: Dynein heavy chain/domain, microtubule motor activity. cluster 2; Zinc, zinc ion binding, Zinc-finger, Metal-binding.	No functional cluster enrichment with significant p-value and enrichment score>2.
Endo I, pos/neg rho, (gain and loss)	Cluster1: cell adhesion, cadherins, calcium ion channel. Cluster 2: a number of Sushi domains. Cluster 3: SAM. Cluster 4: ATP-binding, nucleotide phosphate-binding region: ATP, Nucleotide-binding, Kinase.	cluster 1: homophilic cell adhesion via plasma membrane adhesion molecules, Cadherins, Cell adhesion, calcium ion binding, CA. Cluster 2: Synapse, Cell junction, Postsynaptic cell membrane. Cluster 3: regulation of small GTPase mediated signal transduction, positive regulation of GTPase activity, Rho GTPase activation protein, signal transduction. Cluster 4: Sterile alpha motif domain (SAM). Cluster 5: regulation of small GTPase mediated signal transduction, Dbl homology (DH) domain, Rho guanyl-nucleotide exchange factor activity, Pleckstrin homology-like domain, positive regulation of apoptotic process, Src homology-3 domain, intracellular signal transduction.	Cluster 1: PDZ/PDZ domain. Cluster2: EGF, EGF-like, EGF-like calcium-binding, Laminin, Insulin-like growth factor binding protein, TB domain, Extracellular matrix, Secreted. Cluster 3: Calponin homology (CH) domain. Cluster 4: Cell junction, Synapse, Postsynaptic cell membrane, neuron projection.
Endo IV, pos/neg rho (gain and loss)	Cluster 1: plasma membrane, topological domain: Extracellular, Glycoprotein, Signal, Disulfide bond, Transmembrane. Cluster 2: Neurexin/syndecan/glycophorin C, cell adhesion.	No enrichment in functional clusters (total probes = 106) with our stat cut off, but some enrichment without ES>2 include: cluster 1 at 1.69: Fibronectins, Laminin, cell adhesion, extracellular matrix, secreted. Cluster 2: 1.13: cell adhesion, glycoprotein, cell membrane, signal.	cluster 1: repeat I, II, III, IV (this includes these: calcium voltage-gated channel subunit alpha1 C(CACNA1C), calcium voltage-gated channel subunit alpha1 A(CACNA1A), integrin subunit beta like 1(ITGBL1). *(cluster 2: 1.8: Phosphotyrosine interaction domain. Cluster 3 (1.56):Pleckstrin homology-like domain. Cluster 4: (1.4): synapse, cell junction)*.

There were also multiple differences in response to P_4_, and E_2_+P_4_ among the eSF groups, further highlighting distinct molecular signatures in each disease stage. Importantly, eSF_stage-I_ showed distinct clusters in response to P_4_ and to E_2_+P_4,_ including functions characteristic of endometrial biology and endometriosis pathophysiology (**Table 5**).

While there were no statistically significantly enriched gene functional clusters in response to P4 in stage IV disease, a moderate enrichment of fibronectins, cell adhesion and secreted proteins were noted. These are consistent with the important role for cell adhesion in stage I and stage IV disease. In response to E_2_+P_4_, eSF_stage-IV_ showed enrichment of calcium channels and integrins. Together these data suggest that the responses to all hormone treatments are aberrant in eSF derived from women with stage I and stage IV disease and are specific to each stage, supporting distinct disease subtypes.

### Comparison of *in vitro* versus *in vivo* data

Herein, eSF hormone treatments *in vitro* were chosen to approximate the hormonal milieu *in vivo* (E_2_-dominant proliferative phase endometrium (PE) and E_2_+P_4_-dominant mid-secretory phase endometrium (MSE)). Comparing *in vitro* hormone eSF transcriptome data to corresponding phases in bulk endometrial tissue in normal versus disease [26] and FACS-isolated eSF_endo_ and eSF_normal_ [20] revealed great overlap of differentially expressed genes (**Table 6, S15 and S16 Tables**). GO functional analysis of genes differentially expressed in E_2_ treated eSF_stage-IV_ and PE tissue revealed many genes in common involved in regulation of cell migration and motility, proteolysis, negative regulation of cell death, regulation of fibroblast proliferation and others. Regulation of inflammatory response, cell migration/motility, transport, protein import to nucleus, signal transduction, wound healing, and epithelial development and others were noted in stage I (**S15 Table**).

**Table 6:** Genes commonly regulated in eSF treated with E_2_ and E_2_+P_4_ and in whole endometrial tissue in PE and MSE or in FACS sorted eSF.

**eSF E**_**2**_ **treated in common with WHOLE Tissue PE in Disease vs. Control**						
Gene Symbol	In Vitro array.Probe.Set. ID	FC ([Endo IV-E] vs [NUP-E])	FC ([Endo I-E] vs [NUP-E])	FC ([PE.Endo] vs [PE.Control])	Tissue array. Probe.Set.ID	Gene Title
SPARCL1	8101659	-2.86	-4.51	-2.77	200795_at	SPARC-like 1 (hevin)
CXCL12	7933194	-2.79	-2.02	-2.20	203666_at	chemokine (C-X-C motif) ligand 12
GREB1	8040292	-2.77	-3.49	-1.66	205862_at	growth regulation by estrogen in breast cancer 1
IL1R1	8043995	-2.53	-1.76	-2.30	202948_at	interleukin 1 receptor, type I
COLEC12	8021946	-2.23	-2.02	-2.33	221019_s_at	collectin sub-family member 12
LCP1	7971461	-2.12	-2.66	-1.73	208885_at	lymphocyte cytosolic protein 1 (L-plastin)
FAM134B	8111136	-2.09	-1.82	-2.82	218532_s_at	family with sequence similarity 134, member B
IGF1	7965873	-2.07	-2.76	-2.68	209540_at	insulin-like growth factor 1 (somatomedin C)
ADAMTS9	8088560	-2.02	-2.06	-3.77	226814_at	ADAM metallopeptidase with thrombospondin type 1 motif, 9
SLC40A1	8057677	-2.01	-2.25	-1.98	223044_at	solute carrier family 40 (iron-regulated transporter), member 1
PDE7B	8122222	-1.97	-2.00	-2.44	230109_at	phosphodiesterase 7B
PLIN2	8160297	-1.94	-1.65	-2.18	209122_at	perilipin 2
IL17RB	8080562	-1.93	-2.36	-2.27	224156_x_at	interleukin 17 receptor B
SPTLC2	7980438	-1.91	-1.72	-5.38	203128_at	serine palmitoyl transferase, long chain base subunit 2
CD109	8120719	-1.88	-1.49	-4.35	226545_at	CD109 molecule
TMX4	8064939	-1.85	-1.61	-2.67	201580_s_at	thioredoxin-related transmembrane protein 4
CCBE1	8023575	-1.85	-1.59	-1.83	243805_at	collagen and calcium binding EGF domains 1
FAM46A	8127778	-1.84	-1.68	-1.90	224973_at	family with sequence similarity 46, member A
EPHA5	8100578	-1.83	-1.86	-2.42	237939_at	EPH receptor A5	
SPON1	7938608	-1.82	-1.84	-2.13	213994_s_at	spondin 1, extracellular matrix protein
ABCG2	8101675	-1.81	-1.94	-3.01	209735_at	ATP-binding cassette, sub-family G (WHITE), member 2
RSPO3	8121916	-1.75	-1.92	-2.21	228186_s_at	R-spondin 3 homolog (Xenopus laevis)
CLIC2	8176234	-1.74	-1.54	-2.68	213415_at	chloride intracellular channel 2
SLC18A2	7930837	-1.68	-1.78	-2.58	205857_at	solute carrier family 18 (vesicular monoamine), member 2
PRICKLE2	8088550	-1.67	-1.48	-2.83	225968_at	prickle homolog 2 (Drosophila)
KLHL13	8174654	-1.61	-1.51	-2.31	227875_at	kelch-like 13 (Drosophila)
PLA2R1	8056151	-1.59	-1.51	-1.51	207415_at	phospholipase A2 receptor 1, 180kDa
PLD1	8092134	-1.57	-1.47	-1.59	226636_at	phospholipase D1, phosphatidylcholine-specific
WT1	7947363	-1.53	-1.82	-2.49	216953_s_at	Wilms tumor 1
C17orf58	8017831	-1.48	-1.45	-1.87	226901_at	chromosome 17 open reading frame 58
TWISTNB	8138454	-1.46	-1.65	-2.81	226784_at	TWIST neighbor
LONRF2	8054281	-1.46	-2.44	-3.55	225996_at	LON peptidase N-terminal domain and ring finger 2
SERPINE1	8135069	1.45	1.65	3.39	1568765_at	serpin peptidase inhibitor, clade E (nexin, plasminogen activator inhibitor type 1), member 1
IER3	8124848	1.48		5.13	201631_s_at	immediate early response 3
EIF4A1	8004506	2.01	1.60	1.89	214805_at	Eukaryotic translation initiation factor 4A1
TBRG4	8139482	2.12	1.73	2.93	220789_s_at	transforming growth factor beta regulator 4
IGFBP5	8058857	3.28	3.24	2.45	203425_s_at	insulin-like growth factor binding protein 5
IER3	8178435		1.47	5.13	201631_s_at	immediate early response 3
**eSF E2+P4 treated in common with WHOLE Tissue MSE in Disease vs Control**						
	In Vitro array.Probe.Set. ID	FC ([Endo IV-EP] vs [NUP-EP])	FC ([Endo I-EP] vs [NUP-EP])	FC ([MSE.Endo] vs [MSE.Control])	Tissue array. Probe.Set.ID	Gene Title
LPL	8144917	-4.54	-5.48	-1.62	203548_s_at	lipoprotein lipase	
LGR4	7947199	-3.35	-3.41	-3.15	218326_s_at	leucine-rich repeat-containing G protein-coupled receptor 4
GREB1	8040292	-3.19	-4.97	-2.10	205862_at	growth regulation by estrogen in breast cancer 1
F3	7917875	-2.92	-2.11	-2.50	204363_at	coagulation factor III (thromboplastin, tissue factor)
CAB39L	7971590	-2.64	-2.33	-2.62	225915_at	calcium binding protein 39-like
SPARCL1	8101659	-2.28	-2.92	-1.91	200795_at	SPARC-like 1 (hevin)	
GPR155	8056837	-2.24	-2.50	-1.63	239533_at	G protein-coupled receptor 155
FAM134B	8111136	-2.23	-2.76	-2.14	218532_s_at	family with sequence similarity 134, member B
RORB	8155864	-2.19	-2.73	-2.93	242385_at	RAR-related orphan receptor B
THSD7A	8138231	-2.10	-4.77	-2.28	214920_at	thrombospondin, type I, domain containing 7A
ADAMTS9	8088560	-2.09	-2.27	-2.74	226814_at	ADAM metallopeptidase with thrombospondin type 1 motif, 9
PRLR	8111490	-2.03	-2.67	-2.91	206346_at	prolactin receptor
LPAR1	8163257	-2.02	-2.14	-1.76	204037_at	lysophosphatidic acid receptor 1
ATP13A3	8092849	-2.02	-2.22	-1.58	212297_at	ATPase type 13A3
ADAMTS5	8069689	-1.99	-2.11	-3.14	229357_at	ADAM metallopeptidase with thrombospondin type 1 motif, 5
SAT1	8166469	-1.95	-2.20	-2.66	213988_s_at	spermidine/spermine N1-acetyltransferase 1
APOO	8171823	-1.94	-1.78	-2.29	221620_s_at	apolipoprotein O
CNKSR2	8166355	-1.91	-1.97	-1.79	229116_at	connector enhancer of kinase suppressor of Ras 2
ABHD5	8079153	-1.74	-1.91	-2.59	213935_at	abhydrolase domain containing 5
CTSS	7919800	-1.67	-1.56	-1.79	202902_s_at	cathepsin S
ANGPT2	8149071	-1.66	-1.65	-1.86	205572_at	angiopoietin 2
CADM1	7951807	-1.64	-1.87	-3.47	209032_s_at	cell adhesion molecule 1
ZCCHC6	8162147	-1.63	-1.87	-2.25	220933_s_at	zinc finger, CCHC domain containing 6
SESTD1	8057394	-1.63	-1.95	-2.31	226763_at	SEC14 and spectrin domains 1
KAL1	8171248	-1.62	-1.91	-1.97	205206_at	Kallmann syndrome 1 sequence
ETS2	8068593	-1.60	-1.46	-1.56	201328_at	v-ets erythroblastosis virus E26 oncogene homolog 2 (avian)
TSPAN12	8142524	-1.54	-1.52	-2.14	219274_at	tetraspanin 12
MDM1	7964810	-1.51	-1.58	-3.31	213761_at	Mdm1 nuclear protein homolog (mouse)
TSPAN13	8131600	-1.51	-1.52	-1.69	217979_at	tetraspanin 13
TMEM133	7943369	-1.48	-1.52	-1.77	223595_at	transmembrane protein 133
ARRDC4	7986350	-1.47	-1.90	-2.41	225283_at	arrestin domain containing 4
FERMT2	7979204	-1.45	-1.89	-2.29	209209_s_at	fermitin family member 2
TBRG4	8139482	1.99	1.57	2.35	220789_s_at	transforming growth factor beta regulator 4
**eSF signature in Disease vs Control in E2, or E2+P4 treated eSF common with FACS sorted eSF in Disease vs Control**						
**Compared to E2 treatment**						
	In Vitro array.Probe.Set. ID	FC ([Endo IV-E] vs [NUP-E])	FC ([Endo I-E] vs [NUP-E])	FC eSF FACS Endo vs eSF FACS control	Gene Title	
TNXB|TNXA	8179935	-2.53	-1.83	-2.32	tenascin XB | tenascin XA pseudogene	
LCP1	7971461	-2.12	-2.66	-2.12	lymphocyte cytosolic protein 1 (L-plastin)	
FAM134B	8111136	-2.09	-1.82	-1.64	family with sequence similarity 134, member B	
IL17RB	8080562	-1.93	-2.36	-2.06	interleukin 17 receptor B	
TGFBR3	7917649	-1.80	-1.57	-1.97	transforming growth factor, beta receptor III	
ABI3BP	8089145	-1.76	-2.10	-1.91	ABI family, member 3 (NESH) binding protein	
KLHL13	8174654	-1.61	-1.51	-1.55	kelch-like 13 (Drosophila)	
SNORD46	7901048	1.90	1.74	1.76	small nucleolar RNA, C/D box 46	
SERTAD4	7909503	2.28	2.04	1.54	SERTA domain containing 4	
RGS4	7906919	2.36	2.89	1.90	regulator of G-protein signaling 4	
SULF1	8146863	3.79	2.31	1.61	sulfatase 1	
**Compared to E2+P4 Treatment**						
	In Vitro array.Probe.Set. ID	FC ([Endo IV-EP] vs [NUP-EP])	FC ([Endo I-EP] vs [NUP-EP])	FC eSF FACS Endo vs eSF FACS control	Gene Title	
ABI3BP	8089145	-2.36	-6.50	-1.91	ABI family, member 3 (NESH) binding protein	
ADAMTS5	8069689	-1.99	-2.11	-1.87	ADAM metallopeptidase with thrombospondin type 1 motif, 5	
CRISPLD2	7997642	-1.58	-1.88	-1.55	cysteine-rich secretory protein LCCL domain containing 2	
FAM134B	8111136	-2.23	-2.76	-1.64	family with sequence similarity 134, member B	
KIAA0040	7922474	-2.46	-2.33	-1.50		
LCP1	7971461	-2.12	-2.90	-2.12	lymphocyte cytosolic protein 1 (L-plastin)	
MAOB	8172204	-2.24	-3.23	-1.54	monoamine oxidase B	
PARM1	8095751	-1.90	-1.56	-2.30	prostate androgen-regulated mucin-like protein 1	
SERTAD4	7909503	1.67	1.71	1.54	SERTA domain containing 4	
SNORD46	7901048	2.16	1.60	1.76	small nucleolar RNA, C/D box 46	
SULF1	8146863	2.12	1.67	1.61	sulfatase 1	
TMEM37	8044813	-1.61	-2.23	-1.54	transmembrane protein 37	

Comparing MSE tissue and E_2_+P_4_-treated eSF_stage-I_ and eSF_stage-IV_ vs normal, pathway analysis revealed common signaling pathways involving PI3K-Akt, Rap1 and Ras, and cancer (**S15 Table**).

Comparing transcriptomes of cultured eSF_endo_ and eSF_normal_ and freshly isolated (uncultured) FACS-sorted eSF_endo_ and eSF_normal_ from human eutopic endometrium [20] revealed many shared genes (**Table 6**; **S16 Table**). Note that FACS-sorted eSF_normal_ and eSF_endo_ included samples from various cycle phases, different disease stages [20], and a more limited sample number compared to the bulk tissue study. Thus, the number of overlapping loci in cultured and freshly isolated eSF is expectedly smaller than those shared with whole tissue.

Together, the extent of overlap with whole tissue samples and FACS isolated eSF indicates the *in vitro* hormonal treatment of eSF, the predominant cell type in endometrium, is a good model and reflects a persistent eSF signature in the whole tissue.

### Histone H3K27me3 and H3K27ac modifications in response to E_2_

Since E_2_ induced the largest changes in the DNA methylome of eSF, we sought to assess its effect on the histone marks to better understand how E_2_ affected the regulatory function of the epigenetic machinery. We assessed silencing and activating histone modifications, H3K27me3, and H3K27ac, using chromatin immunoprecipitation followed by deep sequencing (ChIP-Seq). Modifications of H3K27me3, and H3K27ac have been found in loci involved in eSF decidualization [10,11,27]. In response to E_2_ we observed more differential peaks in H3K27ac than H3K27me3 in line with our observation of more loss of DNA methylation corresponding to a more open chromatin state induced by E_2_ **(S17 Table** for peaks associated with each histone mark**).** GO gene functional analysis for each histone modification renriched pathways related to regulation of signaling, cell morphogenesis and differentiation, G-protein coupled receptor signaling, regulation of mitotic cell cycle and intracellular protein transport among others, many of which are shared with DNA methylation data **(S17 Table** for pathways for each histone mark**).**

### Association of PGR target loci identified in E_2_+cAMP+MPA decidualization and E_2_+P_4_ induced DNA methylation

Increased binding of PGR to open chromatin was shown previously in decidualizing cells by ChIP-Seq experiments [10,27] and that the presence of PGR binding site and its putative co-regulator FOSL2 in a genomic location is associated with open chromatin during decidualization [10,11]. Moreover, direct PGR targets in eSF treated for 72hrs with E_2_+MPA+cAMP were identified by Mazur *et*. *al*., using ChIP-Seq and RNA-Seq [10]. We assessed the overlap of the E_2_+P_4_ induced differentially methylated CpG sites associated with genes in normal, stage I and stage IV disease to genes with PGR binding sites present in the Mazur *et*. *al*. study within the extended promoter region (as defined to be -7500bp and +2500bp from TSS) and intervals within ±10KB, as well as ±25KB from transcriptional start/stop site in normal eSF. We found a small subset of genes overlapping in normal eSF (**Table 7**); however, these common genes were enriched for biofunctions that are involved in cell morphogenesis, differentiation and cell projections, endosome organization and cytoskeletal organization (**Table 7**). These are important during decidualization as eSF decidualization is characterized by morphological changes, expansion/restructuring of extracellular matrix, surface projections and expansion of endoplasmic reticulum. Interestingly, a larger number of genes in stage I and IV overlapped with PGR binding sites than normal eSF (**Table 7**). Stage IV and normal shared more common genes with PGR binding sites than they did with stage I (**Table 7**). Biofunction analysis showed more biofunctions involved in stage I than normal, such as tissue morphogenesis, response to TGF-beta signaling, response to growth factor and extracellular matrix among others (**Table 7**). In stage IV, the biofunctions involved negative regulation of Wnt signaling, and intracellular signal transduction (**Table 7**) known to be affected in endometriosis. These data further support the notion of aberrant P_4_ response, rather than P_4_-resistance in endometriosis.

**Table 7:** Differentially methylated (DM) genes with PGR binding sites affected by E_2_+P_4_ treatment in normal eSF (NUP), stage I eSF (Endo I) and stage IV eSF (Endo IV) and the associated pathways by GO analysis in DAVID.

**DM genes with PGR binding sites in E2+P4 treated NUP**	**DM genes with PGR binding sites in E2+P4 treated Endo I**	**DM genes with PGR binding sites in E2+P4 treated Endo IV**
*AFF1*, *ASH1L*, *C3orf21*, *CAP2*, *ELK3*, *CLSTN2*, *LOC442459*, *MYH13*, *MYLK4*, *NSMCE2*, *NUBPL*, *PHACTR1*, *RBM46*, *SAV1*, *SOX5*, *SPAG16*, *TBC1D1*, *TMEM232*, *TOP2B*, *WDR27*, *WWC3*, NLK***, TTL*** ATP8A2, C1QTNF9B, C8orf44, CACNA2D3, CDC73, CSRNP3, FMN1, GPM6B, HSD17B2, INPP5A, JPH1, KIAA1033, MCF2, MGAT4C, NEK10, ODZ2, OXR1, PIK3C3, PLXNC1, PTPRQ, RAB3C, SEMA5A, SGIP1, SNX25, SRGAP1, TBC1D4, TNFRSF9, VENTXP1, VTA1, WBP4, YEATS4, RBM20*, SRBD1*	ACTG2, ANKRD11, AP4E1, ARHGAP12, ARHGAP15, ATF6, BCAS1, C13orf26, C15orf61, CDH6, CLASP2, COL1A2, CPEB4,CROCC, CSTF3, DIRC3, DOPEY1, DSEL, EBF1, EYS, FARP1, FBN1, FBN3, FBXL2, FIGN, FLJ43860, FRAS1, FSIP1, GLI3, GLIS1, GNG7, GPR143, GPR176, GRID2, HERC2, HHLA2, HTRA3, ICA1L, INTS3, IQGAP2, IQGAP3, ITGA8, ITGB5, KCTD9, KDM4B, KIF26B, LOC285692, LRBA, LRRC8C, LTBP2, MAGI2, MED13L, METAP2, MYH8, NFIB, NHS, NOTCH4, NTM, PACSIN3, PCNX, PCNXL2, PDE3B, PLCG2, PPM1H, PRIM2, PTBP2, PTPN4, PTPRG, RGS7, RHOQ, ROBO1, RPS6KA2, RTKN2, SHROOM3, SLC44A1, SLIT3, SNTB1, SYNPR, TBX4, TGFBR3, THSD4, THSD7B, TNC, TRIO, UTRN,VWA3B, ZDHHC6, RBM20*, SRBD1*, NLK***, TTL***, CNTNAP5**, ITGBL1**, ZNF275**	*AFF1*, *ASH1L*, *C3orf21*, *CAP2*, *ELK3*, *CLSTN2*, *LOC442459*, *MYH13*, *MYLK4*, *NSMCE2*, *NUBPL*, *PHACTR1*, *RBM46*, *SAV1*, *SOX5*, *SPAG16*, *TBC1D1*, *TMEM232*, *TOP2B*, *WDR27*, *WWC3*, NLK***, TTL*** ACER3, ARL15, ATP8B4, C10orf11, C16orf62, C17orf104, C17orf67, COL14A1, CREB5, DCBLD1, DENND2A, DLC1, DOCK4, EFR3A, FAM155A, FAM188A, FAM73A, FKBP5, FYCO1, GAL3ST2, GALNTL6, GULP1, HPS3, IGF1R, IL31RA, KIAA0564, KLHL29, LRRC16A, MOXD1, MYO1E, NMBR, NXN, PDE4DIP, PPFIA2, PSMD14, RECK, RGL1, SCHIP1, SIK3, SLC2A13, SNX29, SPSB1, ST7, TEAD1, TNFRSF1B, UST, WWTR1, YWHAQ, ITGBL1**, CNTNAP5**, ZNF275**
**Pathways of DM genes with PGR binding sites in E2+P4 treated NUP (P<0.05)**	**Pathways of DM genes with PGR binding sites in E2+P4 treated Endo I (P<0.05)**	**Pathways of DM genes with PGR binding sites in E2+P4 treated Endo IV (P<0.05)**
Cellular component morphogenesis; cell morphogenesis; cell part morphogenesis;single-organism organelle organization; cytoskeleton organization; endosome organization; neuron development; cell morphogenesis involved in differentiation; cell projection morphogenesis; negative regulation of developmental growth; neuron projection development; regulation of axon extension; regulation of extent of cell growth	Tissue morphogenesis; cellular response to transforming growth factor beta stimulus; organ morphogenesis; morphogenesis of an epithelium; metanephros development; extracellular matrix organization; animal organ development; system development; cell morphogenesis involved in differentiation; response to growth factor; epithelium development; cell development; urogenital system development; neuron projection development; cell morphogenesis; neuron development; anatomical structure formation involved in morphogenesis; cellular component morphogenesis; cellular component morphogenesis; kidney development; cell surface receptor signaling pathway; renal system development; cellular response to organic substance; regulation of cellular response to transforming growth factor beta stimulus; negative regulation of transmembrane receptor protein serine/threonine kinase signaling pathway; gland development; odontogenesis; embryonic morphogenesis; regulation of transmembrane receptor protein serine/threonine kinase signaling pathway; regulation of cell projection organization; neuron projection guidance; signal transduction; tube morphogenesis; mammary gland development; regulation of signal transduction; telencephalon cell migration; circulatory system development; negative regulation of cellular component movement; forebrain cell migration; negative regulation of cellular response to transforming growth factor beta stimulus; forebrain generation of neurons; regulation of neurogenesis; brain development; tangential migration from the subventricular zone to the olfactory bulb; regulation of cell development	Negative regulation of Wnt signaling pathway; intracellular signal transduction; single-organism organelle organization

* Common in NUP and Endo I

** Common in Endo I and Endo IV

*** Common in NUP, Endo I and Endo IV; ***Bold Italics***, common in NUP and Endo IV

## Discussion

### Unique steroid hormone effects on normal endometrial stromal fibroblasts

The eSF is the most abundant steroid hormone-responsive cell in endometrium and is a master regulator of tissue function and pregnancy success, and thus how the steroid hormones E_2_ and P_4_ regulate the epigenome and transcriptional machinery in this cell type in a timeline similar to *in vivo* exposure is of high priority in understanding normal and abnormal eSF function in women. Effects of E_2_ and P_4_ alone on the hormone-epigenome interplay has largely been studied in breast cancer cell lines, providing key insights into hormone receptor topology, epigenetic genomic alterations, transcriptional regulation, and chromatin dynamics [28,29]. As these complex interactions are cell- and tissue-specific, extrapolating their properties to normal endometrium is limited, although a few studies have investigated the effects of E_2_ plus progestins, such as medroxyprogesterone acetate, in the presence of cAMP on eSF for 72 hrs [10] or longer with or without glucose in the culture medium on chromatin accessibility or histone marks [9,11]. These studies show altered chromatin accessibility in eSF decidualized with E_2_+MPA+cAMP [11] and provide significant insights into PGR binding across the genome and association with open chromatin [10].

The current study investigated the effects of estradiol, progesterone (individually) and their combination (without cAMP or other progestins) on the DNA methylome and transcriptome and their interplay in normal eSF at 14 days, mimicking *in vivo* exposure times. Moreover, we compared eSF from the inflammatory disorder, endometriosis, in the setting of lesser and more advanced stage disease to the normal eSF. The data herein revealed, for the first time, that E_2_ and P_4_ individually and together promote unique patterns and profiles in the normal DNA methylome of this cell type. E_2_ alone elicited broad changes, blunted by P_4_, and mostly result in open chromatin by inducing more loss of methylation and increased H3K27ac histone mark. Progesterone alone had a limited effect on the DNA methylome, and unlike E_2,_ elicited loss and gain of methylation equally. E_2_+P_4_ affected the epigenome less robustly than E_2_ alone, but showed more loss than gain of methylation. In support of our observation Vrljicak *et*. *al*. using transposase accessible chromatin followed by sequencing (ATAC-Seq) found altered chromatin accessibility with more open than closed chromatin loci after 4days treatment with MPA and cAMP [11]. These data suggest that E_2_ and P_4_ interact differently with the epigenome when combined than when individually administered suggestive of different mechanisms involved in the response of eSF to E_2_ and to P_4_ individually and in combination. Hormone-specific patterns and profiles were abnormal in both disease stages with more severe abnormalities associated with stage IV disease. The range of differences in individual loci with differential methylation and the number of enriched clusters and gene functions induced by each hormonal treatment in disease versus normal suggest inherent differences in disease and disease stages. However, in disease, as in normal, E_2_ induced more extensive alteration than E_2_+P_4_ followed by P_4_. Despite these differences, hormone-induced changes overall mainly involved CpG sites at the 3’end, intergenic regions, and enhancers, limited involvement of 5’end and 1^st^ exons and rarely involved CpG sites in close proximity to transcription start sites (TSS200) or CpG islands. Notably, E_2_ treatment of MCF-7 breast cancer cells also demonstrated minimal binding of ER to proximal promoter regions (up to 5kb) [30], despite their containing the majority of known EREs.

Whereas CpG sites in CpG islands (CGI) were minimally affected, CGI shores and shelves were more involved, regardless of methylation loss or gain or the type of hormone treatment, indicating a specific genome landscape interaction of hormones in this endometrial cell type. Whether the lack of involvement of CGIs reflects regulation of genes whose functions are not regulated by direct or indirect hormone-targeted mechanisms, or whether hormone response elements are not affected in CGIs is yet to be determined. In breast cancer cell lines gene expression [31] and DNA methylation profiles [32] as well as DNA methylation at several candidate genes at their CGIs [33] depend on their ER and PR status. This observation further highlights findings herein that the majority of differentially methylated loci in eSF_normal_ are located in the intergenic regions, 3’UTRs and enhancers, and do not involve regions in close proximity to TSS, 5’UTR and 1^st^ exons, where most CpG islands are located.

### Epigenetic signatures in endometriosis: Hormone response, disease subtypes, pre-existing abnormalities

Women with endometriosis have high prevalence of infertility with otherwise unknown etiologies and lower implantation, clinical pregnancy and live birth rates compared to those without disease [34]. Studies in humans [35] and animal models [36] suggest compromised implantation attributed, in part, to an abnormal response to P_4_ and the inflammatory milieu of the endometrium. The current study confirmed abnormal P_4_-regulated decidualization marker expression in eSF_endo_, largely attributed to “P_4_-resistance”, although P_4_ did have effects across the eSF_endo_ genome and PGR targets. However, eSF_endo_ additionally had different responses to E_2_ compared to eSF_normal_, which likely also contributes to abnormal endometrial function in women with disease (as described below). Of note, aberrant lack of ERα down-regulation at the time of implantation in endometrium of endometriosis women is considered key in implantation failure in women with disease [37]. However, endometrial-based infertility and effects on pregnancy outcomes are controversial, as large studies on IVF/ICSI outcomes in women with endometriosis and ovarian endometriomas revealed no differences in pregnancy rates [38,39] or a significant difference in endometrial receptivity array test in women with endometriosis versus controls [40]. How the aberrancies observed herein in P_4_-, or E_2_-induced epigenetic signatures are linked with implantation outcomes in women with endometriosis warrants further investigation.

Whether stage I and stage IV endometriosis are distinct disease sub-types has been the subject of debate. That eSF_stage-I_ differ greatly from eSF_stage-IV_ in hormone response supports distinct disease subtypes. Also, disparities in the DNA methylomes of eSF_stage-I_ and eSF_stage-IV_ *before* hormonal treatments further support distinct disease subtypes. The latter observation underscores pre-existing abnormalities in the eSF epigenome in the setting of endometriosis, and the data showed eSF_stage-IV_ with more extensive pre-existing differences affecting its responses, compared to eSF_stage-I_.

This is consistent with previous findings that the endometrial bulk tissue transcriptome differs between the two stages [18,26] and several endometriosis genome-wide analysis studies suggestive of a stronger genetically driven component for stage IV than stage I disease [41]. Of note, clinically, women with stage IV versus stage I endometriosis have significantly lower implantation rates (13.7% vs. 28.3%, respectively), pregnancy rates (22.6% vs. 40.0%, respectively) [42], and lower IVF pregnancy rates (13.84% vs. 21.12% respectively) [43]- believed due to endometrial abnormalities that reflect distinct subtypes of disease. Mapping hormone-genome interactions of these subtypes holds promise for innovative, targeted therapies to modify pre-existing and stage-specific abnormalities in endometrium of women with endometriosis and optimize endometrial receptivity for implantation and pregnancy success of women with endometriosis.

Dyson *et*. *al*. [44] have observed aberrant DNA methylation in the stromal fibroblast isolated from the endometriotic ectopic lesion. It remains to be determined whether and to what extent the ectopic lesion aberrances stem from the eutopic endometrial stromal fibroblasts.

Recently, Maekawa *et*. *al*. assessed the genome-wide methylome changes during decidualization and in contrast to our data reported no changes in the DNA methylome [45]. DNA methylation distribution follows a bimodal distribution with the majority of CpG sites either hypomethylated or hypermethylated [46], as also reported in their study as well as in the current study and as we have previously observed in normal and endometriosis endometrium [14,15] Furthermore, we have also observed that the majority of CpG sites remain unchanged in decidualized versus non-decidualized eSF. The differences in our observation could be due to different analyses methods, where Maekawa *et*. *al* limited the definition of differential methylation to >Δβ of 0.3, which would not detect smaller changes. In our analyses we considered smaller changes in the DNA methylome but with the stringency that they were observed in at least 75% of each sample group. Another reason could be due to differences in the samples, where we used normal controls while patients with myoma or cervical cancer were used in their study, or it is likely that E_2_+MPA used in that study affects the epigenome differently than E_2_+P_4_ in our study.

### Progesterone “resistance”

Pursuing bulk tissue transcriptomic analysis, we first described “P_4_ resistance” in endometrium from women with endometriosis [8,35] a phenomenon also observed by others [47–49]. In samples obtained in the implantation window and timed to the LH surge, there was evidence for impaired expression of key epithelial and stromal fibroblast markers of embryo receptivity and decidualization, respectively [35]. Analysis of endometrium across the menstrual cycle from women with severe disease strikingly revealed persistent E_2_-regulated genes in the early secretory phase, consistent with impaired P_4_ action [8]. Moreover, these data were substantiated in a larger cohort [26], that also revealed a marked pro-inflammatory phenotype within the endometrium of women with disease. Inflammation can cause epigenetic changes in endometrium as demonstrated in an animal model of the disease [50]. We and others found P_4_-resistance in eSF [19,51]. Notably, inflammatory cytokines (e.g., IL-1β and TNFα) epigenetically silence the eSF PR, promoting P_4_-resistance with diminished expression of decidualization markers IGFBP1 and prolactin [50,52] and enhanced secretion of matrix metalloproteinases, which are normally suppressed in eSF by P_4_ [53]. Epigenetic mechanisms underlying P_4_-resistance in endometriosis have mostly focused on the disease itself (as opposed to the eutopic endometrium studied herein) which exhibits P_4_ and progestin resistance for pain relief initially or acquired over time [54].

We suggest that the nomenclature of “P_4_ resistance” be re-evaluated, since the data herein show eSF_endo_ respond to P_4_ with regard to epigenetic marks, PGR target sites, and gene expression, albeit differently from eSF_normal_, although they do not fully decidualize. Notably, endometrium of women with disease does not retain a proliferative phenotype throughout the cycle [6,8], although there is compromised implantation in women with disease [35,52]. Importantly, “P_4_ -resistance” was observed in endometrium of non-pregnant women who previously had severe pre-eclampsia, and this was also found in the decidua at delivery of women with this disorder [55], underscoring the need to understand this process for normal pregnancy. Thus, P_4_ signaling in the endometrium and aberrancies in decidualization therein *in vivo* are likely influenced by other cell types in the tissue, including the inflammatory status of the individual and warrant further investigation.

### Abnormal response to E_2_

Herein, for the first time the observations have been made that in addition to aberrant eSF_endo_ P_4_ response, eSF from women with endometriosis show vastly aberrant responses to E_2._ Specifically, E_2_-induced eSF DNA methylation changes blunted in stage IV disease and were more extensive in stage I. Loci with strong associations of DNA methylation and gene expression had distinct enrichment in gene functions in stage I and stage IV, including ion channels, ATP and nucleotide binding in stage I and plasma membrane and signaling in stage IV, suggesting functional impairment of eSF from women with versus without endometriosis. Moreover, they underscore that not only is the eSF response to P_4_ abnormal in women with disease, but also *their response to E*_*2*_ *is abnormal*. The latter has received little attention in the endometriosis literature, which is surprising, as the disorder is estrogen-dependent [12,21]. As eSF normally require E_2_ priming prior to the full decidualization P_4_ response, it is not unanticipated that with abnormal E_2_ signaling in eSF, P_4_ signaling would also be disrupted. Aromatase, essential for E_2_ production, as well as E_2_ levels are highly expressed in endometriotic tissue [56–59] with an increased COX2 expression in turn resulting in increased E_2_ production in a positive feedback loop in ectopic and eutopic tissue of endometriosis patients [57]. Whether the aberrant response to E_2_ observed herein could be affected by these aberrancies in E_2_ production in endometriosis remains to be determined.

### Potential mechanisms of E_2_-epigenome interactions

The binding of some hormone NRs commonly occurs at accessible regions of the chromatin before hormone induction [60] or their recruitment occurs almost equally at the nucleosome-occupied and nucleosome-free states before hormone induction [61]. E_2_ (biological active estrogen) enters cells, binds to subtypes of ERα and β that have high affinity for E_2_ and are encoded by different genes [62]. While both ER subtypes are expressed in human endometrium, ERα is the primary mediator of E_2_ action in this tissue [63]. ERα recruitment is complex involving multiple mechanisms depending on cell type and culture conditions [64]. ERα can bind to compact chromatin while there are abundant accessible regions before E_2_ induction that will further recruit ERα [65]. The DNA methylation and histone modification findings herein suggest that E_2_ can increase open chromatin. Chromatin accessibility can be induced by ERα binding, as these accessible clusters are found near estrogen-target genes [66]. While increased open chromatin was found in eSF_normal_ in response to E_2_, the opposite was found in eSF_endo_. This could be due to pre-existing abnormalities in disease affecting chromatin structure, a combination of transcriptional machinery preloaded across the genome, or, as found in disease, up to 50% of loci displaying pre-existing differences in epigenetic signatures influencing this response. Furthermore, the state of chromatin compaction may play an important role. About half of EREs are in regions of DNA with open chromatin prior to estrogen induction [67], but many ERα binding sites in open chromatin are associated with differentially expressed genes after estrogen induction. These data indicate that chromatin compaction can directly affect ERα recruitment and subsequently target gene transcription. Thus, pre-existing and distinct differential methylation observed in stage I and stage IV can potentially affect chromatin compaction in patients with endometriosis. This is currently under investigation in our laboratory.

ERα can be activated by phosphorylation by growth factors binding to tyrosine kinase receptors such as EGFR [68], which were dysregulated in the current study. Genes targeted by phosphorylated ERα are distinct from those targeted by estrogen-induced ERα activation [69]. Signaling through EGFR is a key pathway in eSF response to E_2_, and constitutive activation of EGFR in eSF from women with endometriosis has been reported [70]. Inhibition of EGFR in eSF from women with disease restores decidualization markers [71], underscoring the complexity of the interplay between E_2_ and P_4_ signaling in eSF in endometrium of women with endometriosis. The data overall support phosphorylation of ERα in eSF treated with E_2_ may contribute, in part, to differential DNA methylation signatures and gene expression profiles observed in E_2_ versus E_2_+P_4_ in women without and with disease, which remains to be proven experimentally. Interestingly, E_2_ and EGF can induce ERα recruitment at three classes of enhancers [72], bound only with EGF stimulation, only with E_2_ stimulation, or either. Herein, enrichment of enhancer involvement upon E_2_ stimulation and with E_2_+P_4_ was observed in normal eSF as well as eSF from women with both stages of disease, but with different effects on downstream target genes. We propose that even small differences in EGFR signaling pathways could greatly alter the eSF responses to hormones, as observed herein.

### Study strengths and limitations and future directions

In this study, effects of E_2_, P_4_ and their combination were elucidated on genome-wide DNA methylation marks of the endometrial stromal fibroblast, the predominant cell type in human endometrium essential for establishing and continuing pregnancy. The clinical phenotyping of truly normal controls and specific, well-phenotyped disease stages is a great strength of this study. Also, using the same cells for DNA methylation and gene expression analyses also added to the robustness of the data. Moreover, comparisons of the data herein with published gene expression and DNA methylation data in bulk tissue underscore signatures in the latter due to this predominant cell type. Single cell analysis of eSF from bulk tissue by FACS further underscores the signature of this cell type in overall bulk tissue analyses and opens the door for single cell RNA-Seq and DNA-me analyses in the future.

While use of an *in vitro* system can address whether/how steroid hormones directly affect the eSF epigenome, an *in vivo* model using freshly isolated, sorted endometrial cells would offer an opportunity to assess functionality of the ER and PR landscape in human eSF and other endometrial cell types. Primary epithelial cells were not viable in culture when treated with hormones and as such we did not expand the current study involving epithelial cells, but organoid systems may offer a tool for this investigation. Further analysis using chromatin immunoprecipitation followed by deep sequencing of ER and expanding on the PR binding sites by Mazur *et*.*al* [10], identification of ERE and PRE-specific to endometrium and the status important to pioneer and co-activators and in a larger sample size are required for detailed mapping of the steroid hormone landscapes and hormone-epigenome interplay in normal human endometrial cells and in disease. Transcriptome data from the same cells demonstrate extensive overlap with previously identified differentially expressed genes in whole tissue in the corresponding hormone milieu. We note that utilizing microarray instead of a more comprehensive transcriptome analysis such as RNA-Seq limited the number and the type of transcripts investigated herein. Furthermore, protein data will enable full assessment of epigenomic and transcriptomic effects of hormones in endometrial function normally and in women with endometriosis. An important limitation of this study is the small sample size in each group. We had used very strict criteria for sample selection, both in identifying normal samples without any gynecological and pelvic disease/disorder and for endometriosis to not have any other disease no matter how benign, such as uterine fibroids. A follow up study with a much larger sample size is required to confirm our observations.

Overall this study has elucidated the array of responses of eSF in health and disease in hormone milieu encountered in cycling women that can also serve for comparisons with actions of pharmaceutical steroids used clinically and potentially environmental estrogens that can compromise reproductive function. Moreover, the data reveal unique responses and pre-existing epigenetic abnormalities in women with endometriosis that can benefit endometrial-based diagnostic development and novel targeted therapies for endometrial dysfunction in women with this disorder.

## Materials and methods

### Ethics statement

This study was approved by the Committee on Human Research of the University of California, San Francisco (UCSF) (IRB# 10–02786). All samples were collected after written informed consent was obtained from all subjects.

### Samples

Eutopic (within the uterus) endometrial tissue samples were collected through the UCSF/NIH Human Endometrial Tissue/DNA Bank. Stringent inclusion criteria were applied as follows: I) for normal controls, samples were collected from oocyte donor volunteers with no uterine or pelvic pathology (NUP, normal controls); endometriosis samples were collected from stage I and stage IV endometriosis patients (**S18 Table**). Oocyte donor volunteers (controls) were extensively screened, had no gynecologic disorders, and donated endometrial samples six months post oocyte retrieval. Endometriosis patients (stage I and IV) were surgically confirmed and had no other gynecologic abnormalities. II) All samples were collected in the proliferative phase and matched for age, BMI, no smoking history (one exception), no contraceptive steroid use three months prior to sample collection, and endometrial stromal fibroblast (eSF) passage number. Menstrual cycle phase was determined by histological evaluation [73] as well as serum levels of E_2_ and P_4_. Disease stage was determined by ASRM criteria [74].

### Stromal cell isolation and hormone treatment

Primary eSF were isolated from endometrial biopsies by digestion with collagenase and size fractionation as described [75] and cultured as monolayers in stromal cell medium (SCM, [18,19,76]). To ensure the purity of stromal cells in culture, after digestion of endometrial sample biopsies, the digested tissue was size fractionated using a 40μM filter to separate epithelial glands, followed by stromal cells selective attachment and growth in stromal cell medium. The purity of primary eSF was monitored morphologically during the culture and the homogeneity was verified by immunocytochemical localization of vimentin for eSF, keratin for epithelial cells and actin for vascular cells [76] before further hormone experiments. Only pure primary eSF (with <0.1% other cells) were used for this study. After 24 hours serum starvation, eSF from normal women (n = 7, controls) and endometriosis women (n = 6 stage I, n = 9 stage IV) were treated with four different hormonal treatments of 10 nM E_2_, 1μM P_4_, 10 nM E_2_ +1μM P_4_, or vehicle (0.1% ethanol) control for 14 days [76]) after which conditioned media and cells were collected for further analysis. Decidualization was assessed in E_2_+P_4_ treated eSF from normal, stage I and stage IV disease (see below). As eSF from women without endometriosis mostly have a robust decidualization response to E_2_+P_4_, our controls were eSF that fully decidualized (n = 4) by the decidualization marker IGFBP1 by ELISA and morphologically. As rarely do eSF from endometriosis women decidualize *in vitro* in response to E_2_+P_4_, eSF from stage I (n = 4) and stage IV (n = 4) with non-detectable decidualization (the most common phenotype) by morphology and IGFBP1 marker by ELISA were used for further analysis.

### Decidualization assessment

Insulin-like growth factor binding protein-1 (IGFBP1), a P_4_-induced decidualization marker [77], was measured in media conditioned by 14 day E_2_+P_4_ treated cultures, by ELISA (Alpha Diagnostic International Inc., San Antonio, TX) as a marker for decidualization. Concentrations were measured in duplicate, averaged and normalized to cell number. eSF were assessed by microscopy for morphological changes corresponding to decidualization.

### DNA and RNA extraction

After treatments cells were harvested, pelleted and frozen at -80C for DNA and RNA extraction as previously described [14,15]. Genomic DNA was extracted using QIAGEN (QIAamp DNA Tissue Kit, QIAGEN, Germantown, MD) and RNA was extracted using the Macherey-Nagel NuceloSpin Tissue Kit with DNase treatment (Macherey-Nagel Inc., Bethlehem, PA) according to manufacturers’ recommendations and stored at -80C.

### DNA methylation

Genomic DNA was bisulfite converted at the University of Southern California (USC) Epigenome Center using the EZ-96 DNA Methylation Kit (Zymo Research, Irvine, CA), according to the manufacturer’s protocol, and as previously described [14,15]. Quality, completeness of bisulfite conversion and amount of bisulfite-converted DNA were assessed by a panel of MethyLight reactions [78]. All samples passed all quality controls (QCs) and were further assayed by the Illumina Infinium HumanMethylation450K DNA methylation platform (HM450) based on Illumina’s specifications. (All data files are submitted to GEO, under SuperSeries accession number GSE145702).

### Gene expression microarray analysis

RNA quality was assessed by Bioanalyzer 2100 (Agilent Technologies, Santa Clara, CA). RNA samples were prepared for microarray analysis according to Affymetrix (Affymetrix, Inc., Santa Clara, CA) specifications [15]. cDNA sample quality was assessed by Bioanalyzer, and samples passing QCs were hybridized to Affymetrix HU133 Plus 2.0 gene array, interrogating >38,500 genes at the UCSF Genomics Core.

### DNA methylation data analysis

HM450 interrogates 485,577 methylation targets across the genome. The ratio of methylated signal over total fluorescent signal was used to calculate β values, ranging from 0 (no) to 1 (complete) methylation. 850 control bead types were used as positive and negative controls and to calculate a detection P value to assess DNA methylation measurement quality for each probe of each sample [79]. The passing threshold of P value was set at P<0.05, and probes with P>0.05 were indicated as “missing” (no statistically significant differences from background). Probe dropout-rates (percent probes with missing values versus total number of platform probes) were calculated to exclude samples with dropout-rates >1%. All 12 samples passed these criteria. Probes with a “missing” value in >1 sample were removed. Differential DNA methylation in each hormone treatment in each group (control, stage I, stage IV) was identified compared to non-treated cells (vehicle control) in the same group. For each probe, β values of hormone treatment in an individual sample were compared to its corresponding vehicle treated β value (Δβ). Changes in β values (Δβ) < 10% were not considered as differentially methylated for each sample. Median or average changes between hormone treatment vs vehicle control were not used, to obviate limited numbers of strong signals affecting selecting differentially methylated loci. Instead, probes were considered differentially methylated if they exhibited >10% change in β value (Δβ >10%) in hormone treatment versus vehicle *and* in at least 3 of 4 samples within each group, *and* with the same direction of methylation change (gain or loss). To assess if the aberrant signatures observed in hormone induced changes in disease compared to normal were due to pre-existing aberrations in the non-treated cells, we compared the methylation signatures of the non-treated (vehicle) eSF in each disease stage to non-treated (vehicle) eSF in normal for each aberrantly methylated locus in response to each hormone in disease. Pre-existing differential methylation, loci differentially methylated in normal vs each stage of disease in untreated cells (vehicle) were determined. The percentage of the differences observed in hormonal treatment of disease were assessed. Association of CpG islands with enhancers, distribution across the genome, association with CGIs and CGI shores and shelves were extracted from Illumina Infinium HumanMethylation450K manifest.

### Gene expression analysis

The raw.CEL gene expression data files were RMA normalized using GeneSpring (GX13.1 version, Agilent Technologies). Loci were considered differentially expressed with Benjamini-Hochberg corrected ANOVA p<0.05 and fold change (FC)≥1.5 in each comparison.

### DNA methylation association with changes in gene expression

Differentially methylated loci and normalized gene expression were imported into R, and corresponding probes from each platform were matched using the transcript identifier. Every DNA methylation probe for a given locus identifier was compared to all corresponding transcripts of that locus using the non-parametric Spearman’s rank-order correlation method, as bivariate normality could not be assumed (DNA methylation data are not normally distributed). Spearman's rank correlation coefficients (ρ) on gene expression and DNA methylation were computed for each probe, along with a p value testing against the null hypothesis that ρ equals zero.

### Pathways, biofunctions and genomic distribution analyses

Ingenuity Pathway Analysis (IPA, QIAGEN) software was used to determine pathways, upstream regulators and biofunctions of differentially expressed genes, as described [14,15,26]. Pathways with Z-scores ≥|2| were considered significantly enriched. For differentially methylated loci, DAVID and KEGG databases were used to identify functional classification, functional enrichment and pathways. For pathway selection an enrichment score ≥|2| with a Benjamini-Hochberg corrected p<0.05 was considered. For genomic distribution, element enrichment analyses, the null hypothesis that the observed proportions in two groups are the same, a test of proportions was performed in R using the prop.test() function.

### Association of *in vitro* and *in vivo* differential gene expression

eSF treatment with E_2_ and E_2_+P_4_ used herein, mimicked *in vivo* proliferative endometrium (PE, E_2_-dominant phase) and mid-secretory endometrium (MSE (E_2_ and P_4_- dominant phase). To assess commonalities, the current data were compared to previously published [26] whole endometrial gene expression in PE and MSE in normal versus disease. E_2_-treated differential expression in eSF from stage I versus control and stage IV versus control were compared to endometriosis (all stages) versus control in bulk tissue PE, and E_2_+P_4_- treated compared to MSE. FACS-sorted eSF gene expression data [20] in disease versus control were also compared. As FACS-sorted eSF_endo_ and eSF_normal_ included a mixture of phases and endometriosis stages, gene expression signatures of FACS-sorted eSF were compared to stage I, stage IV eSF treated with E_2_ and E_2_+P_4_.

### ChIP-Seq for Histone H3K27me3 and H3K27ac in response to E_2_

We found that E_2_ affected the methylome more robustly than P_4_ or E_2_+P_4_ and that, unexpectedly, along with aberrant P_4_ response in disease, E_2_ response was also aberrant in both stages of disease. Therefore, we sought to investigate further the effect of E_2_ on two repressive and open chromatin histone marks, H3K27me3 and H3K27ac. eSF cells from two independent control participant were isolated from endometrial biopsies by digestion followed by size fractionation, and primary eSF were cultured in SCM and purity of cultured eSF was assessed as described above. eSF was passaged with trypsin and 1x10^5^ cell/well were seeded. Confluent cultures were serum starved for 24hrs and treated with E_2_ or vehicle for 14 days. Cells were cross-linked by a final concentration of 1% formaldehyde and terminated after 10 minutes by 0.125 M final concentration glycine. Chromatin was extracted using Chromatin Extraction Kit according to manufacturer’s recommendation (ab117152, Abcam, Cambridge, UK) sonicated by Diagenode Bioruptor and the size of sheared chromatin was visualized on agarose gel (100-600bp). Chromatin Immunoprecipitation was done using Abcam ChIP Kit (ab117138, Abcam, Cambridge, UK) with antibodies for H3K27me3 (ab6002, Abcam, Cambridge, UK) or H3K27ac (ab4729, Abcam, Cambridge, UK). Input control and immunoprecipitated DNA were paired-end sequenced using Illumina NextSeq 500 after library preparation according to the manufacturer’s instructions. Data were analyzed by removing adapter sequences, then aligned to reference human genome. Peaks called using Macs2 *callpeaks* and were selected with q-value <0.05. Differential peaks were identified using Macs2 *bdgdiff* and log likelihood ratio >3.

## Supporting information

S1 Fig IGFBP1 ELISA assay of normal, stage I and IV eSFs used in the study. (PDF)

S2 Fig Genomic distribution of differentially methylated CpG sites in each hormonal treatment, by gain or loss of methylation, in normal (NUP), stage I (Endo I) and stage IV (Endo IV) eSF. (PDF)

S3 Fig Heat-map of pre-existing aberrancies prior to hormone treatments and percentage of contribution to each hormone induced methylation in stage I and stage IV eSF. (PDF)

S1 Table Differentially methylated CpG sites in response to E_2_, P_4_, E_2_+P_4_ (vs. vehicle) in Normal eSF, based on loss and gain of methylation. (XLSX)

S2 Table Distribution of differentially methylated loci (in %) based on their location across the genome and in comparison to those interrogated within the platform in normal (NUP), stage I (Endo I) and stage IV (Endo IV). (XLSX)

S3 Table Distribution of differentially methylated loci (in %) based on their association with CpG islands (CGI), and CGI north/south shelf, CGI north/south shore and not associated with CGIs (open sea), and in comparison to the interrogated CGI in the platform in normal (NUP), stage I (Endo I) and stage IV (Endo IV). (XLSX)

S4 Table Differentially expressed genes in hormone treatments (E_2_, P_4_, E_2_+P_4_) vs. vehicle in normal eSF (NUP). (XLSX)

S5 Table Enriched pathways and the associated genes and upstream regulators of differentially expressed gene in response to hormones (E_2_, P_4_, E_2_+P_4_) vs. vehicle, in normal (NUP), stage I (Endo I), and stage IV (Endo IV) disease. (XLSX)

S6 Table Differentially methylated CpG sites in response to E_2_, P_4_, E_2_+P_4_ (vs. vehicle) in endometriosis stage I eSF, based on loss and gain of methylation. (XLSX)

S7 Table Differentially methylated CpG sites in response to E_2_, P_4_, E_2_+P_4_ (vs. vehicle) in endometriosis stage IV eSF, based on loss and gain of methylation. (XLSX)

S8 Table Differentially expressed genes in hormone treatments (E_2_, P_4_, E_2_+P_4_) vs. vehicle in stage I disease (Endo I). (XLSX)

S9 Table Differentially expressed genes in hormone treatments (E_2_, P_4_, E_2_+P_4_) vs. vehicle in eSF from stage IV disease (Endo IV). (XLSX)

S10 Table Unique and common up- and down-regulated genes in each hormone treatment and in normal (NUP), stage I (Endo I) and stage IV Endo (IV). (XLSX)

S11 Table Association of differentially methylated (DM) loci with differentially expressed (DE) genes in normal eSF (NUP) for all hormone treatments (E_2_, P_4_, E_2_+P_4_) vs. vehicle, based on positive or negative association (positive/negative rho) and gain and loss of methylation (gain, loss). (XLSX)

S12 Table Association of differentially methylated (DM) loci with differentially expressed (DE) genes in stage I eSF (Endo I) for all hormone treatments (E_2_, P_4_, E_2_+P_4_) vs. vehicle, based on positive or negative association (positive/negative rho) and gain and loss of methylation (gain/loss). (XLSX)

S13 Table Association of differentially methylated (DM) loci with differentially expressed (DE) genes in stage IV eSF (Endo IV) for all hormone treatments (E_2_, P_4_, E_2_+P_4_) vs. vehicle, based on positive or negative association (positive/negative rho) and gain and loss of methylation (gain/loss). (XLSX)

S14 Table Differentially methylated (DM) loci associated with differentially expressed (DE) assessed by Spearman rho, with either positive association (pos) or negative association (neg). Common/unique genes are shown in columns and in Venn diagrams, in normal (NUP), stage I (Endo I), and stage IV (Endo IV).
(XLSX)

S15 Table Overlap of *in vivo* with *in vitro* genes and biofunctions from hormonal treatments in culture and endometrial tissue cycle phases in endometriosis stage I or stage IV versus normal. (XLSX)

S16 Table Genes commonly differentially expressed in eSF from disease vs. normal, treated or untreated in vitro with hormones and in eSF FACS-isolated in disease vs. normal. (XLSX)

S17 Table Peaks and GO biofunctions enriched in histone marks H3K27me3 and H3K27ac in normal eSF induced by E_2_ versus vehicle. (XLSX)

S18 Table Sample information. (XLSX)

S1 Data Differentially methylated loci and the associated pathways/biofunctions that are affected in hormonal treatments of eSF_normal,_ eSF_stage I_ and eSF_stage IV_ with known roles/importance in normal endometrial function and dysfunction in endometriosis. (PDF)

## References

[1] Gargett CE, Chan RWS, Schwab KE. Endometrial stem cells. Curr Opin Obstet Gynecol. 2007;19: 377–83. 10.1097/GCO.0b013e328235a5c6 17625422

[2] Macklon NS, Brosens JJ. The Human Endometrium as a Sensor of Embryo Quality1. Biol Reprod. 2014;91 10.1095/biolreprod.114.122846 25187529

[3] Vicent GP, Nacht AS, Font-mateu J, Castellano G, Gaveglia L, Beato M. Four enzymes cooperate to displace histone H1 during the first minute of hormonal gene activation. 2011; 845–862. 10.1101/gad.621811 21447625 PMC3078709

[4] Heldring N, Isaacs GD, Diehl AG, Sun M, Cheung E, Ranish JA, et al Multiple Sequence-Specific DNA-Binding Proteins Mediate Estrogen Receptor Signaling through a Tethering Pathway. Mol Endocrinol. 2011;25: 564–574. 10.1210/me.2010-0425 21330404 PMC3063082

[5] Kittler R, Zhou J, Hua S, Ma L, Liu Y, Pendleton E, et al A Comprehensive Nuclear Receptor Network for Breast Cancer Cells. Cell Rep. 2013;3: 538–551. 10.1016/j.celrep.2013.01.004 23375374

[6] Al-Sabbagh M, Lam EW-F, Brosens JJ. Mechanisms of endometrial progesterone resistance. Mol Cell Endocrinol. 2012;358: 208–215. 10.1016/j.mce.2011.10.035 22085558

[7] Piestrzeniewicz-Ulanska D, Brys M, Semczuk A, Jakowicki JA, Krajewska WM. Expression of TGF-beta type I and II receptors in normal and cancerous human endometrium. Cancer Lett. 2002;186: 231–9. Available: http://www.ncbi.nlm.nih.gov/pubmed/12213293 10.1016/s0304-3835(02)00351-8 12213293

[8] Burney RO, Talbi S, Hamilton AE, Vo KC, Nyegaard M, Nezhat CR, et al Gene Expression Analysis of Endometrium Reveals Progesterone Resistance and Candidate Susceptibility Genes in Women with Endometriosis. Endocrinology. 2007;148: 3814–3826. 10.1210/en.2006-1692 17510236

[9] Tamura I, Ohkawa Y, Sato T, Suyama M, Jozaki K, Okada M, et al Genome-wide analysis of histone modifications in human endometrial stromal cells. Mol Endocrinol. 2014;28: 1656–1669. 10.1210/me.2014-1117 25073104 PMC5414786

[10] Mazur EC, Vasquez YM, Li X, Kommagani R, Jiang L, Chen R, et al Progesterone Receptor Transcriptome and Cistrome in Decidualized Human Endometrial Stromal Cells. Endocrinology. 2015;156: 2239–2253. 10.1210/en.2014-1566 25781565 PMC4430623

[11] Vrljicak P, Lucas ES, Lansdowne L, Lucciola R, Muter J, Dyer NP, et al Analysis of chromatin accessibility in decidualizing human endometrial stromal cells. FASEB J. 2018;32: 2467–2477. 10.1096/fj.201701098R 29259032 PMC6040682

[12] Giudice LC. Endometriosis. N Engl J Med. 2010;362: 2389–2398. 10.1056/NEJMcp1000274 20573927 PMC3108065

[13] Bulun SE, Cheng Y-H, Pavone ME, Xue Q, Attar E, Trukhacheva E, et al Estrogen receptor-beta, estrogen receptor-alpha, and progesterone resistance in endometriosis. Semin Reprod Med. 2010;28: 36–43. 10.1055/s-0029-1242991 20104427 PMC3073375

[14] Houshdaran S, Nezhat CR, Vo KC, Zelenko Z, Irwin JC, Giudice LC. Aberrant Endometrial DNA Methylome and Associated Gene Expression in Women with Endometriosis. Biol Reprod. 2016;95: 93–93. 10.1095/biolreprod.116.140434 27535958 PMC5178151

[15] Houshdaran S, Zelenko Z, Irwin JC, Giudice LC. Human endometrial DNA methylome is cycle- dependent and is associated with gene expression regulation. Mol Endocrinol. 2014;28 10.1210/me.2013-1340 24877562 PMC4075160

[16] Saare M, Modhukur V, Suhorutshenko M, Rajashekar B, Rekker K, Sõritsa D, et al The influence of menstrual cycle and endometriosis on endometrial methylome. Clin Epigenetics. 2016;8: 2 10.1186/s13148-015-0168-z 26759613 PMC4710036

[17] Vannuccini S, Clifton VL, Fraser IS, Taylor HS, Critchley H, Giudice LC, et al Infertility and reproductive disorders: impact of hormonal and inflammatory mechanisms on pregnancy outcome. Hum Reprod Update. 2016;22: 104–115. 10.1093/humupd/dmv044 26395640 PMC7289323

[18] Aghajanova L, Horcajadas JA, Weeks JL, Esteban FJ, Nezhat CN, Conti M, et al The Protein Kinase A Pathway-Regulated Transcriptome of Endometrial Stromal Fibroblasts Reveals Compromised Differentiation and Persistent Proliferative Potential in Endometriosis. Endocrinology. 2010;151: 1341–1355. 10.1210/en.2009-0923 20068008 PMC2840687

[19] Aghajanova L, Tatsumi K, Horcajadas JA, Zamah AM, Esteban FJ, Herndon CN, et al Unique Transcriptome, Pathways, and Networks in the Human Endometrial Fibroblast Response to Progesterone in Endometriosis. Biol Reprod. 2011;84: 801–815. 10.1095/biolreprod.110.086181 20864642 PMC3062042

[20] Barragan F, Irwin JC, Balayan S, Erikson DW, Chen JC, Houshdaran S, et al Human Endometrial Fibroblasts Derived from Mesenchymal Progenitors Inherit Progesterone Resistance and Acquire an Inflammatory Phenotype in the Endometrial Niche in Endometriosis. Biol Reprod. 2016;94 10.1095/biolreprod.115.136010 27075616 PMC4939744

[21] Bulun SE. Endometriosis. N Engl J Med. 2009;360: 268–279. 10.1056/NEJMra0804690 19144942

[22] Welboren W-J, van Driel MA, Janssen-Megens EM, van Heeringen SJ, Sweep FC, Span PN, et al ChIP-Seq of ERα and RNA polymerase II defines genes differentially responding to ligands. EMBO J. 2009;28: 1418–1428. 10.1038/emboj.2009.88 19339991 PMC2688537

[23] Hah N, Kraus WL. Hormone-regulated transcriptomes: Lessons learned from estrogen signaling pathways in breast cancer cells. Mol Cell Endocrinol. 2014;382: 652–664. 10.1016/j.mce.2013.06.021 23810978 PMC3844033

[24] Kim JJ, Kurita T, Bulun SE. Progesterone Action in Endometrial Cancer, Endometriosis, Uterine Fibroids, and Breast Cancer. Endocr Rev. 2013;34: 130–162. 10.1210/er.2012-1043 23303565 PMC3565104

[25] Aghajanova L, Giudice LC. Molecular evidence for differences in endometrium in severe versus mild endometriosis. Reprod Sci. 2011;18: 229–251. 10.1177/1933719110386241 21063030 PMC3118406

[26] Tamaresis JS, Irwin JC, Goldfien GA, Rabban JT, Burney RO, Nezhat C, et al Molecular classification of endometriosis and disease stage using high-dimensional genomic data. Endocrinology. 2014;155: 4986–99. 10.1210/en.2014-1490 25243856 PMC4239429

[27] Vasquez YM, Mazur EC, Li X, Kommagani R, Jiang L, Chen R, et al FOXO1 is Required for Binding of PR on IRF4, Novel Transcriptional Regulator of Endometrial Stromal Decidualization. Mol Endocrinol. 2015;29: 421–433. 10.1210/me.2014-1292 25584414 PMC4347287

[28] Jadhav RR, Ye Z, Huang R-L, Liu J, Hsu P-Y, Huang Y-W, et al Genome-wide DNA methylation analysis reveals estrogen-mediated epigenetic repression of metallothionein-1 gene cluster in breast cancer. Clin Epigenetics. 2015;7: 13 10.1186/s13148-015-0045-9 25763113 PMC4355986

[29] Liu MH, Cheung E. Estrogen receptor-mediated long-range chromatin interactions and transcription in breast cancer. Mol Cell Endocrinol. 2014;382: 624–632. 10.1016/j.mce.2013.09.019 24071518

[30] Carroll JS, Meyer CA, Song J, Li W, Geistlinger TR, Eeckhoute J, et al Genome-wide analysis of estrogen receptor binding sites. Nat Genet. 2006;38: 1289–1297. 10.1038/ng1901 17013392

[31] Creighton CJ, Kent Osborne C, van de Vijver MJ, Foekens JA, Klijn JG, Horlings HM, et al Molecular profiles of progesterone receptor loss in human breast tumors. Breast Cancer Res Treat. 2009;114: 287–299. 10.1007/s10549-008-0017-2 18425577 PMC2635926

[32] Li L, Lee K-M, Han W, Choi J-Y, Lee J-Y, Kang GH, et al Estrogen and progesterone receptor status affect genome-wide DNA methylation profile in breast cancer. Hum Mol Genet. 2010;19: 4273–4277. 10.1093/hmg/ddq351 20724461

[33] Widschwendter M, Siegmund KD, Müller HM, Fiegl H, Marth C, Müller-Holzner E, et al Association of breast cancer DNA methylation profiles with hormone receptor status and response to tamoxifen. Cancer Res. 2004;64: 3807–13. 10.1158/0008-5472.CAN-03-3852 15172987

[34] Lessey BA, Kim JJ. Endometrial receptivity in the eutopic endometrium of women with endometriosis: it is affected, and let me show you why. Fertil Steril. 2017;108: 19–27. 10.1016/j.fertnstert.2017.05.031 28602477 PMC5629018

[35] Kao LC, Germeyer A, Tulac S, Lobo S, Yang JP, Taylor RN, et al Expression Profiling of Endometrium from Women with Endometriosis Reveals Candidate Genes for Disease-Based Implantation Failure and Infertility. Endocrinology. 2003;144: 2870–2881. 10.1210/en.2003-0043 12810542

[36] Fazleabas A. Progesterone Resistance in a Baboon Model of Endometriosis. Semin Reprod Med. 2010;28: 075–080. 10.1055/s-0029-1242997 20104431

[37] Lessey BA, Palomino WA, Apparao K, Young SL, Lininger RA. Estrogen receptor-alpha (ER-alpha) and defects in uterine receptivity in women. Reprod Biol Endocrinol. 2006;4: S9 10.1186/1477-7827-4-S1-S9 17118173 PMC1679803

[38] Hamdan M, Dunselman G, Li TC, Cheong Y. The impact of endometrioma on IVF/ICSI outcomes: A systematic review and meta-analysis. Hum Reprod Update. 2015;21: 809–825. 10.1093/humupd/dmv035 26168799

[39] Singh N, Lata K, Naha M, Malhotra N, Tiwari A, Vanamail P. Effect of endometriosis on implantation rates when compared to tubal factor in fresh non donor in vitro fertilization cycles. J Hum Reprod Sci. 2014;7: 143–147. 10.4103/0974-1208.138874 25191029 PMC4150142

[40] Garcia-Velasco JA, Fassbender A, Ruiz-Alonso M, Blesa D, D’Hooghe T, Simon C. Is endometrial receptivity transcriptomics affected in women with endometriosis? A pilot study. Reprod Biomed Online. 2015;31: 647–654. 10.1016/j.rbmo.2015.07.014 26385059

[41] Nilufer Rahmioglu, Karina Banasik, Paraskevi Christofidou, Rebecca Danning, Genevieve Galarneau, Ayush Giri, Stuart MacGregor, Sally Mortlock, Yadav Sapkota, Schork J Andrew Sobalska-Kwapis Marta, Lilja Stefansdottir, Constance Turman, Outi Uimari, Adach ZTK. Large-scale genome-wide association meta-analysis of endometriosis reveals 13 novel loci and genetically-associated comorbidity with other pain conditions. biRxiv. 2018.

[42] Kuivasaari P, Hippeläinen M, Anttila M, Heinonen S. Effect of endometriosis on IVF/ICSI outcome: stage III/IV endometriosis worsens cumulative pregnancy and live-born rates. Hum Reprod. 2005;20: 3130–3135. 10.1093/humrep/dei176 16006468

[43] Barnhart K, Dunsmoor-Su R, Coutifaris C. Effect of endometriosis on in vitro fertilization. Fertil Steril. 2002;77: 1148–55. Available: http://www.ncbi.nlm.nih.gov/pubmed/12057720 10.1016/s0015-0282(02)03112-6 12057720

[44] Dyson MT, Roqueiro D, Monsivais D, Ercan CM, Pavone ME, Brooks DC, et al Genome-Wide DNA Methylation Analysis Predicts an Epigenetic Switch for GATA Factor Expression in Endometriosis. PLoS Genet. 2014;10 10.1371/journal.pgen.1004158 24603652 PMC3945170

[45] Maekawa R, Tamura I, Shinagawa M, Mihara Y, Sato S, Okada M, et al Genome-wide DNA methylation analysis revealed stable DNA methylation status during decidualization in human endometrial stromal cells. BMC Genomics. 2019;20 10.1186/s12864-019-5695-0 31035926 PMC6489213

[46] Laird PW. Principles and challenges of genome-wide DNA methylation analysis. Nat Rev Genet. 2010;11: 191 Available: 10.1038/nrg2732 20125086

[47] Lessey BA, Young SL. Homeostasis imbalance in the endometrium of women with implantation defects: The role of estrogen and progesterone. Semin Reprod Med. 2014;32: 365–375. 10.1055/s-0034-1376355 24959818

[48] Palomino WA, Tayade C, Argandoña F, Devoto L, Young SL, Lessey BA. The endometria of women with endometriosis exhibit dysfunctional expression of complement regulatory proteins during the mid secretory phase. J Reprod Immunol. 2018;125: 1–7. 10.1016/j.jri.2017.10.046 29153978

[49] Yoo JY, Kim TH, Fazleabas AT, Palomino WA, Ahn SH, Tayade C, et al KRAS Activation and over-expression of SIRT1/BCL6 Contributes to the Pathogenesis of Endometriosis and Progesterone Resistance. Sci Rep. 2017;7 10.1038/s41598-017-04577-w 28754906 PMC5533722

[50] Stocks MM, Crispens MA, Ding T, Mokshagundam S, Bruner-Tran KL, Osteen KG. Therapeutically Targeting the Inflammasome Product in a Chimeric Model of Endometriosis-Related Surgical Adhesions. Reprod Sci. 2017;24: 1121–1128. 10.1177/1933719117698584 28322132 PMC5933106

[51] Klemmt PAB, Carver JG, Kennedy SH, Koninckx PR, Mardon HJ. Stromal cells from endometriotic lesions and endometrium from women with endometriosis have reduced decidualization capacity. Fertil Steril. 2006;85: 564–572. 10.1016/j.fertnstert.2005.08.046 16500320 PMC1626574

[52] Osteen KG, Bruner-Tran KL, Eisenberg E. Reduced progesterone action during endometrial maturation: A potential risk factor for the development of endometriosis. Fertil Steril. 2005;83: 529–537. 10.1016/j.fertnstert.2004.11.026 15749474

[53] Bruner-Tran KL, Eisenberg E, Yeaman GR, Anderson TA, McBean J, Osteen KG. Steroid and Cytokine Regulation of Matrix Metalloproteinase Expression in Endometriosis and the Establishment of Experimental Endometriosis in Nude Mice. J Clin Endocrinol Metab. 2002;87: 4782–4791. 10.1210/jc.2002-020418 12364474

[54] McKinnon B, Mueller M, Montgomery G. Progesterone Resistance in Endometriosis: an Acquired Property? Trends Endocrinol Metab. 2018;29: 535–548. 10.1016/j.tem.2018.05.006 29934050

[55] Garrido-Gomez T, Dominguez F, Quiñonero A, Diaz-Gimeno P, Kapidzic M, Gormley M, et al Defective decidualization during and after severe preeclampsia reveals a possible maternal contribution to the etiology. Proc Natl Acad Sci. 2017;114: E8468–E8477. 10.1073/pnas.1706546114 28923940 PMC5635883

[56] Yang S, Fang Z, Suzuki T, Sasano H, Zhou J, Gurates B, et al Regulation of Aromatase P450 Expression in Endometriotic and Endometrial Stromal Cells by CCAAT/Enhancer Binding Proteins (C/EBPs): Decreased C/EBPβ in Endometriosis Is Associated with Overexpression of Aromatase. J Clin Endocrinol Metab. 2002;87: 2336–2345. 10.1210/jcem.87.5.8486 11994385

[57] Attar E, Bulun SE. Aromatase and other steroidogenic genes in endometriosis: translational aspects. Hum Reprod Update. 2006;12: 49–56. 10.1093/humupd/dmi034 16123052

[58] Kitawaki J, Noguchi T, Amatsu T, Maeda K, Tsukamoto K, Yamamoto T, et al Expression of Aromatase Cytochrome P450 Protein and Messenger Ribonucleic Acid in Human Endometriotic and Adenomyotic Tissues but not in Normal Endometrium1. Biol Reprod. 1997;57: 514–519. 10.1095/biolreprod57.3.514 9282984

[59] Zeitoun K, Takayama K, Michael MD, Bulun SE. Stimulation of aromatase P450 promoter (II) activity in endometriosis and its inhibition in endometrium are regulated by competitive binding of steroidogenic factor-1 and chicken ovalbumin upstream promoter transcription factor to the same cis-acting element. Mol Endocrinol. 1999;13: 239–253. 10.1210/mend.13.2.0229 9973254

[60] Thurman RE, Rynes E, Humbert R, Vierstra J, Maurano MT, Haugen E, et al The accessible chromatin landscape of the human genome. Nature. 2012;489: 75–82. 10.1038/nature11232 22955617 PMC3721348

[61] Andreu-Vieyra C, Lai J, Berman BP, Frenkel B, Jia L, Jones PA, et al Dynamic nucleosome-depleted regions at androgen receptor enhancers in the absence of ligand in prostate cancer cells. Mol Cell Biol. 2011;31: 4648–62. 10.1128/MCB.05934-11 21969603 PMC3232925

[62] Green S, Walter P, Kumar V, Krust A, Bornert J-M, Argos P, et al Human oestrogen receptor cDNA: sequence, expression and homology to v-erb-A. Nature. 1986;320: 134–139. 10.1038/320134a0 3754034

[63] Curtis Hewitt S, Collins J, Grissom S, Deroo B, Korach KS. Global Uterine Genomics *in Vivo*: Microarray Evaluation of the Estrogen Receptor α-Growth Factor Cross-Talk Mechanism. Mol Endocrinol. 2005;19: 657–668. 10.1210/me.2004-0142 15528273

[64] Tanos T, Rojo LJ, Echeverria P, Brisken C. ER and PR signaling nodes during mammary gland development. Breast Cancer Res. 2012;14: 210 10.1186/bcr3166 22809143 PMC3680919

[65] Hurtado A, Holmes KA, Ross-Innes CS, Schmidt D, Carroll JS. FOXA1 is a key determinant of estrogen receptor function and endocrine response. Nat Publ Gr. 2010;43 10.1038/ng.730 21151129 PMC3024537

[66] He HH, Meyer CA, Chen MW, Jordan VC, Brown M, Liu XS. Differential DNase I hypersensitivity reveals factor-dependent chromatin dynamics. Genome Res. 2012;22: 1015–25. 10.1101/gr.133280.111 22508765 PMC3371710

[67] He HH, Meyer CA, Shin H, Bailey ST, Wei G, Wang Q, et al Nucleosome dynamics define transcriptional enhancers. Nat Genet. 2010;42 10.1038/ng.545 20208536 PMC2932437

[68] Kato S, Endoh H, Masuhiro Y, Kitamoto T, Uchiyama S, Sasaki H, et al Activation of the estrogen receptor through phosphorylation by mitogen-activated protein kinase. Science (80-). 1995;270: 1491–1494. 10.1126/science.270.5241.1491 7491495

[69] Lupien M, Meyer CA, Bailey ST, Eeckhoute J, Cook J, Westerling T, et al Growth factor stimulation induces a distinct ER(alpha) cistrome underlying breast cancer endocrine resistance. Genes Dev. 2010;24: 2219–27. 10.1101/gad.1944810 20889718 PMC2947773

[70] Large MJ, Wetendorf M, Lanz RB, Hartig SM, Creighton CJ, Mancini MA, et al The Epidermal Growth Factor Receptor Critically Regulates Endometrial Function during Early Pregnancy. Stewart C, editor. PLoS Genet. 2014;10: e1004451 10.1371/journal.pgen.1004451 24945252 PMC4063709

[71] Erikson DW, Chen JC, Piltonen TT, Conti M, Irwin JC, Giudice LC, et al Inhibition of epidermal growth factor receptor restores decidualization markers in stromal fibroblasts from women with endometriosis. J Endometr. 2014;6: 196–211. 10.5301/je.5000198

[72] Berno V, Amazit L, Hinojos C, Zhong J, Mancini MG, Sharp ZD, et al Activation of estrogen receptor-alpha by E2 or EGF induces temporally distinct patterns of large-scale chromatin modification and mRNA transcription. PLoS One. 2008;3: e2286 10.1371/journal.pone.0002286 18509470 PMC2386239

[73] Noyes RW, Hertig AT, Rock J. Dating the endometrial biopsy. Am J Obstet Gynecol. 1975;122: 262–263. 10.1016/s0002-9378(16)33500-1 1155504

[74] American Society for Reproductive AS for R. Revised American Society for Reproductive Medicine classification of endometriosis: 1996. Fertil Steril. 1997;67: 817–821. 10.1016/s0015-0282(97)81391-x 9130884

[75] Sheldon E, Vo KC, McIntire RA, Aghajanova L, Zelenko Z, Irwin JC, et al Biobanking human endometrial tissue and blood specimens: standard operating procedure and importance to reproductive biology research and diagnostic development. Fertil Steril. 2011;95: 2120–2122.e12. 10.1016/j.fertnstert.2011.01.164 21371706 PMC3080464

[76] Irwin JC, Kirk D, King RJB, Quigley MM, Gwatkin RBL. Hormonal regulation of human endometrial stromal cells in culture: an in vitro model for decidualization. Fertil Steril. 1989;52: 761–768. 10.1016/s0015-0282(16)61028-2 2806617

[77] Bell SC, Jackson JA, Ashmore J, Zhu HH, Tseng L. Regulation of Insulin-Like Growth Factor-Binding Protein-1 Synthesis and Secretion by Progestin and Relaxin in Long Term Cultures of Human Endometrial Stromal Cells*. J Clin Endocrinol Metab. 1991;72: 1014–1024. 10.1210/jcem-72-5-1014 1708779

[78] Campan M, Weisenberger DJ, Trinh B, Laird PW. MethyLight. Methods in molecular biology (Clifton, NJ). 2009 pp. 325–337. 10.1007/978-1-59745-522-0_23 18987824

[79] Triche TJ, Weisenberger DJ, Van Den Berg D, Laird PW, Siegmund KD. Low-level processing of Illumina Infinium DNA Methylation BeadArrays. Nucleic Acids Res. 2013;41: e90–e90. 10.1093/nar/gkt090 23476028 PMC3627582

