# Supplementary material for: Steroid hormones regulate genome-wide epigenetic programming and gene transcription in human endometrial cells with marked aberrancies in endometriosis
Source: PLoS Genet. 2020 Jun 17;16(6):e1008601. doi: 10.1371/journal.pgen.1008601 (PMC7299312; doi:10.1371/journal.pgen.1008601)
Supplement: S1 Fig — (PDF) [file pgen.1008601.s001.pdf]

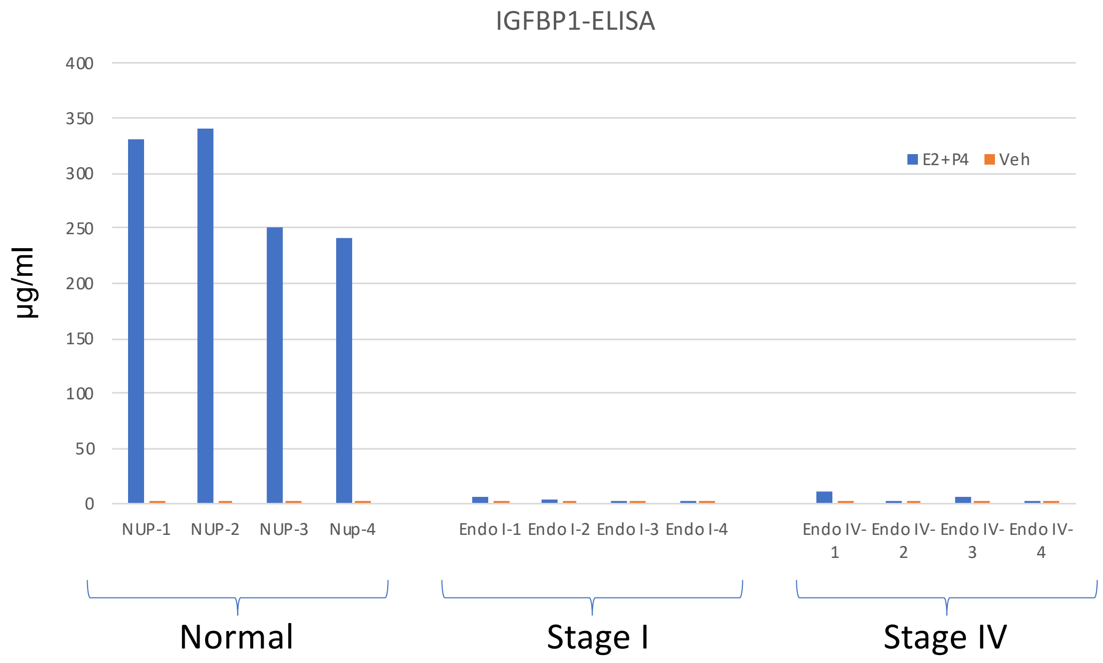

Supplemental Figure 1. IGFBP1 ELISA assay of normal, stage I and IV eSFs used in the study. IGFBP1, as a marker for decidualization, was assessed in the conditioned media of eSF treated with E_2+P_4 (Blue) or vehicle (orange) (0.1% ethanol) for 15 days, in duplicate which were averaged, and corrected to cell number. All normal eSF show robust decidualization (based on IGFBP1 marker product and morphological changes assessed by microscopy), while all disease eSF (stage I and IV) show virtually no decidualization.
