# Supplementary material for: Steroid hormones regulate genome-wide epigenetic programming and gene transcription in human endometrial cells with marked aberrancies in endometriosis
Source: PLoS Genet. 2020 Jun 17;16(6):e1008601. doi: 10.1371/journal.pgen.1008601 (PMC7299312; doi:10.1371/journal.pgen.1008601)

Supplementary Figure 2. Genomic distribution of differentially methylated CpG sites in each hormonal treatment, by gain or loss methylation, in normal (NUP), stage I (Endo I) and stage IV (Endo IV) eSF. Only enrichments with $p<0.05$ by z-test are shown. Orange line: gain of methylation; Blue line: loss of methylation; black line: total interrogated in the HM450 platform.

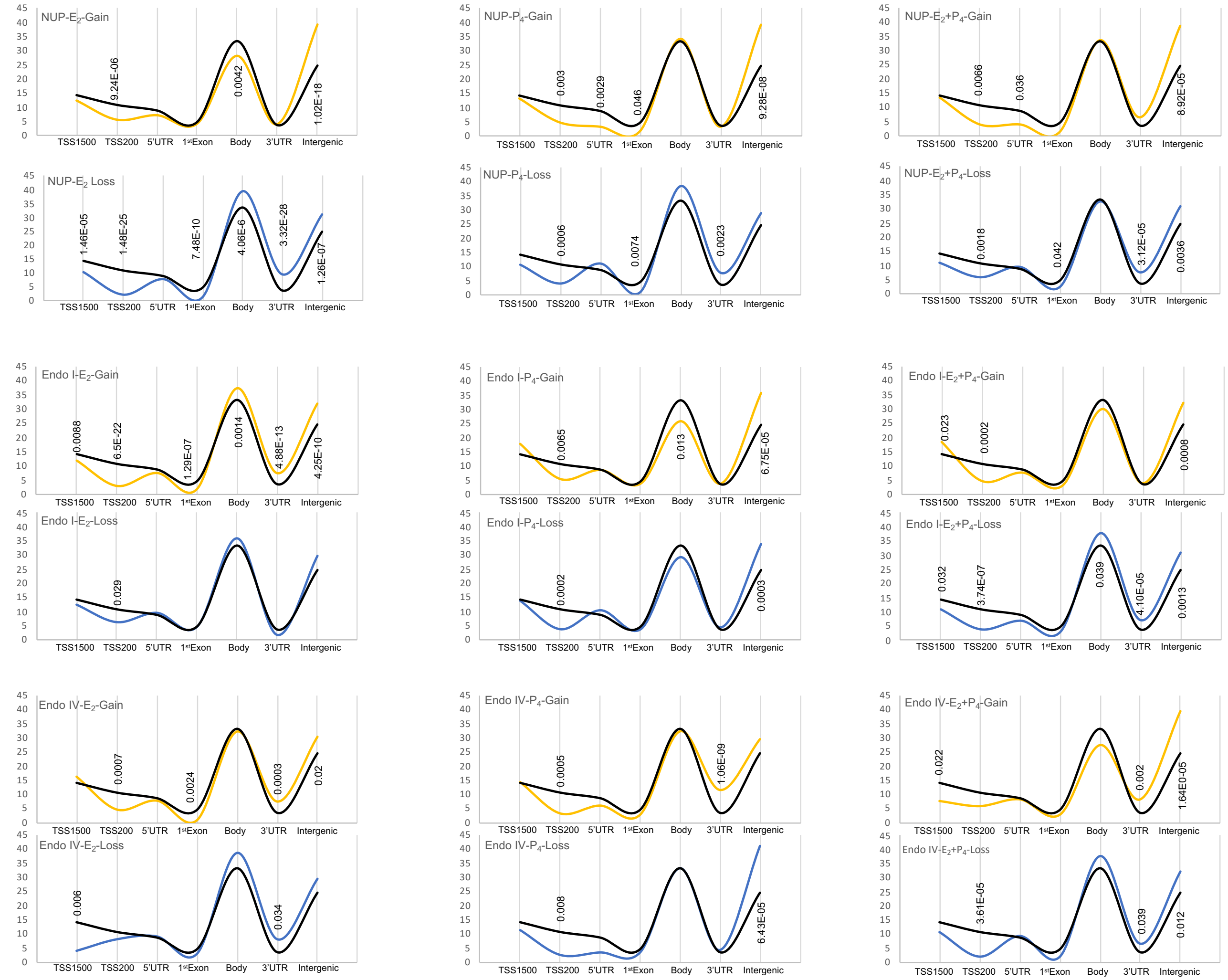

Supplement: S2 Fig — (PDF) [file pgen.1008601.s002.pdf]
