# Supplementary material for: Steroid hormones regulate genome-wide epigenetic programming and gene transcription in human endometrial cells with marked aberrancies in endometriosis
Source: PLoS Genet. 2020 Jun 17;16(6):e1008601. doi: 10.1371/journal.pgen.1008601 (PMC7299312; doi:10.1371/journal.pgen.1008601)

Supplementary Figure 3. Pre-existing aberrancies in stage I and stage IV eSF. **A.** Heatmap of differentially methylated CpG Sites in eSF from normal (NUP), stage I (E-I), stage IV (E-IV), with *no hormonal treatments.* Samples are shown in columns and loci in rows. Tertile is sorted based on increased median beta values in stage IV. **B.** Percent pre-existing signatures in stage I and stage IV.

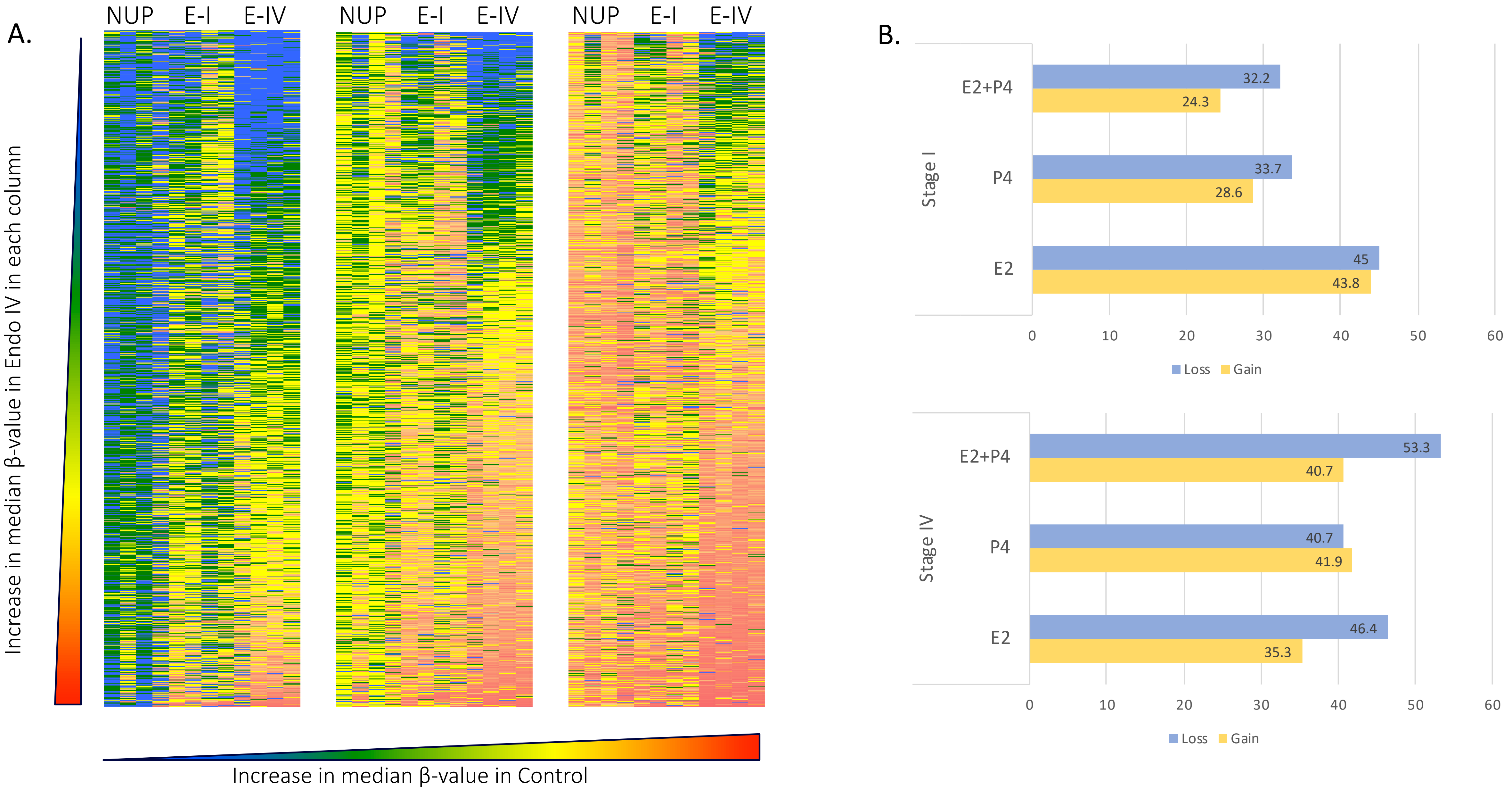

Supplement: S3 Fig — (PDF) [file pgen.1008601.s003.pdf]
