# Supplementary material for: Steroid hormones regulate genome-wide epigenetic programming and gene transcription in human endometrial cells with marked aberrancies in endometriosis
Source: PLoS Genet. 2020 Jun 17;16(6):e1008601. doi: 10.1371/journal.pgen.1008601 (PMC7299312; doi:10.1371/journal.pgen.1008601)

Supplementary Data 1. Differentially methylated loci and the associated pathways/biofunctions that are affected in hormonal treatments of eSF_{normal} , $eSF_{stage\ I}$ and $eSF_{stage\ IV}$ with known roles/importance in normal endometrial function and dysfunction in endometriosis. Pathways with important roles in endometrial biology and often affected in endometriosis include RAS, MAPK, ERBB, VEGF, JAK-STAT, FOXO1 signaling pathways among other biofuctions involving apoptosis, cell junction and estrogen signaling, and as depicted in these pathways are affected differently in normal, stage I and stage IV in response to hormones. Red star indicates genes that are differentially methylated in the specific pathways affected in each hormonal treatment in eSF_{normal} , $eSF_{stage\ I,}$ and $eSF_{stage\ IV}$.

eSF_{normal}

E_2 induced differentially methylated genes

Ras Signaling Pathway

★ Genes differentially methylated

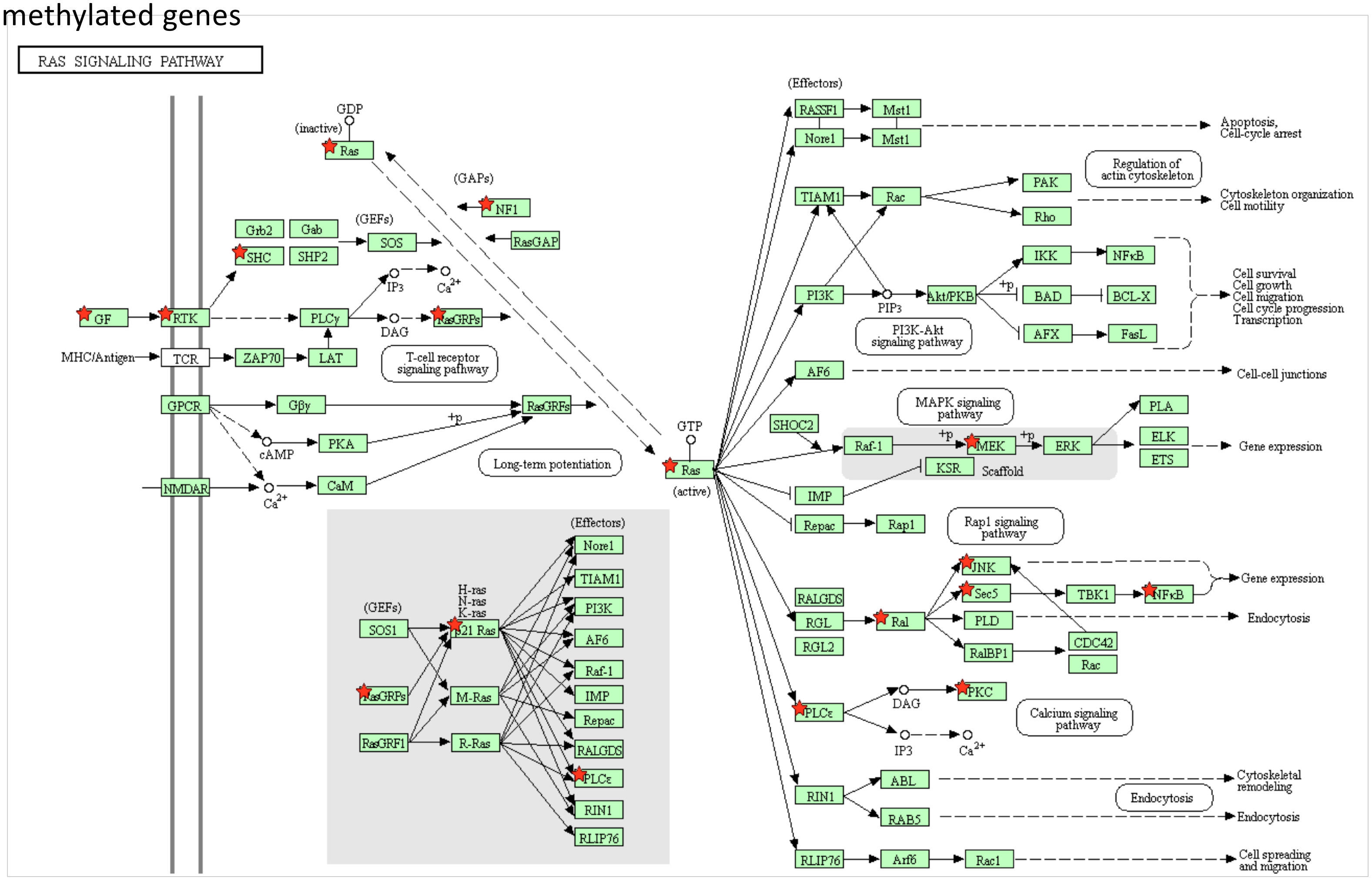

eSF$_{normal}$

E$_2$ induced differentially methylated genes

BRAF/MAPK pathway

★ Genes differentially methylated

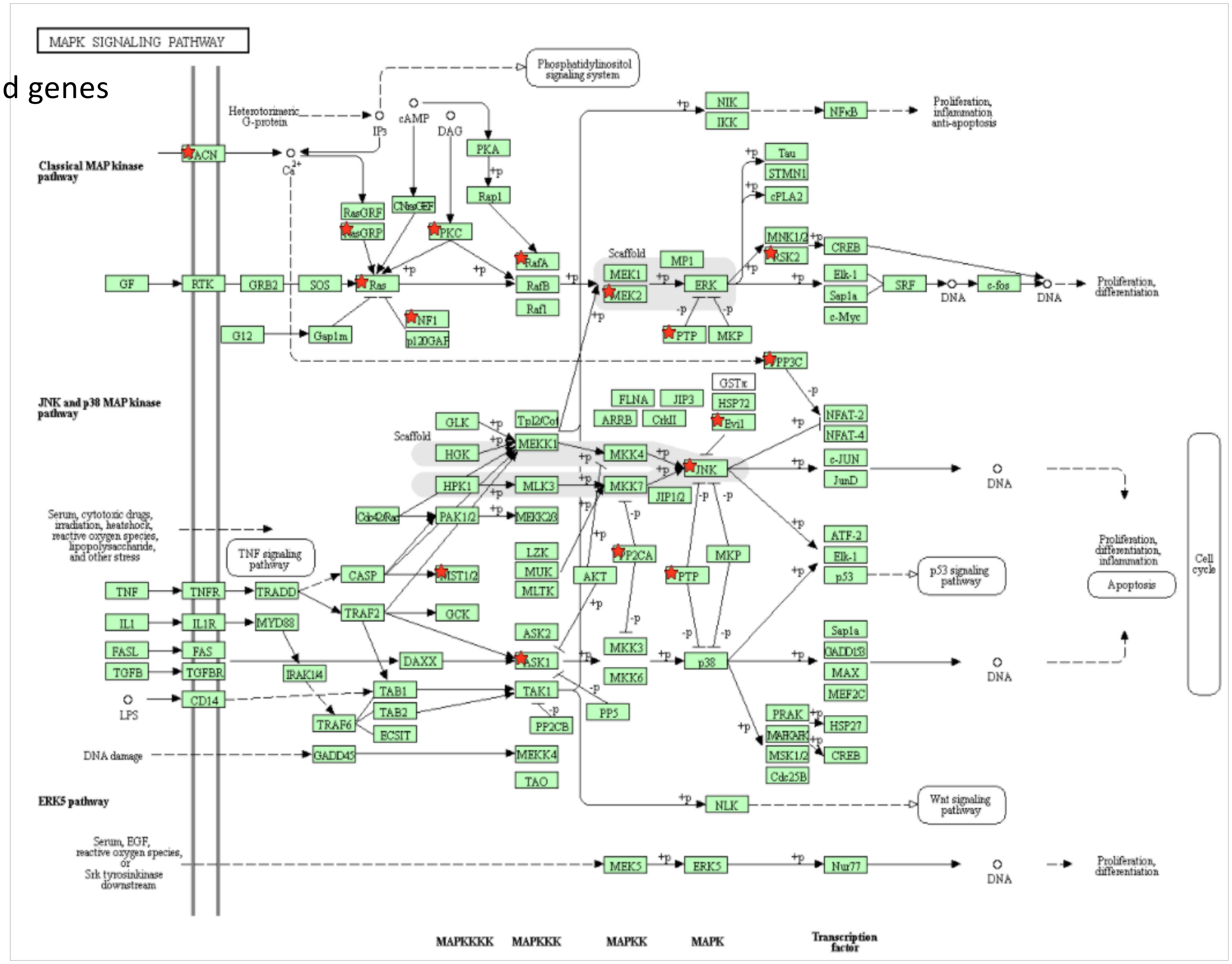

eSF$_{normal}$

E$_2$ induced differentially methylated genes

ERBB signaling pathway

★ Genes differentially methylated

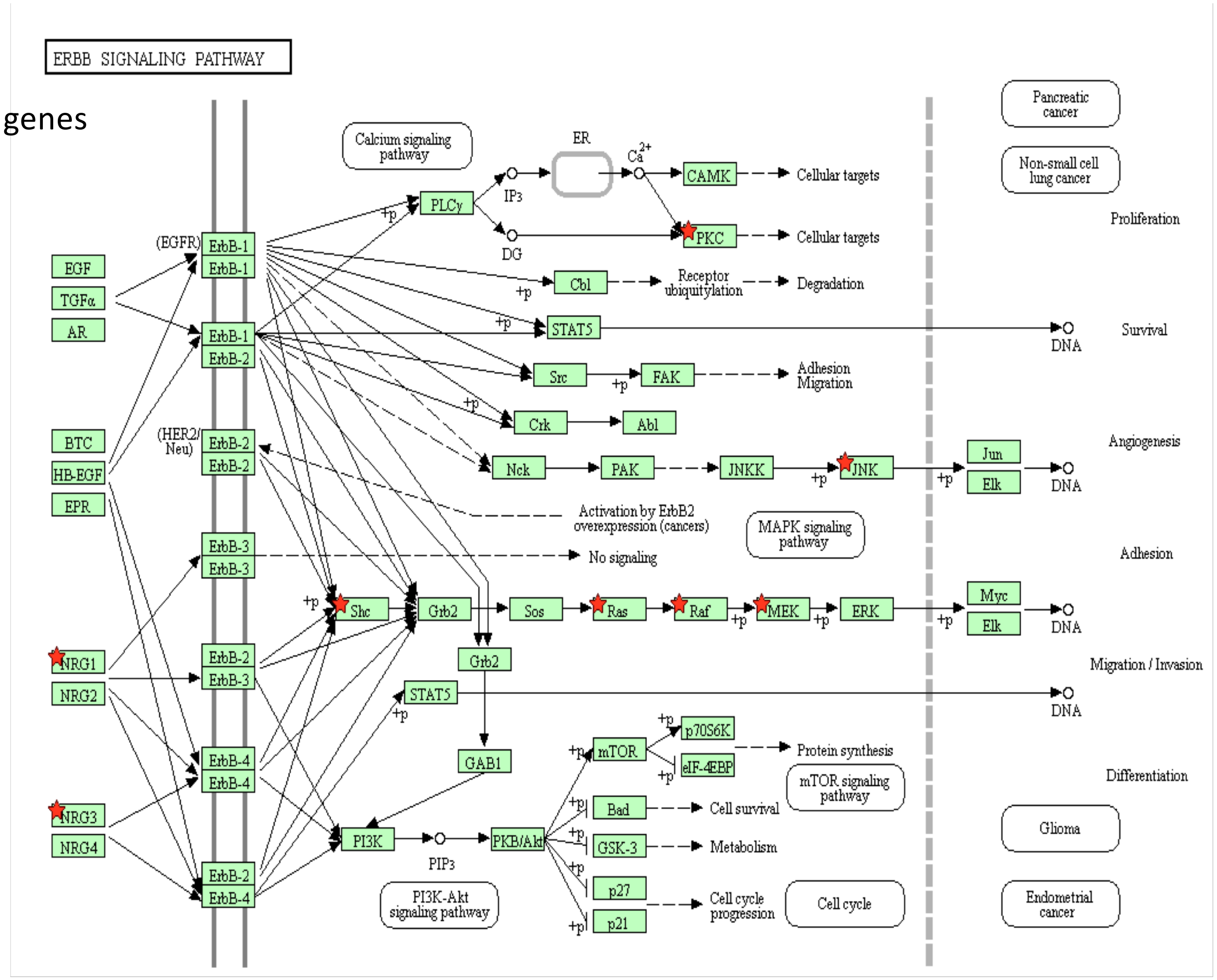

eSF$_{normal}$
E$_2$ induced differentially methylated genes
FOXO signaling pathway

★ Genes differentially methylated

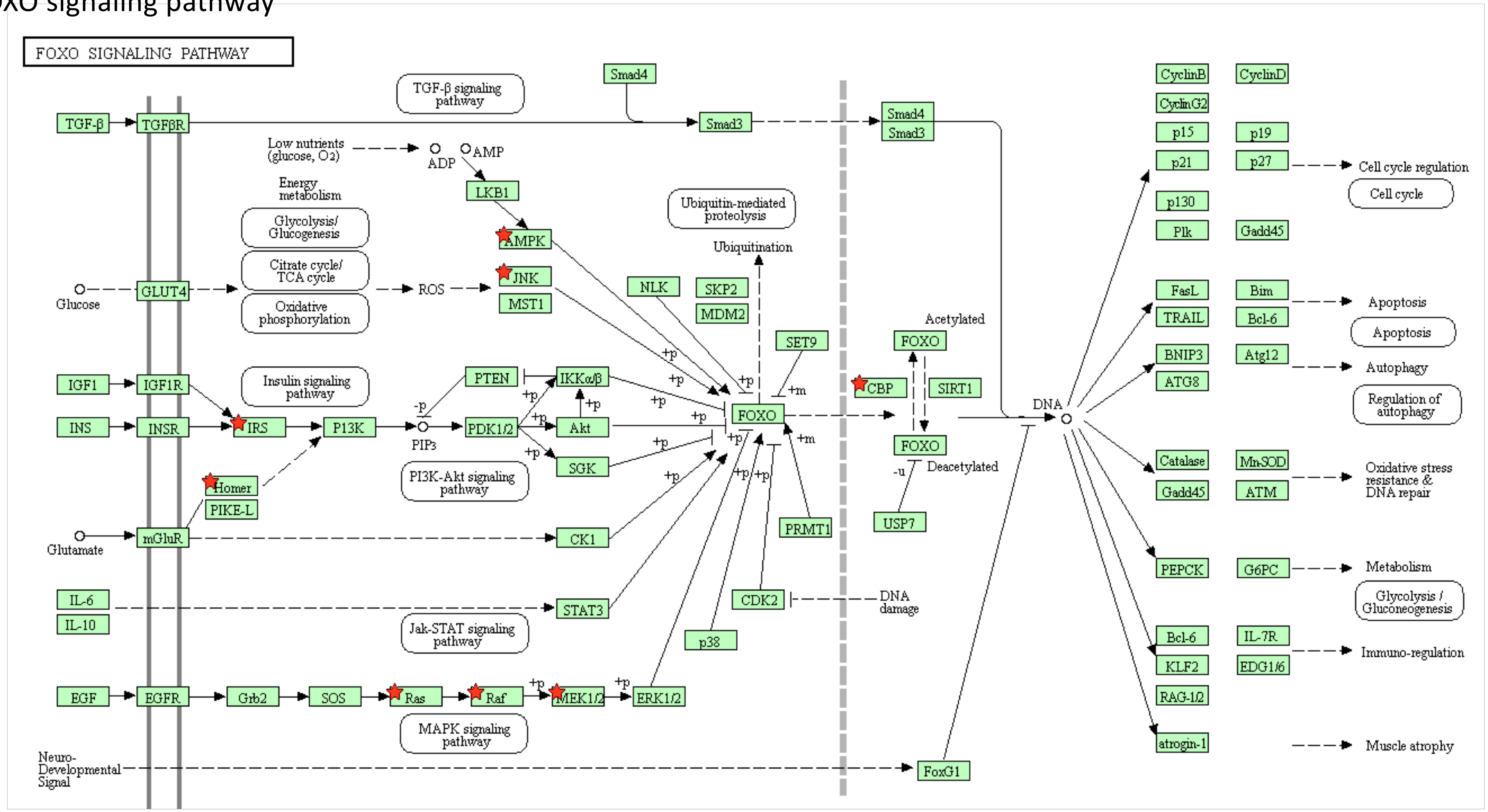

eSF$_{normal}$

E_2 induced differentially methylated genes

Notch signaling pathway

★ Genes differentially methylated

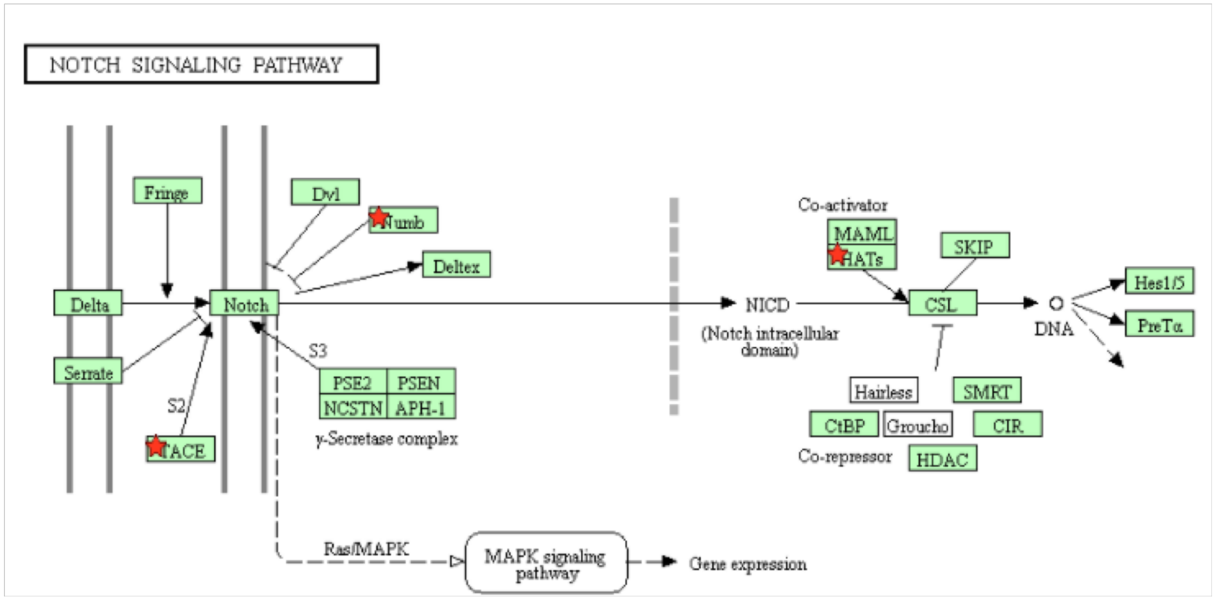

eSF$_{normal}$

E$_2$ induced differentially methylated genes

Thyroid hormone signaling

★ Genes differentially methylated

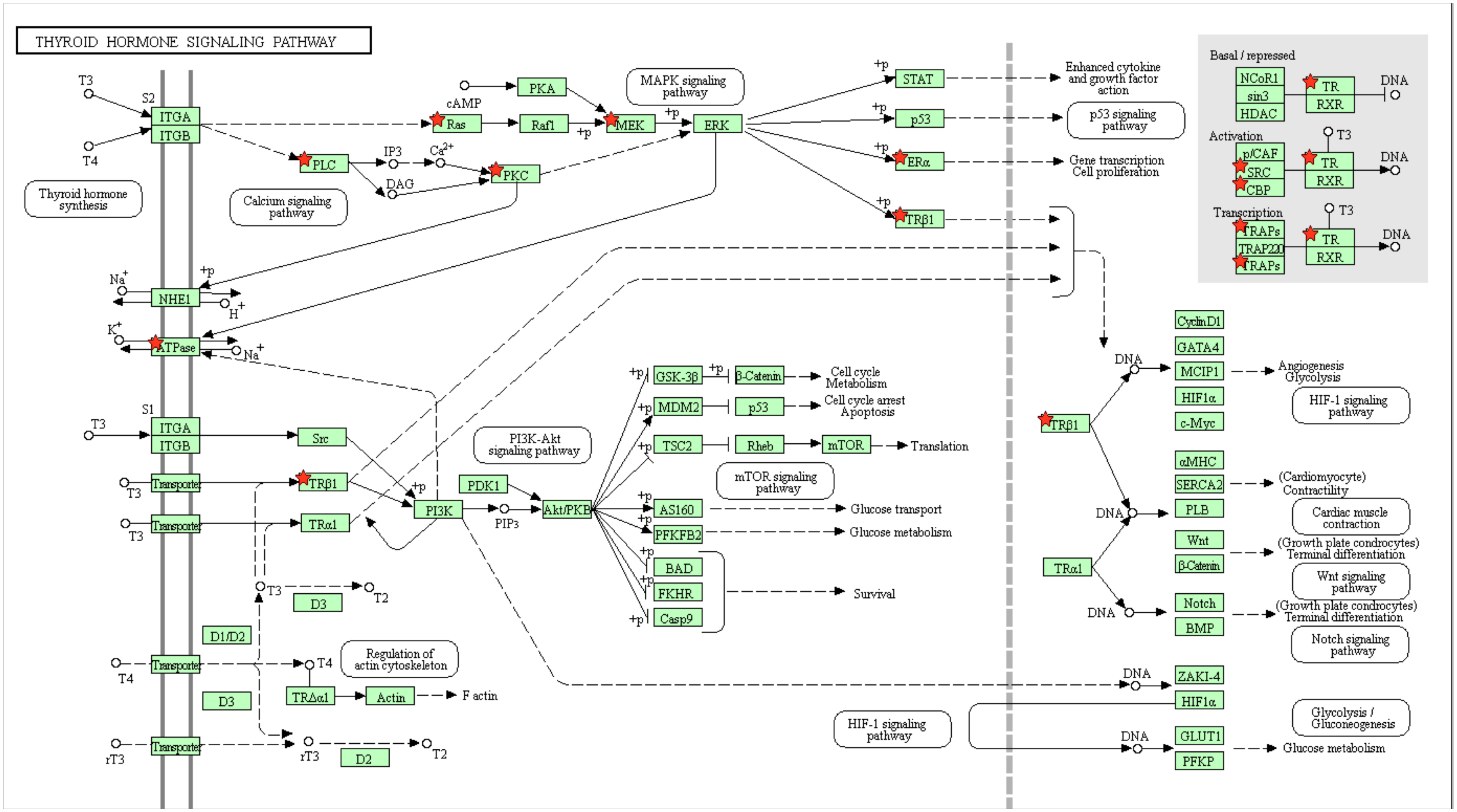

eSF_{normal}

E_2 induced differentially methylated genes

Endocrine pathway affecting Ca++ absorption

★ Genes differentially methylated

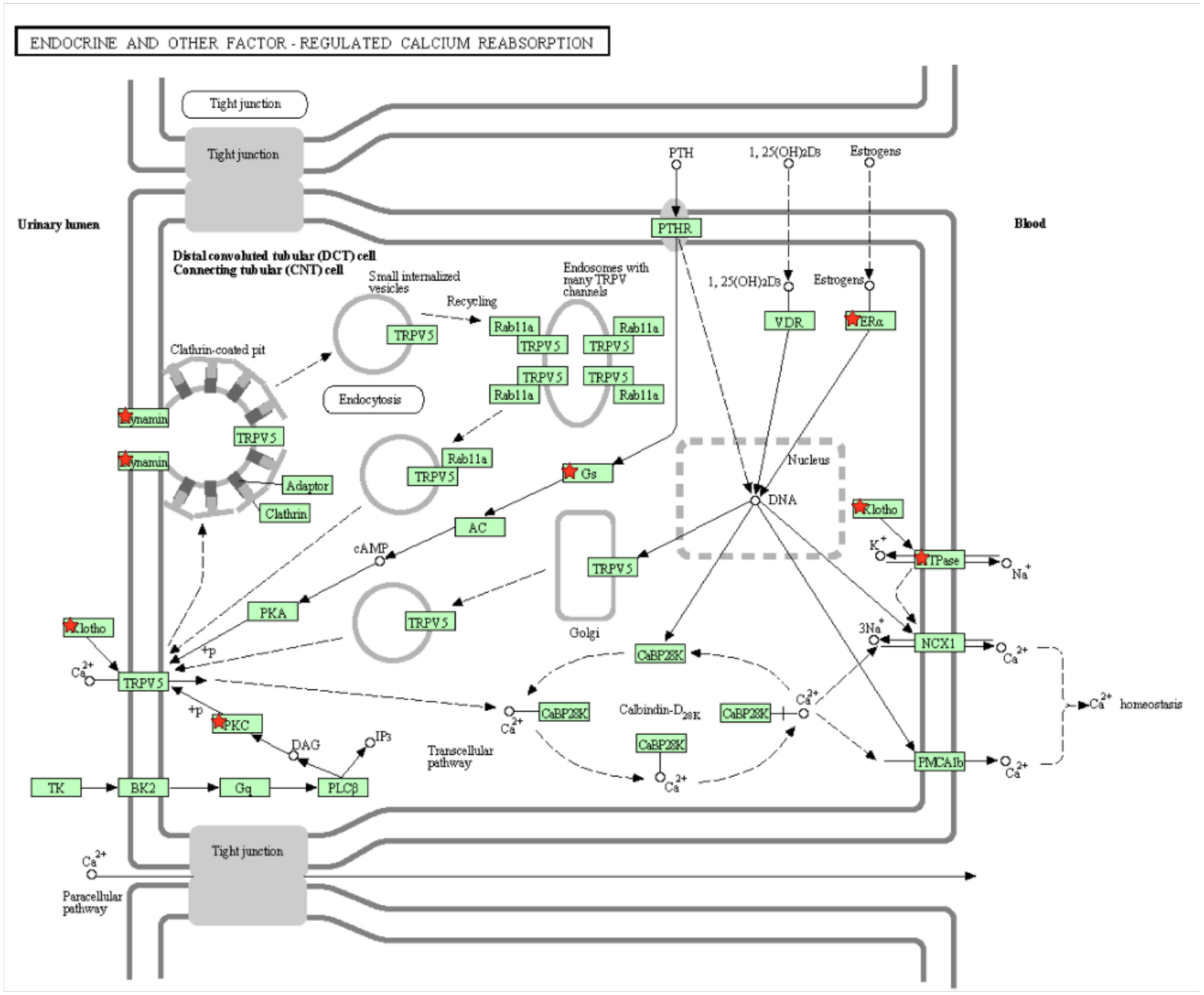

eSF$_{normal}$

E_2 induced differentially methylated genes

Genes involved in endometrial cancer

★ Genes differentially methylated

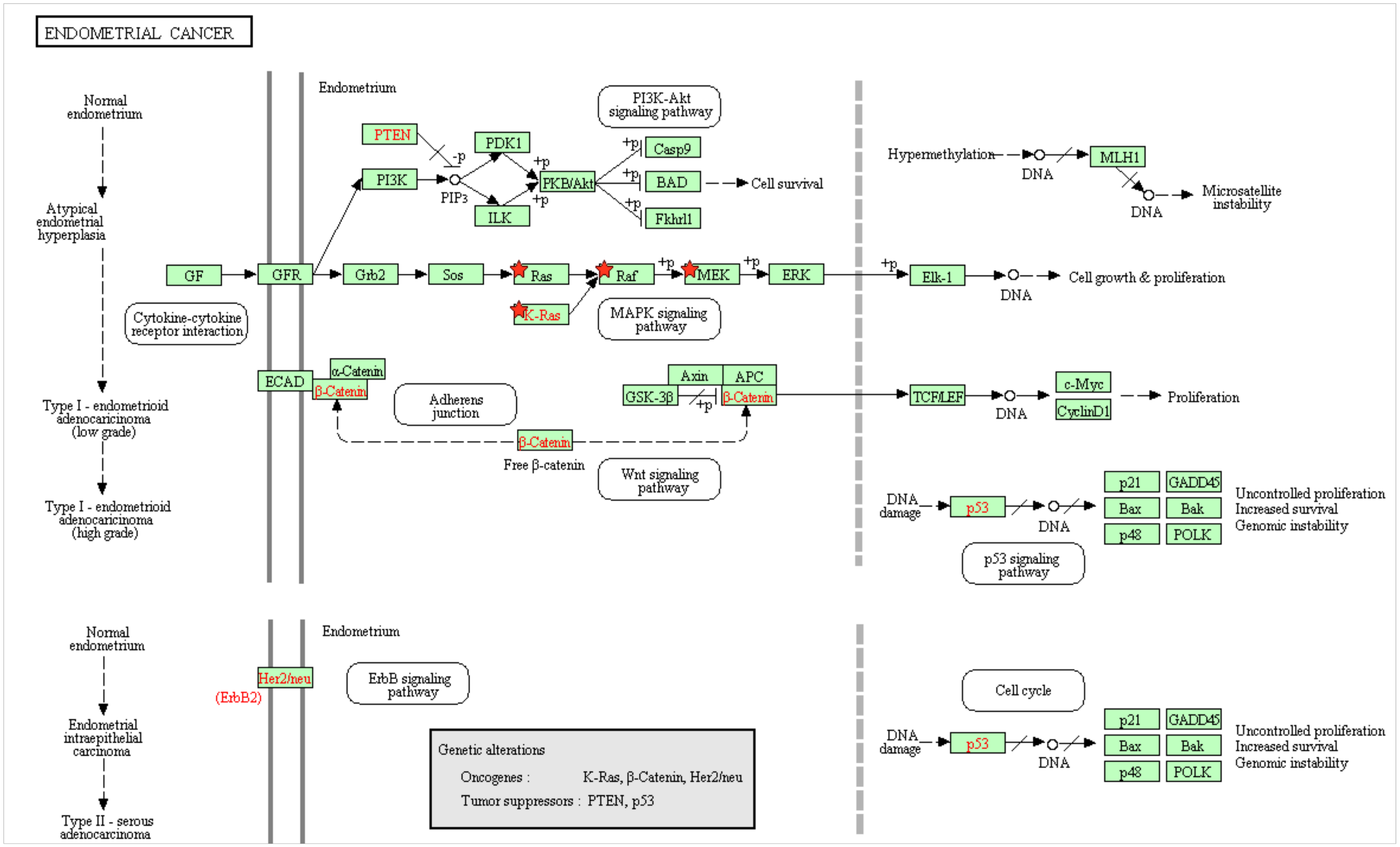

eSF$_{normal}$

E$_2$ induced differentially methylated genes

MicoRNAs involved in breast, ovarian and prostate cancers

★ Genes differentially methylated

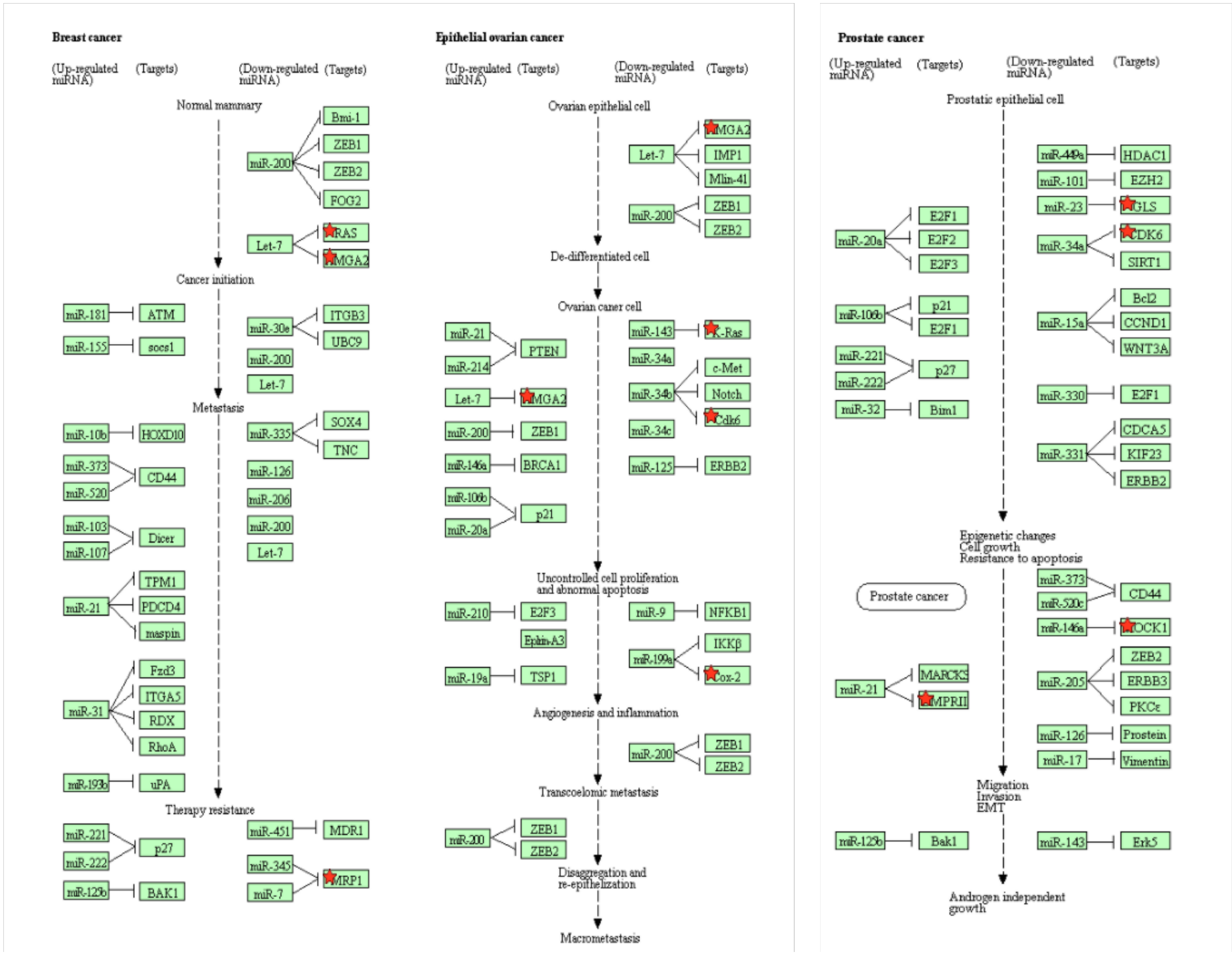

eSF$_{normal}$
P$_4$ induced differentially methylated genes
PI3K/AKT signaling pathway

★ Genes differentially methylated

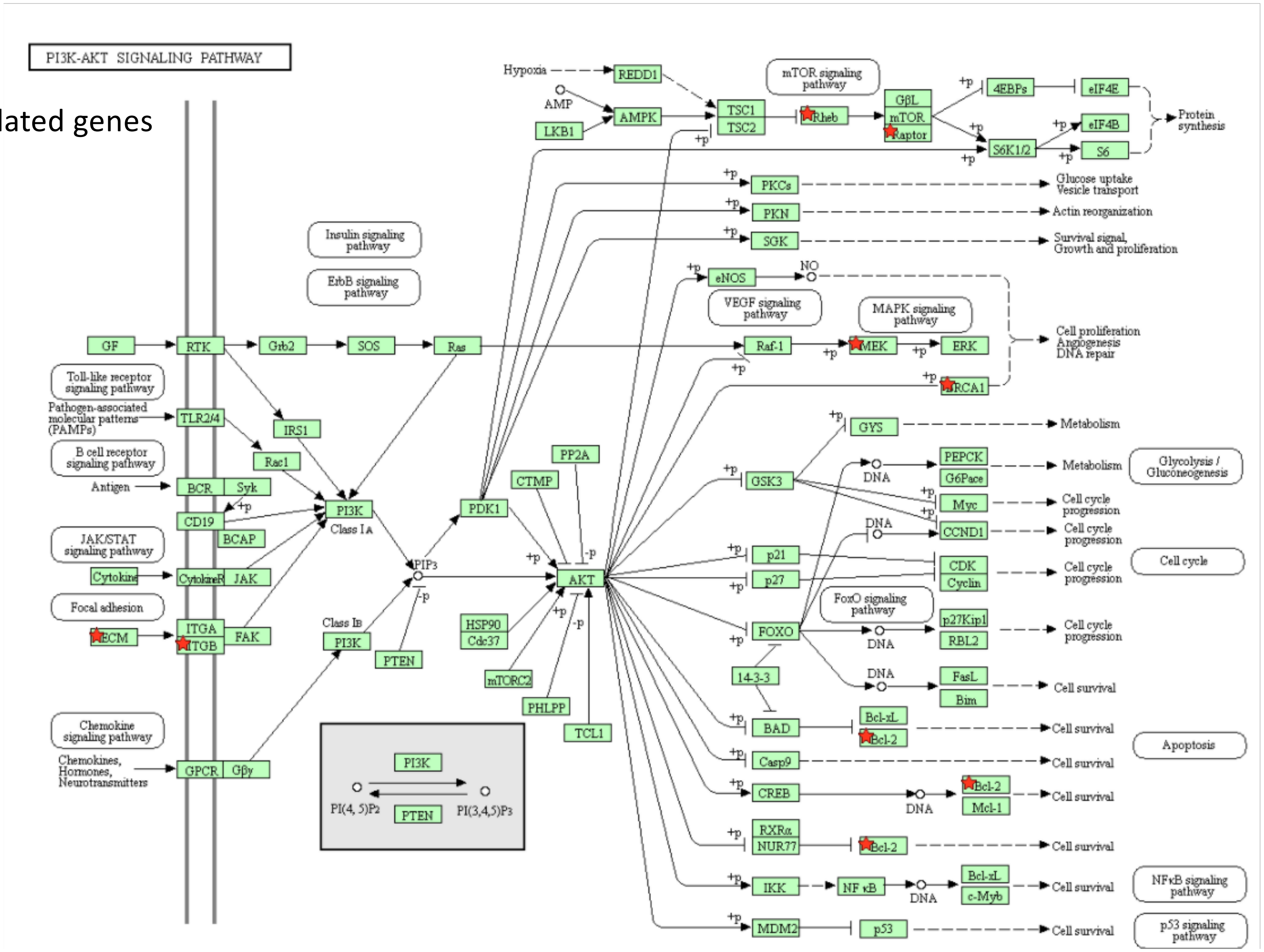

eSF$_{normal}$

P$_4$ induced differentially methylated genes

MAPK signaling pathway

★ Genes differentially methylated

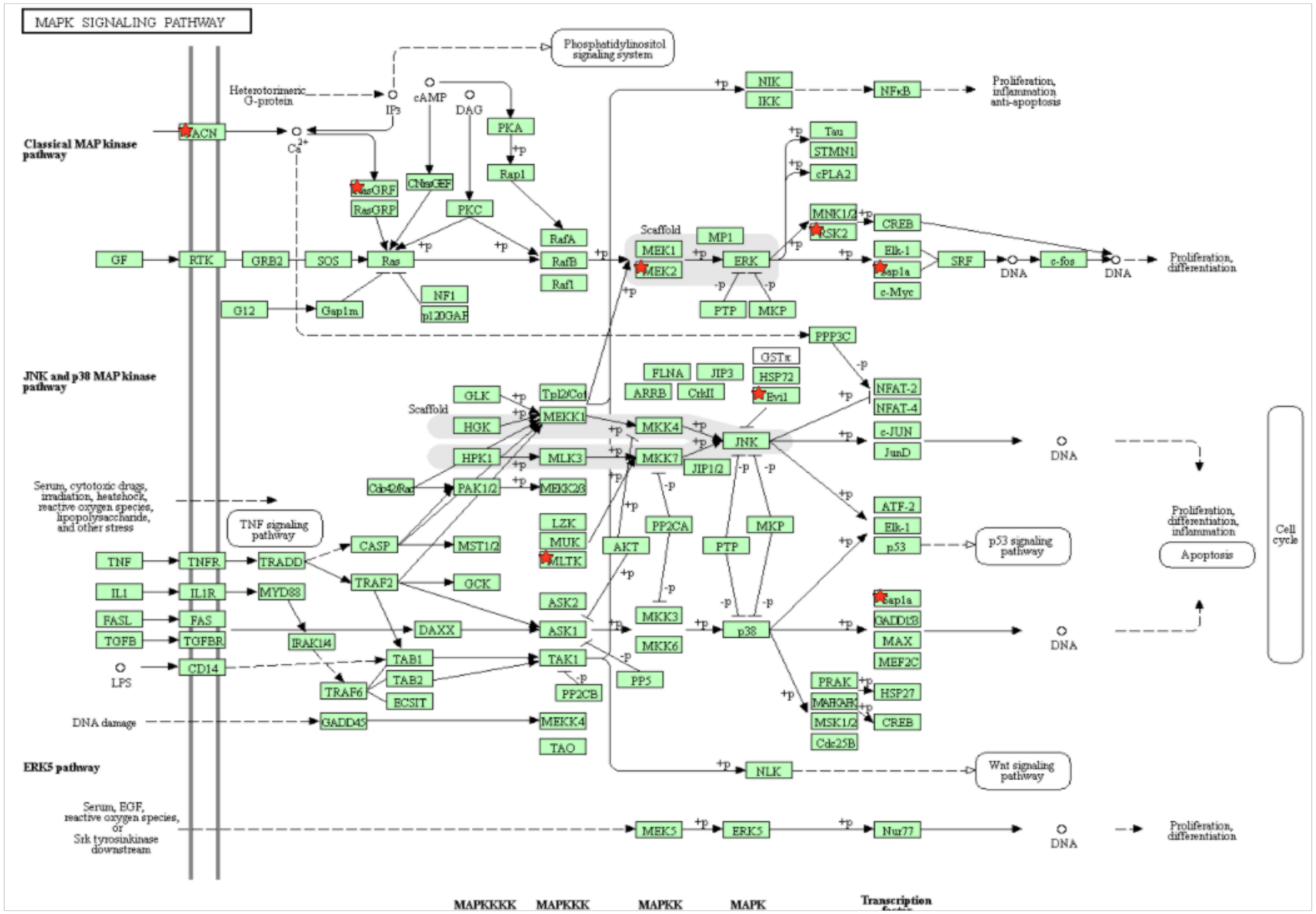

eSF$_{normal}$

P$_4$ induced differentially methylated genes

Regulation of Actin cytoskeleton

★ Genes differentially methylated

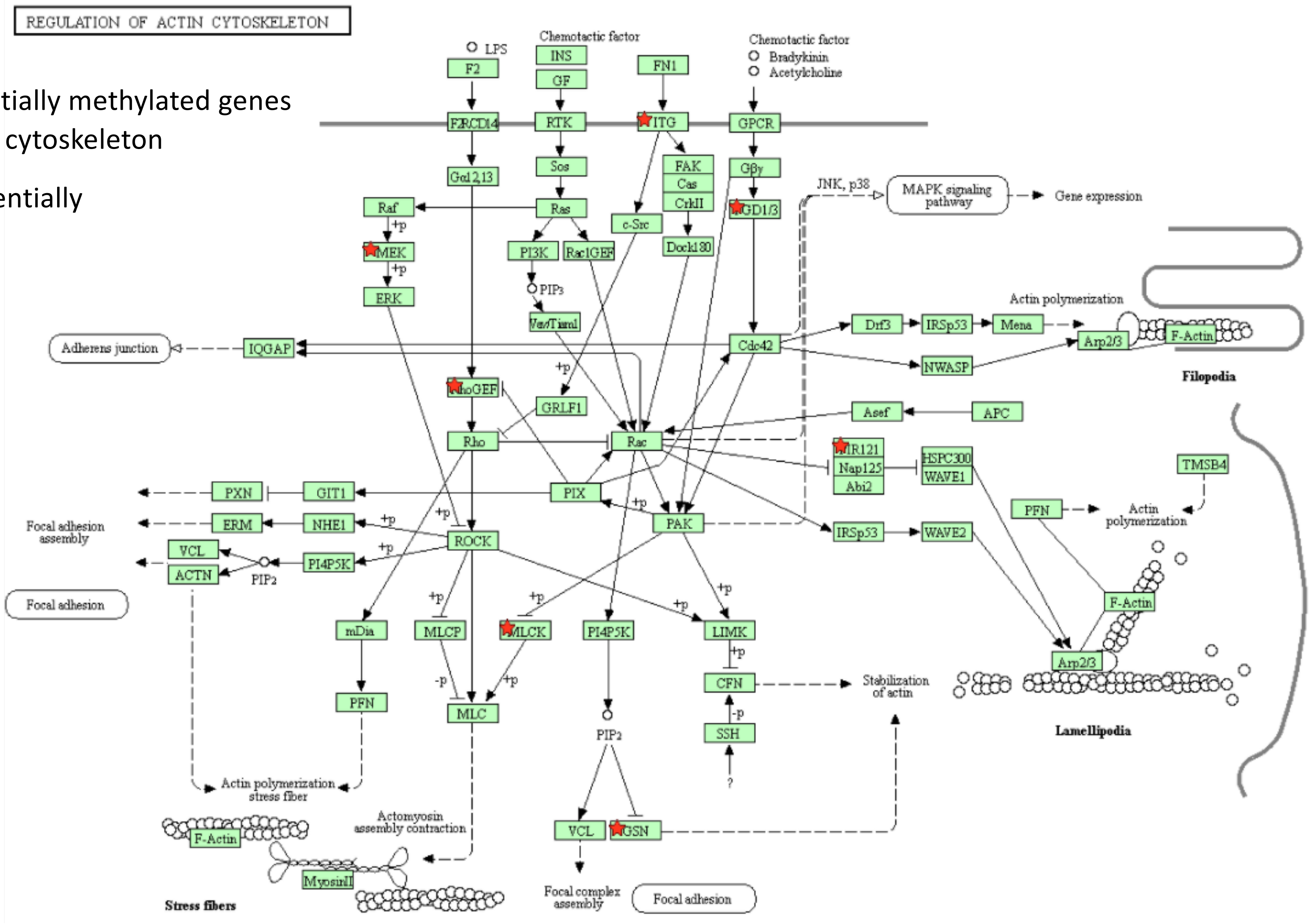

eSF$_{normal}$

P_4 induced differentially methylated genes

Oxidative stress

★ Genes differentially methylated

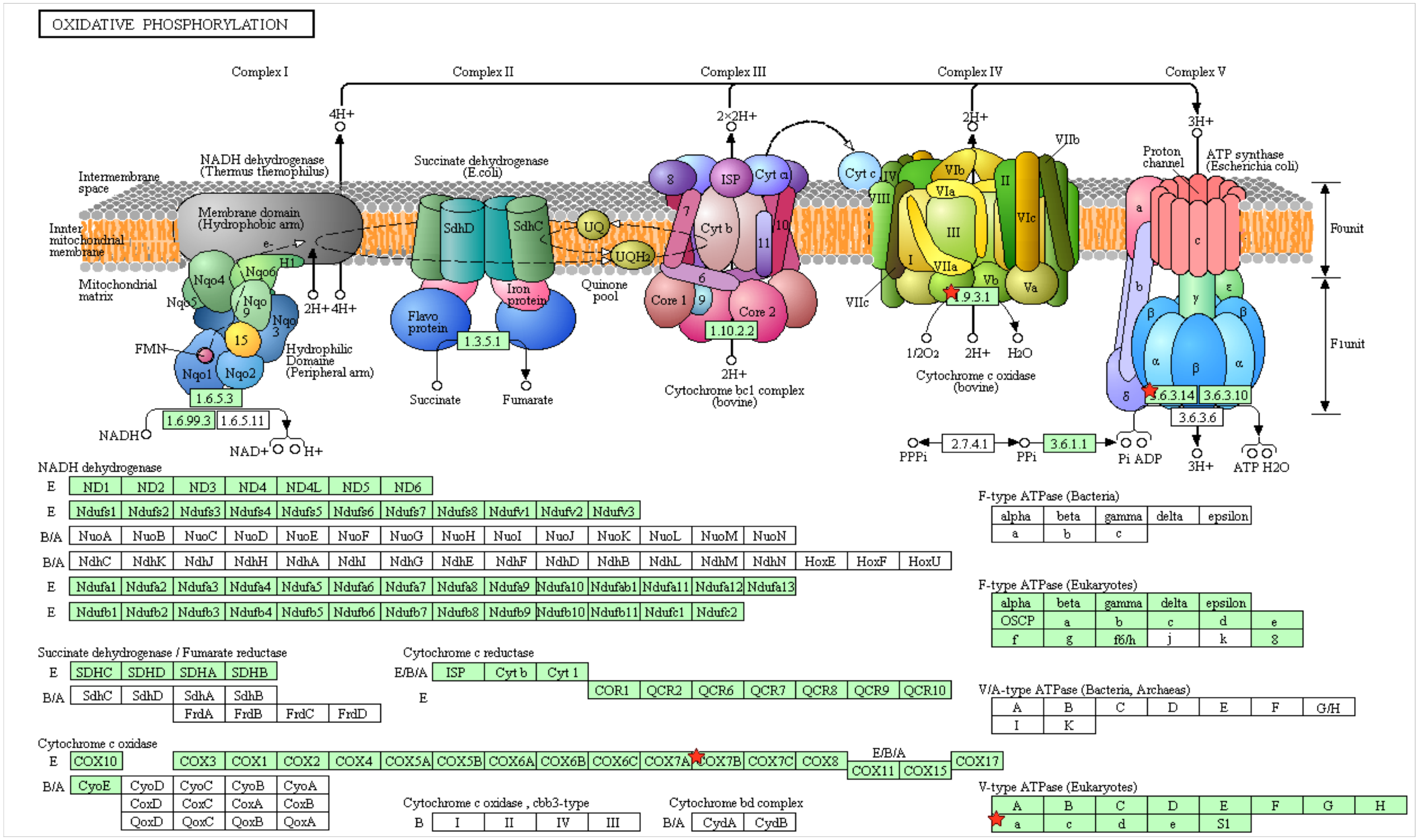

eSF$_{normal}$
P$_4$ induced differentially methylated genes
NF-kB signaling pathway

★ Genes differentially methylated

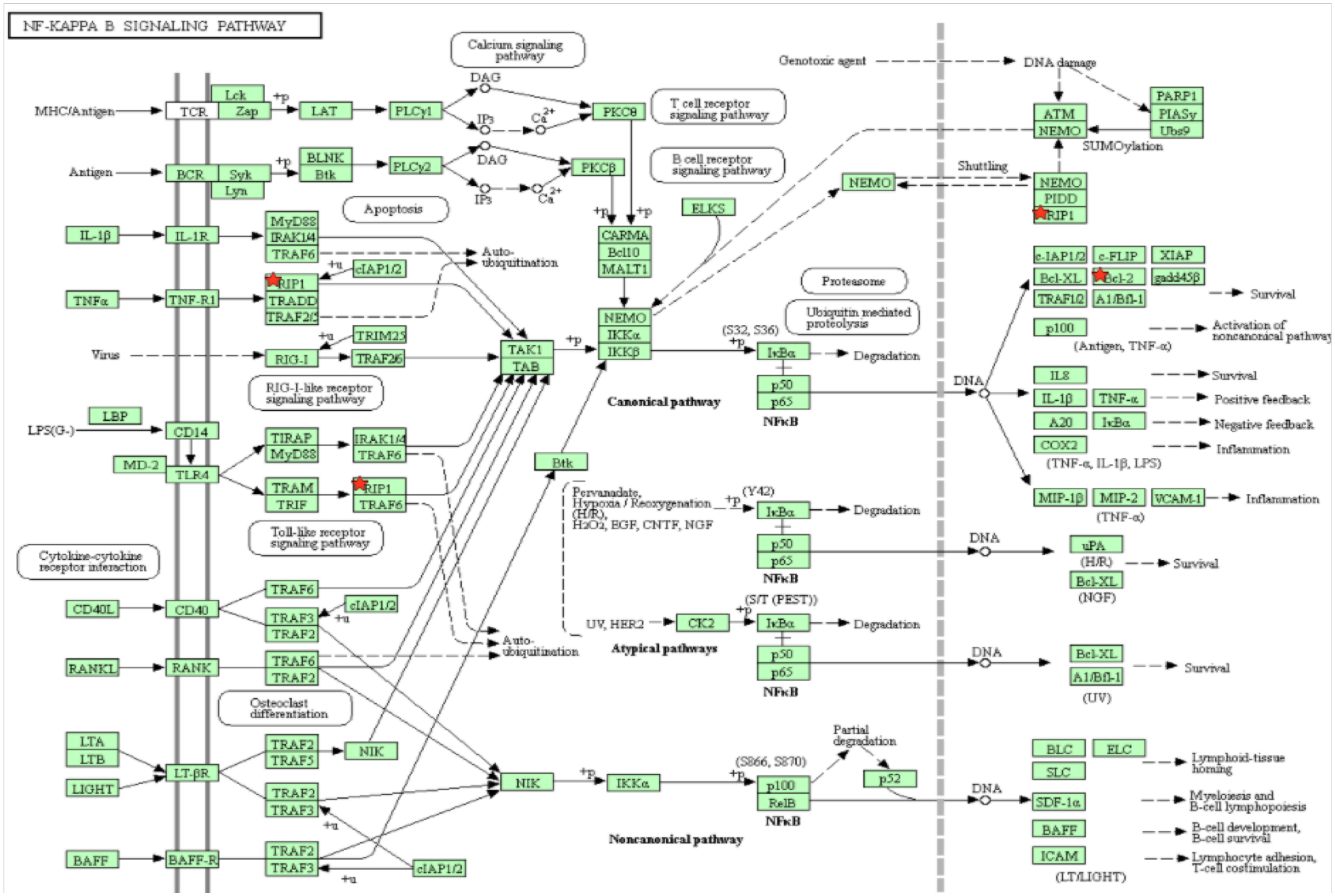

eSF$_{normal}$

P$_4$ induced differentially methylated genes

Apoptosis

★ Genes differentially methylated

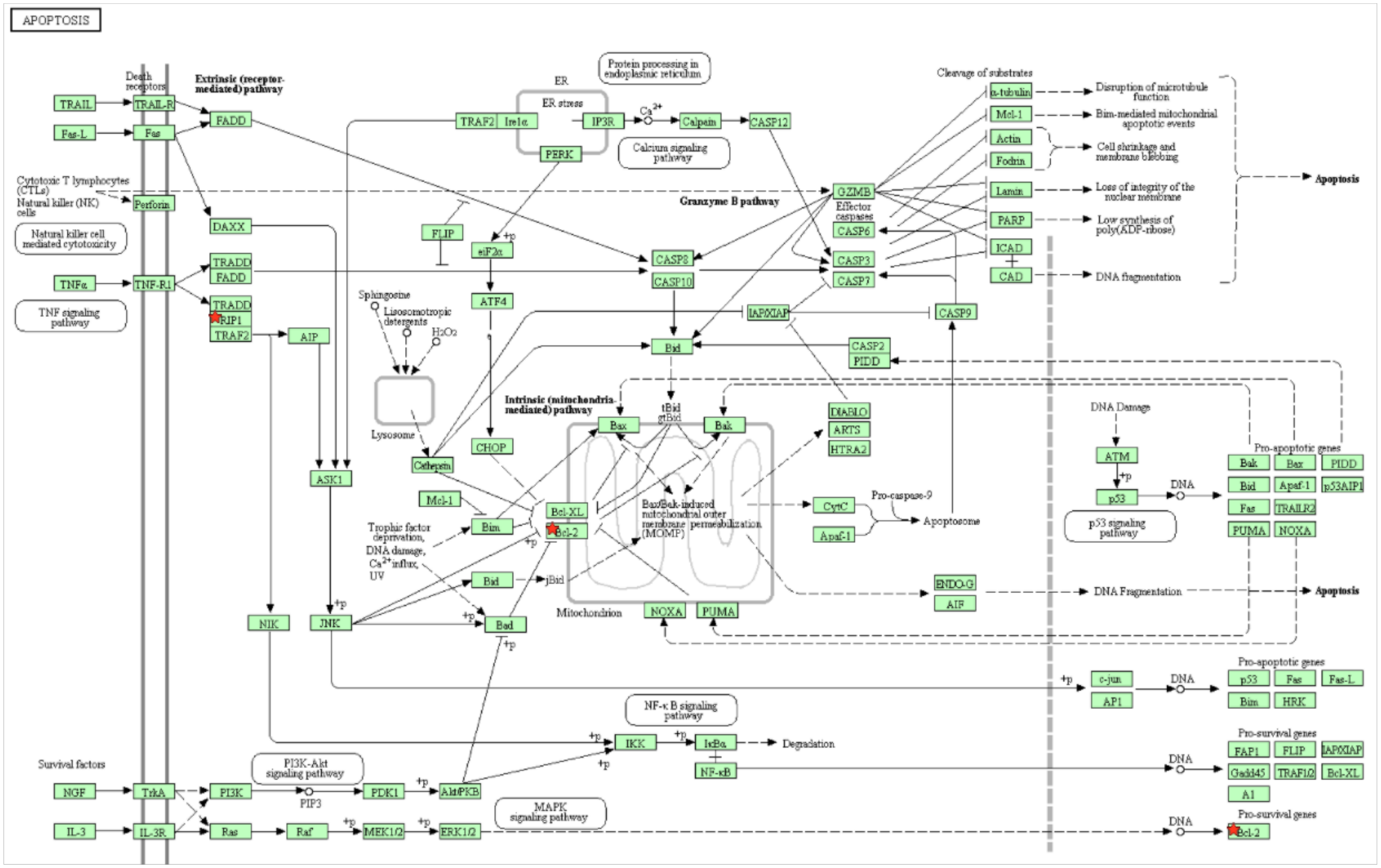

eSF$_{normal}$
P$_4$ induced
differentially methylated genes Cytokine-Cytokine interaction

★ Genes differentially methylated

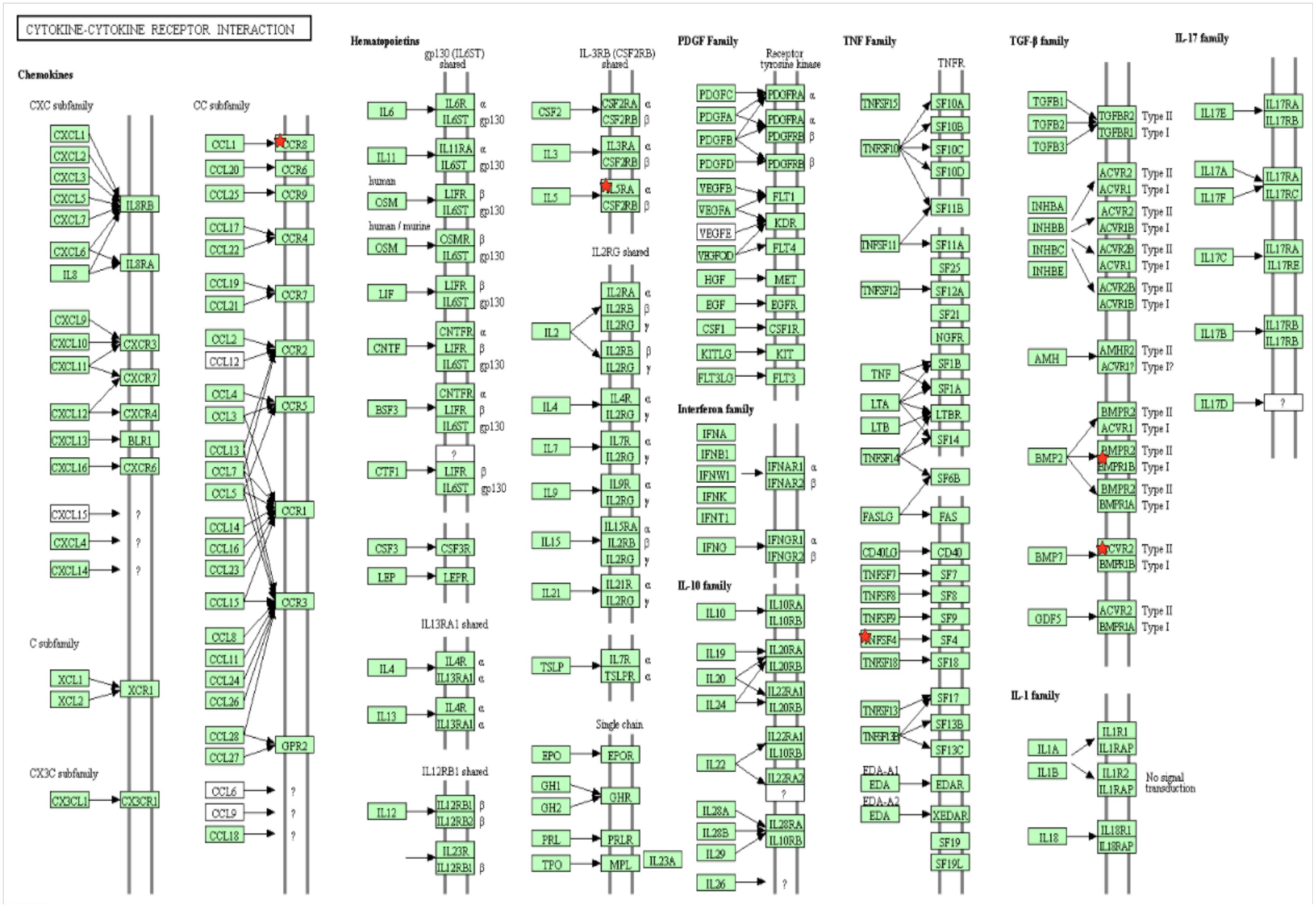

eSF$_{normal}$
P$_4$ induced differentially methylated genes
MicroRNAs in cancer

★ Genes differentially methylated

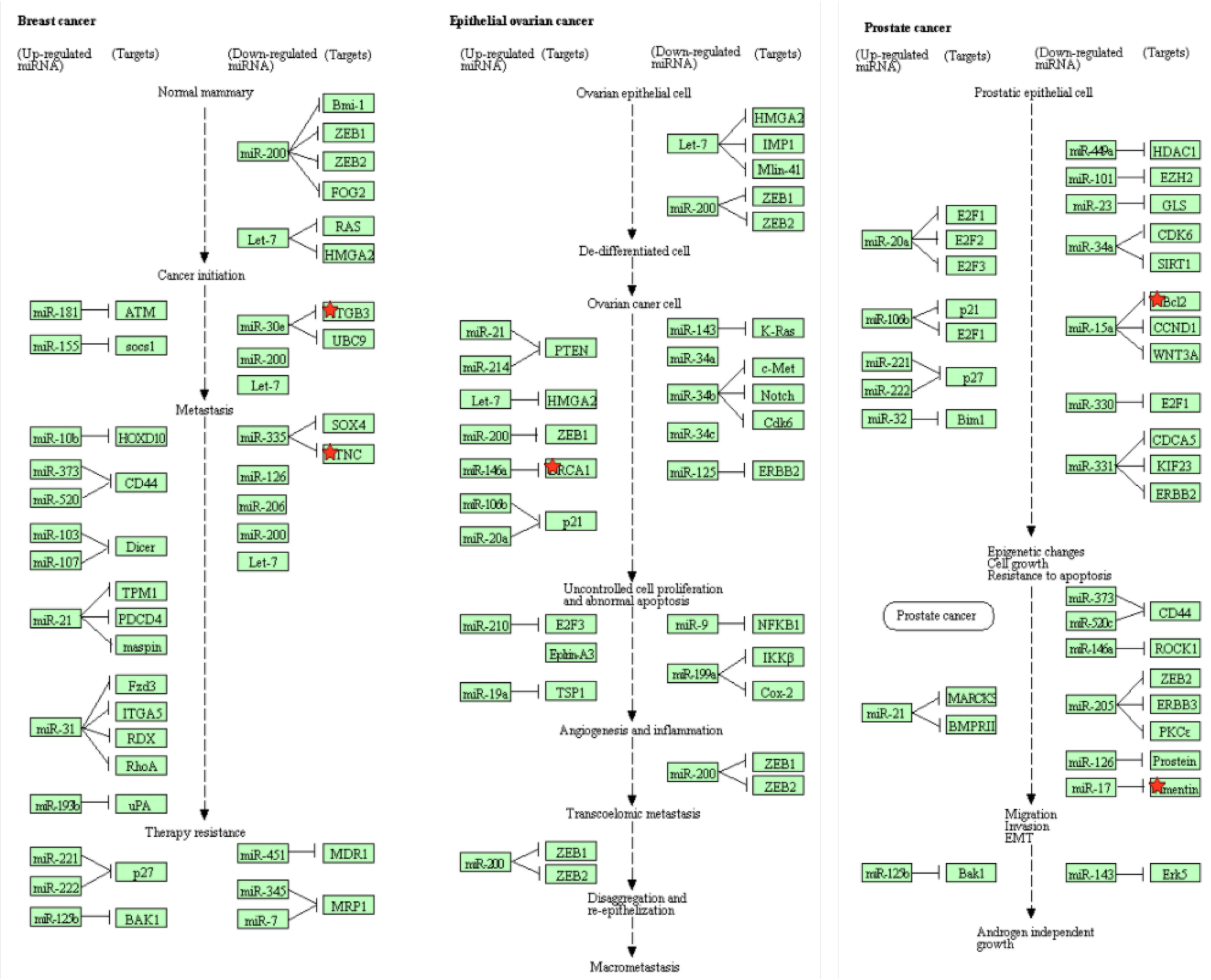

eSF$_{normal}$

P_4 induced differentially methylated genes

ERBB signaling pathway

★ Genes differentially methylated

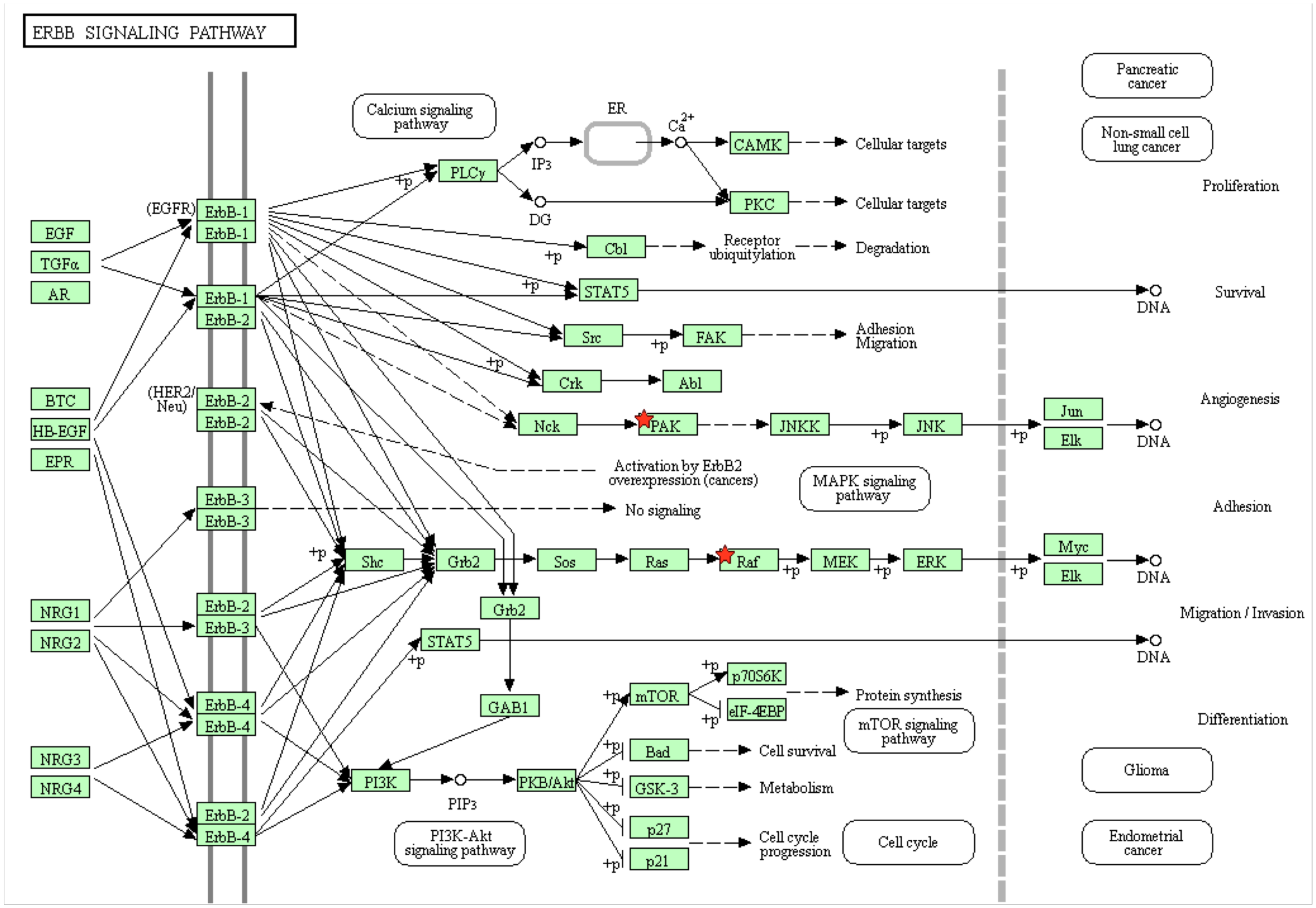

eSF$_{normal}$

E_2+ P_4 induced differentially methylated genes

Vascular smooth muscle contraction

★ Genes differentially methylated

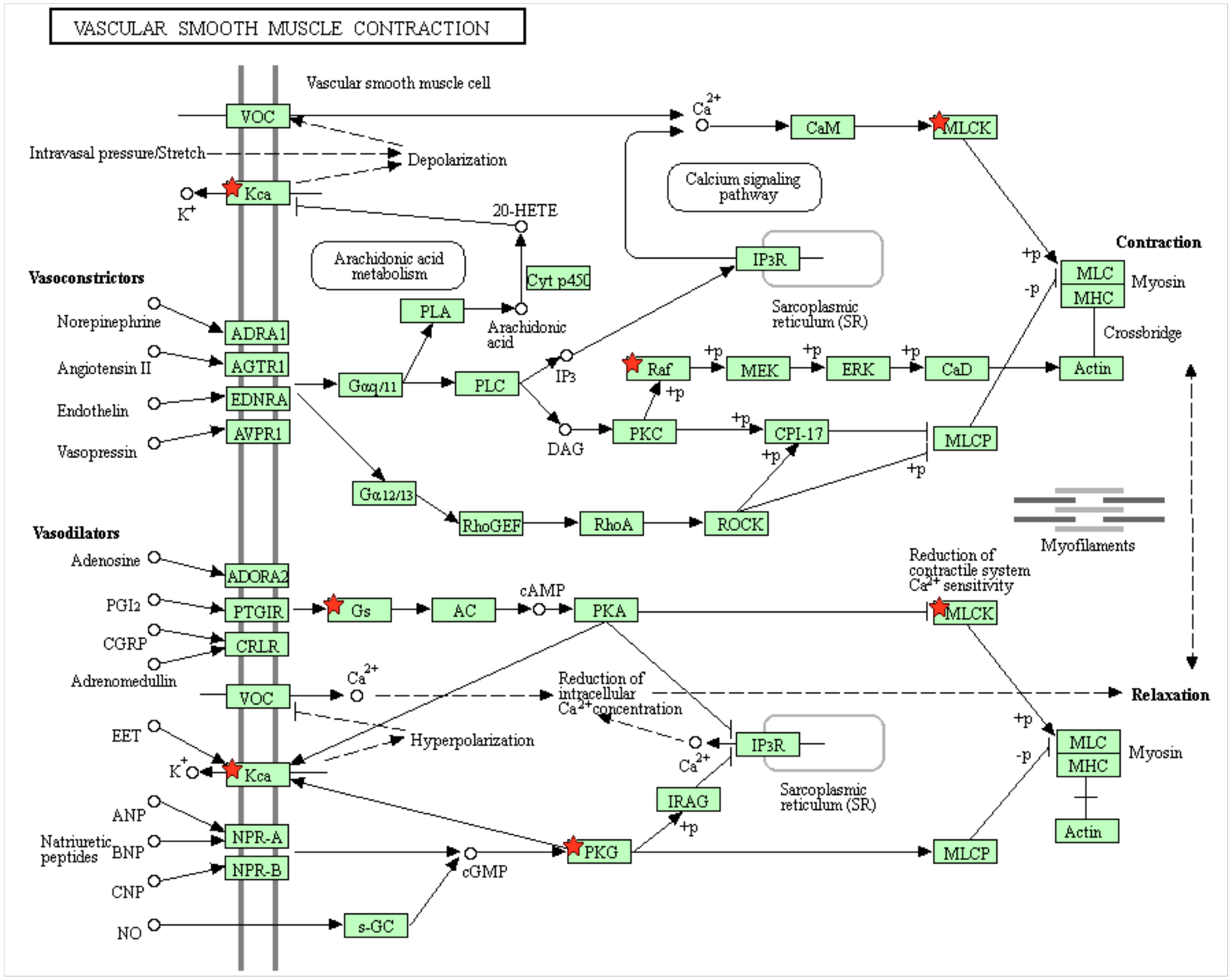

eSF$_{normal}$

E_2+ P_4 induced differentially methylated genes

Regulation of Actin Cytoskeleton

★ Genes differentially methylated

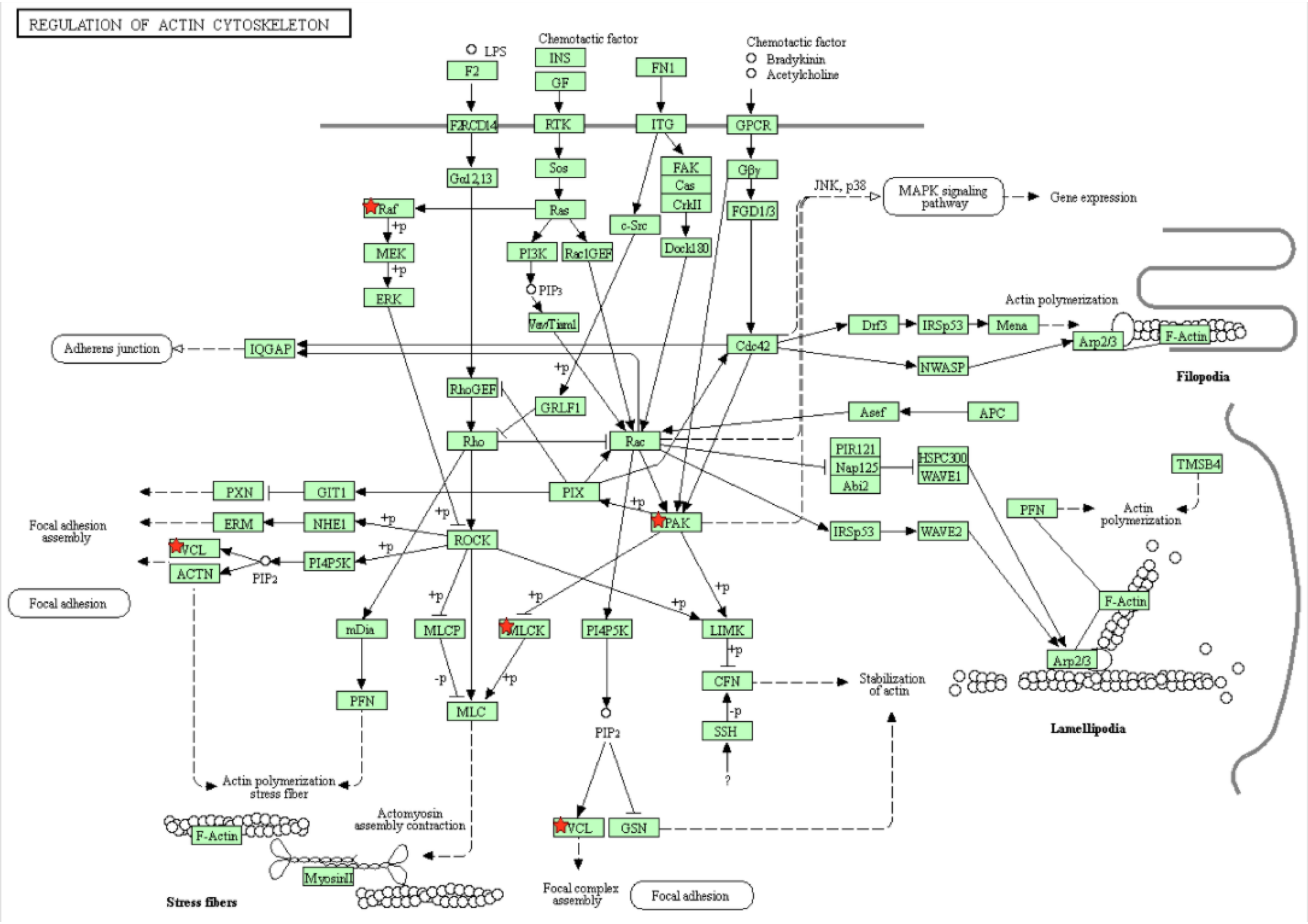

eSF_{normal}

E_2+ P_4 induced differentially methylated genes

Endometrial cancer

★ Genes differentially methylated

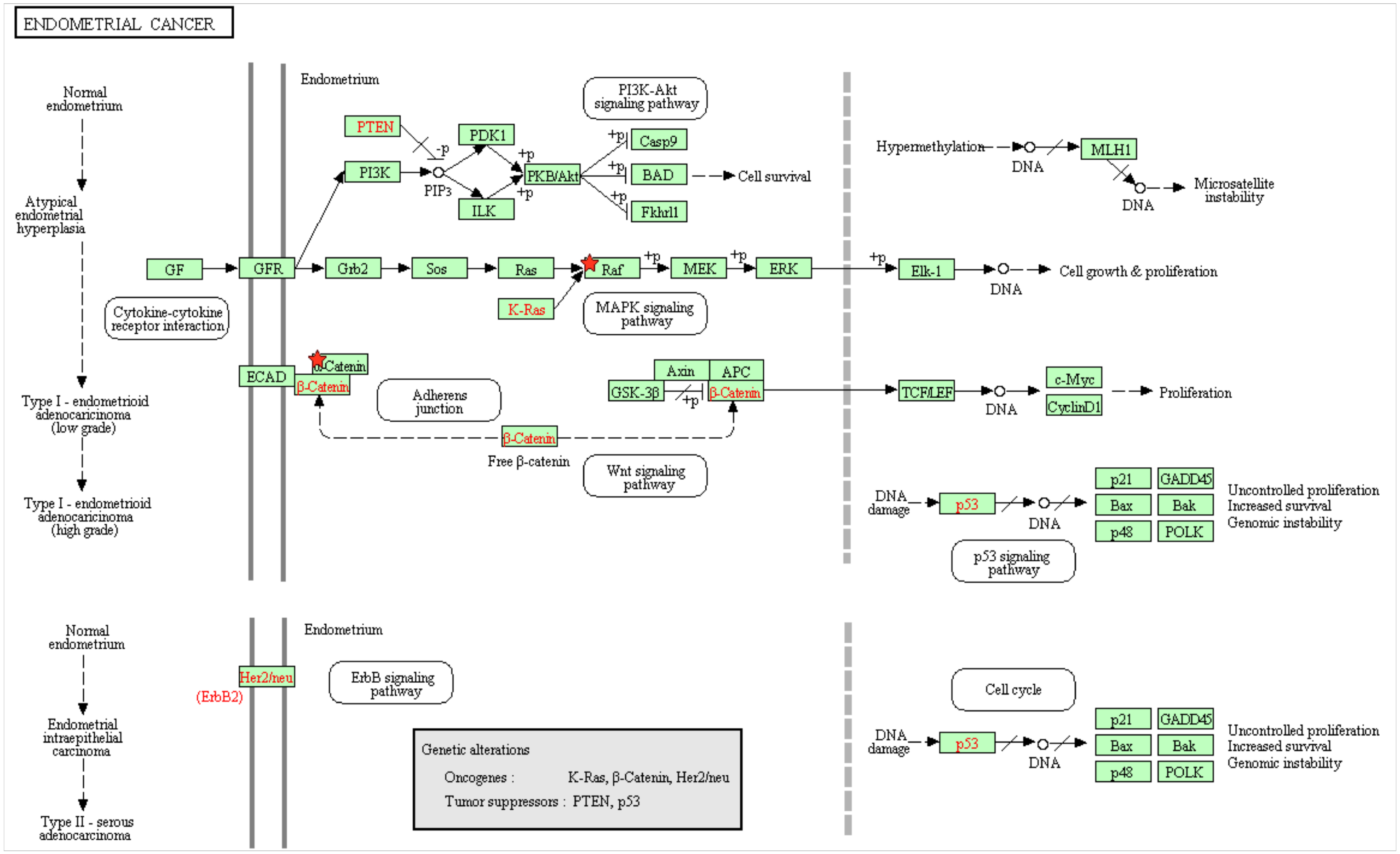

eSF$_{normal}$

E_2+ P_4 induced differentially methylated genes

NF-kB signaling pathway

★ Genes differentially methylated

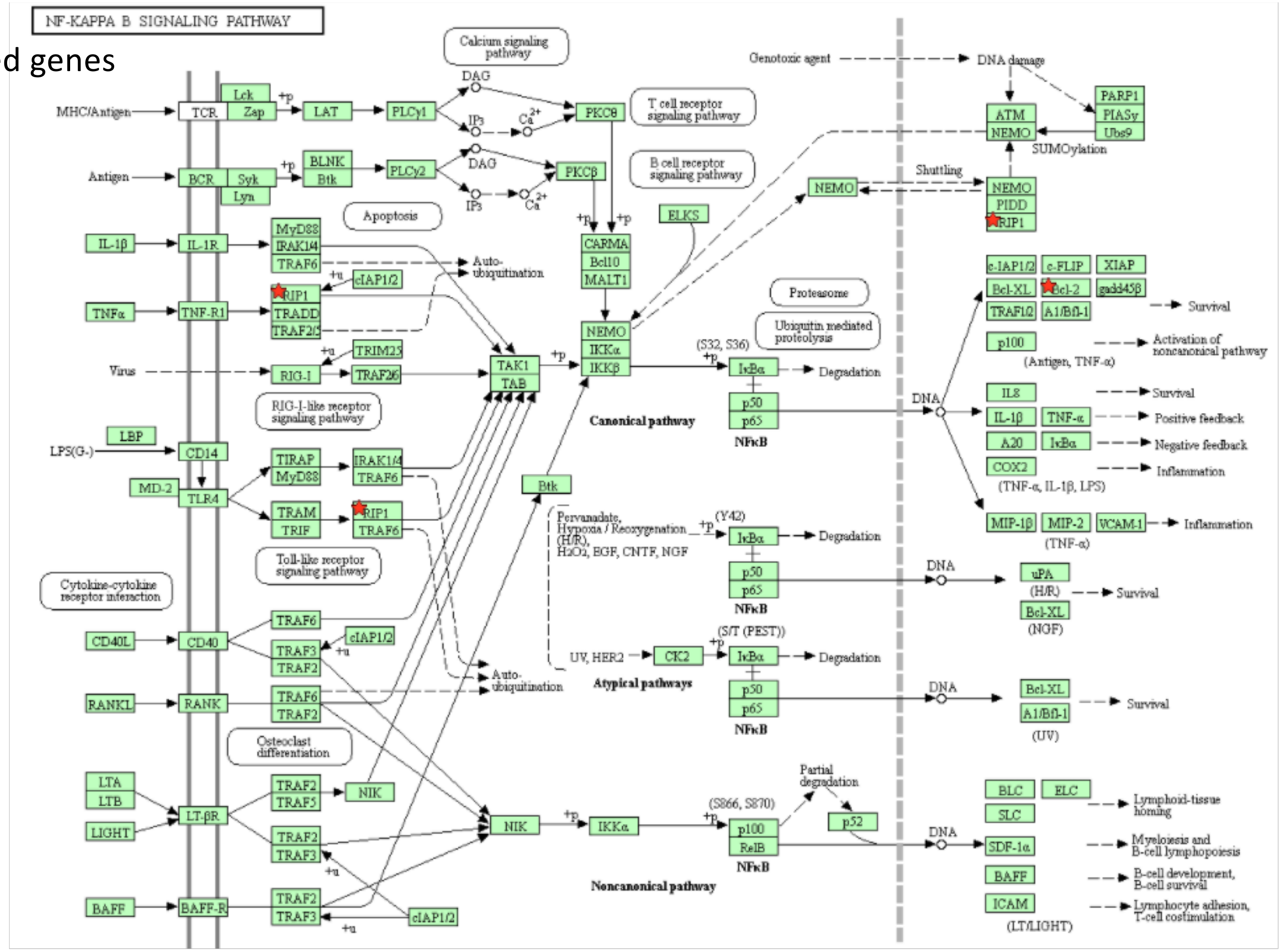

eSF_{normal}

E_2+ P_4 induced differentially methylated genes

PI3K-AKT signaling pathway

★ Genes differentially methylated

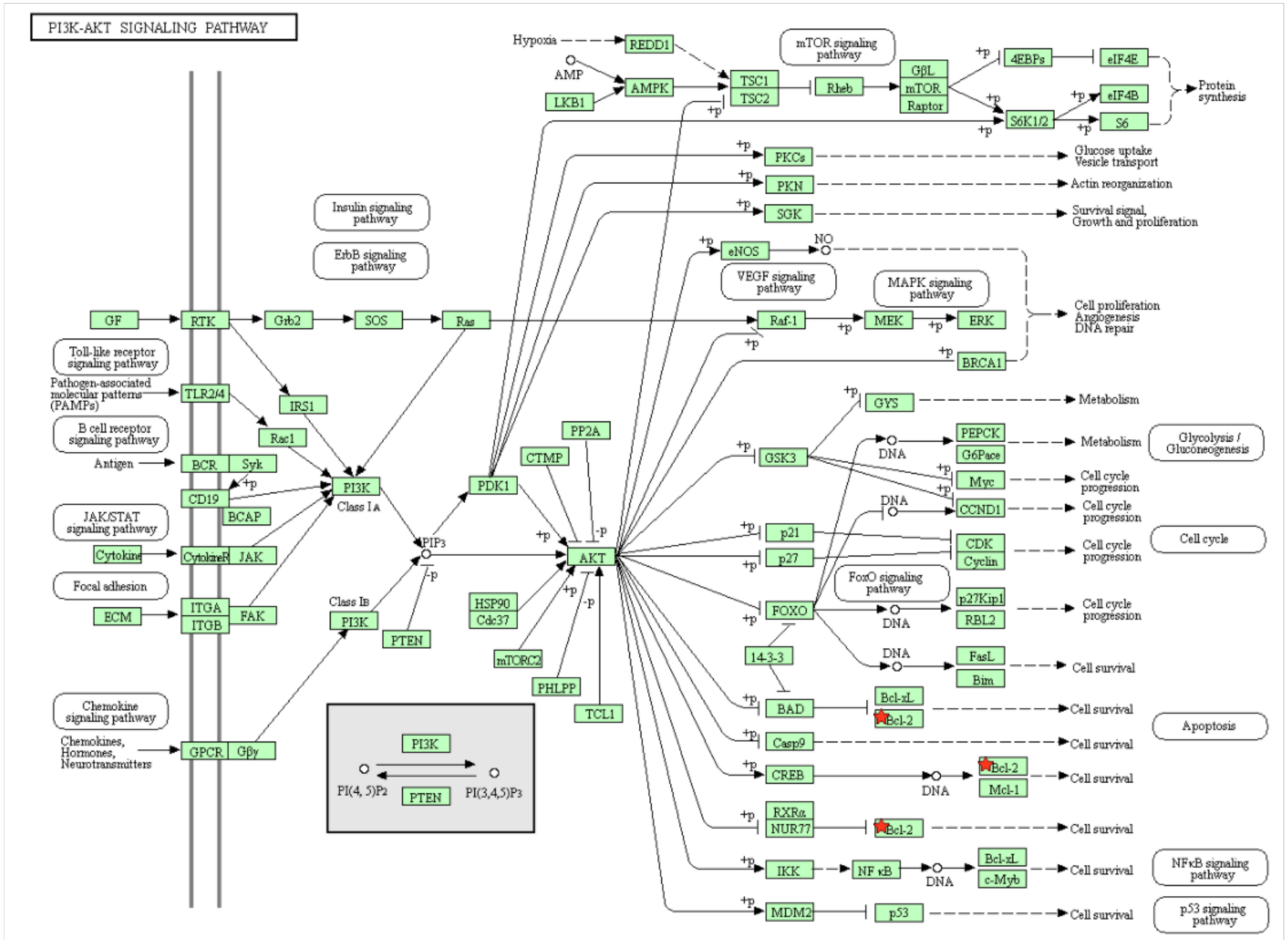

eSF_{normal}

E_2+ P_4 induced differentially methylated genes

Apoptosis

★ Genes differentially methylated

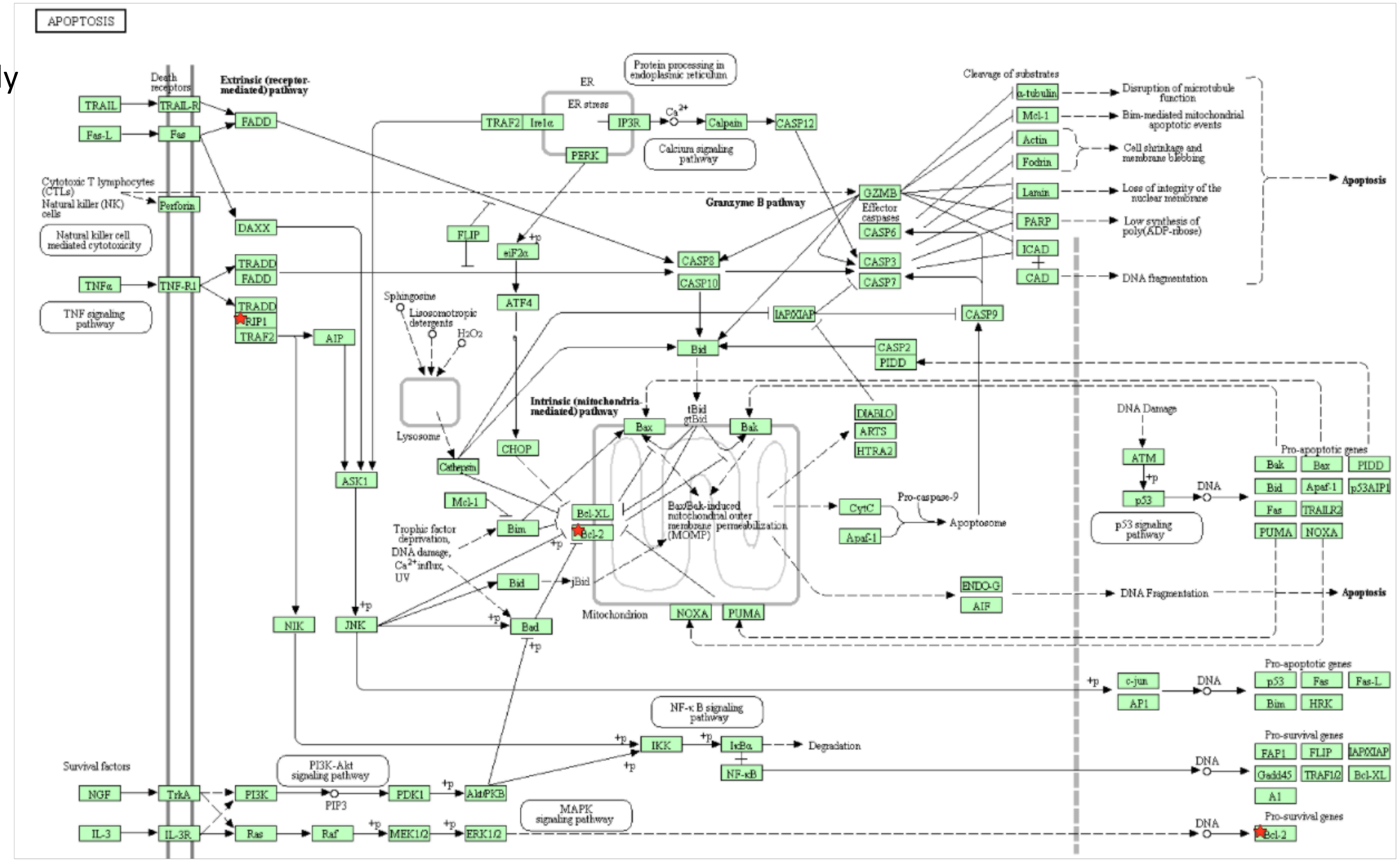

eSF$_{normal}$

E$_2$+ P$_4$ induced differentially methylated genes

MAPK signaling pathway

★ Genes differentially methylated

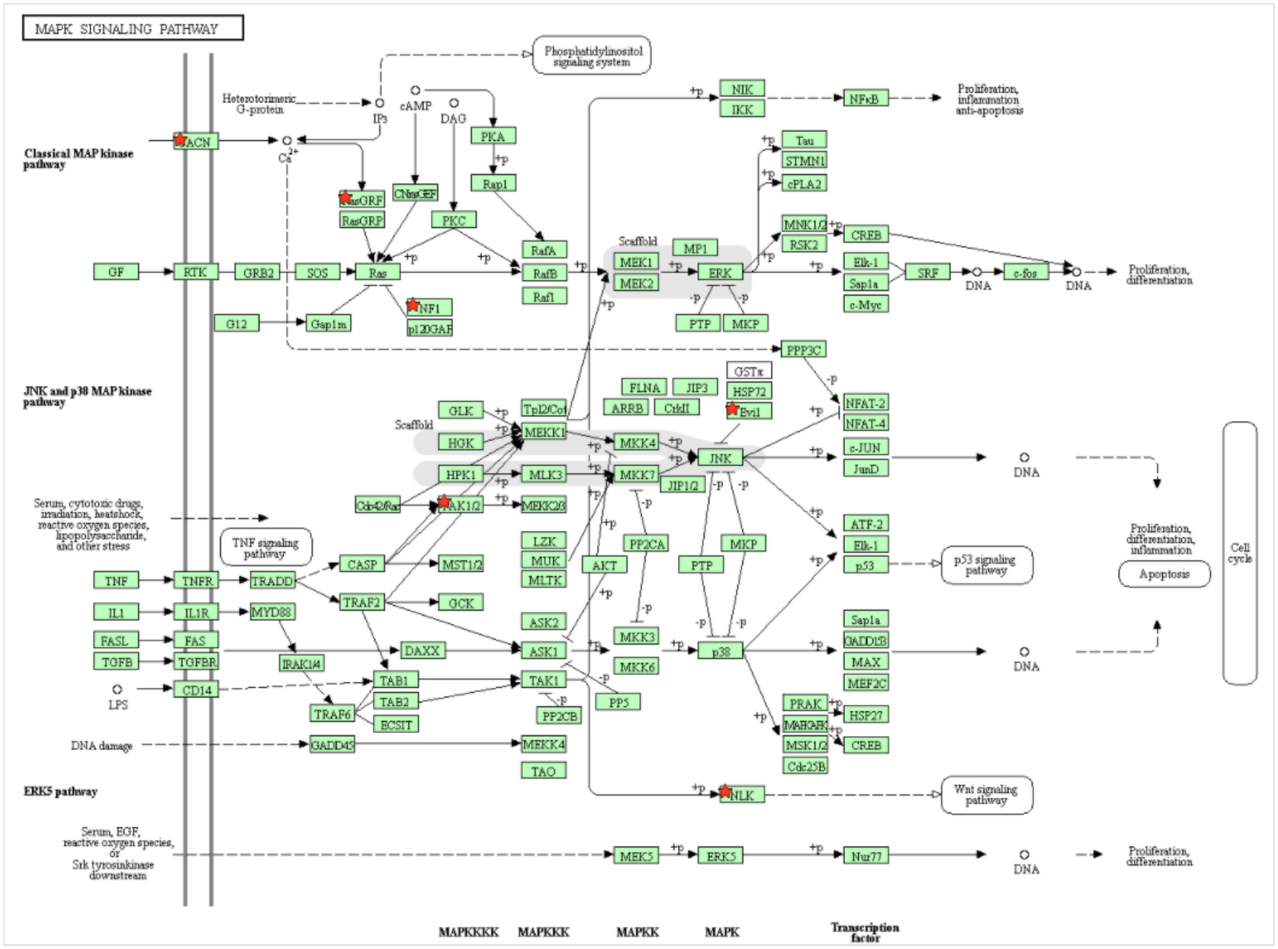

eSF$_{normal}$

E_2+ P_4 induced differentially methylated genes

RAS signaling pathway

★ Genes differentially methylated

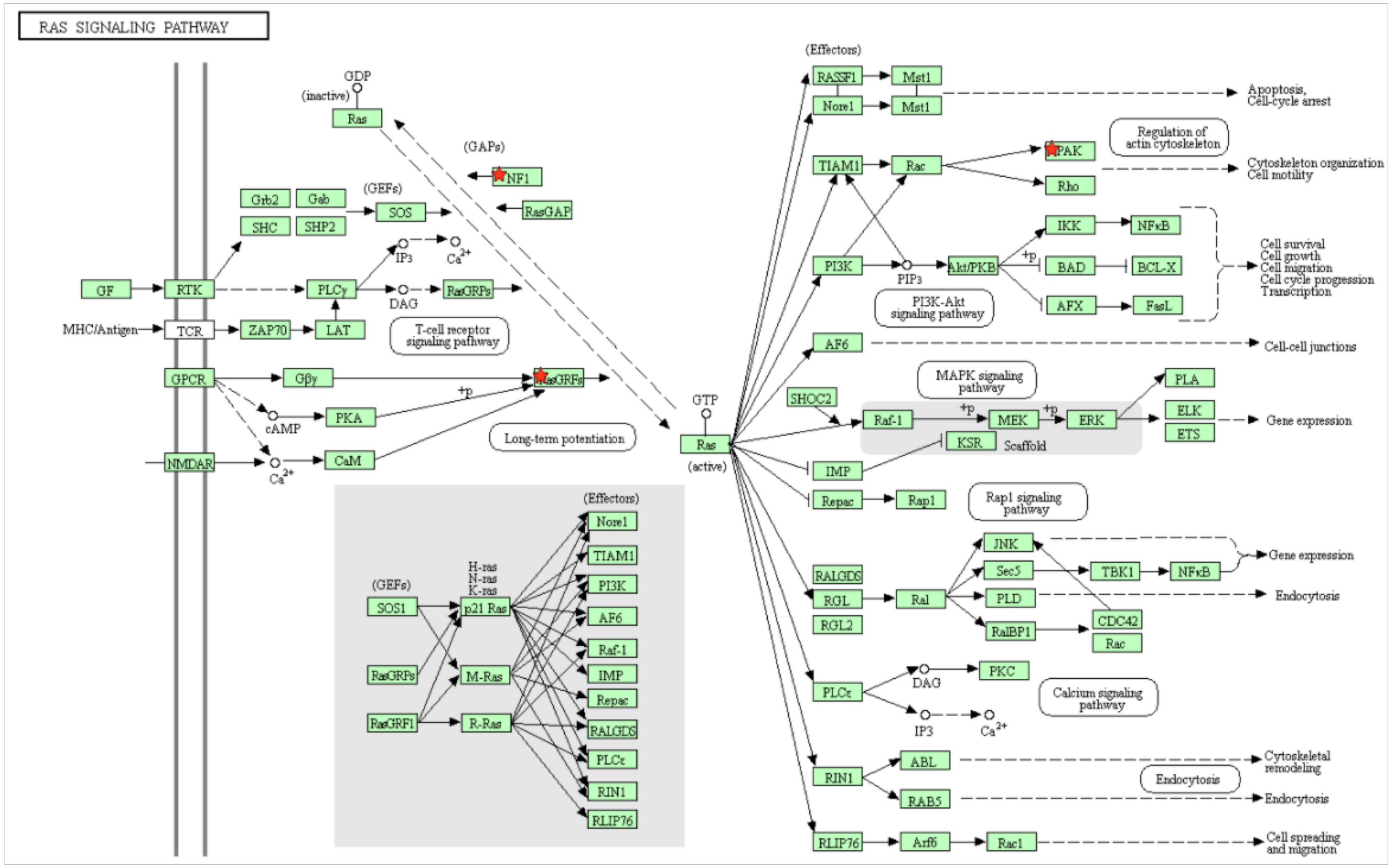

eSF$_{normal}$

E$_2$+ P$_4$ induced differentially methylated genes

Steroid hormone biosynthesis

★ Genes differentially methylated

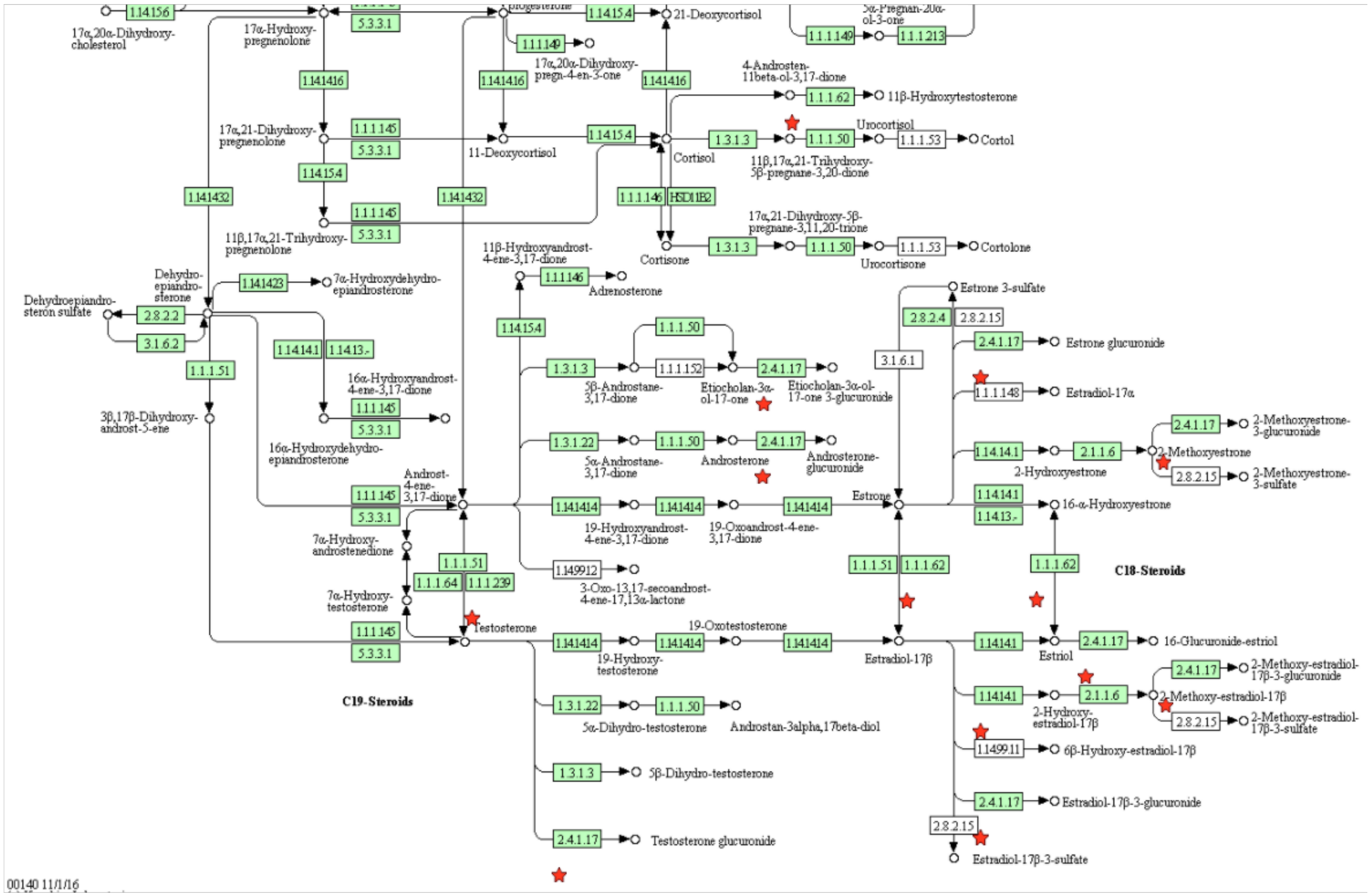

eSF$_{normal}$

E_2+ P_4 induced differentially methylated genes

Cell adhesion

★ Genes differentially methylated

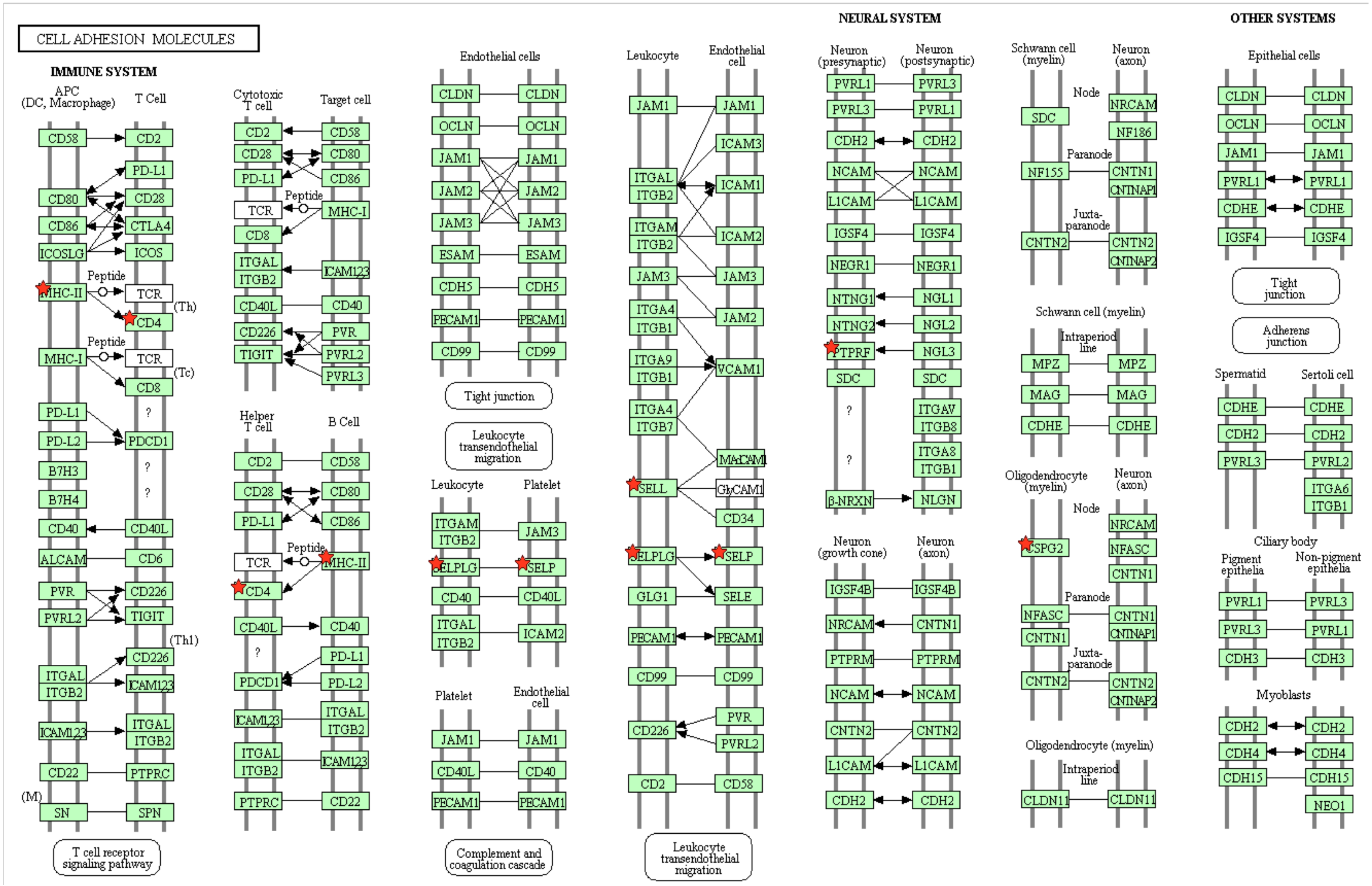

$eSF_{stage\ I}$

E_2 induced differentially methylated genes

Proteoglycans in cancer

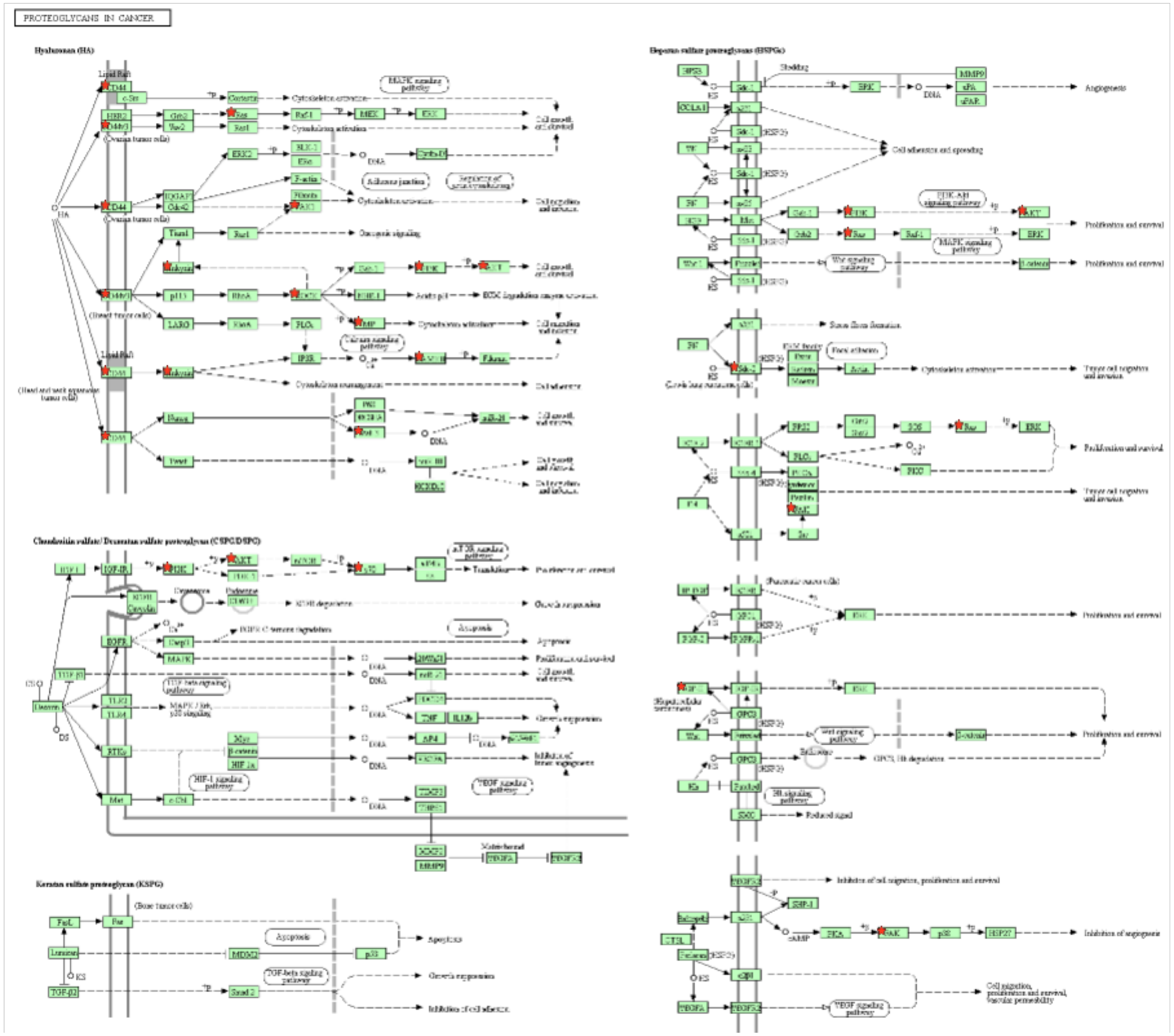

★ Genes differentially methylated

$eSF_{stage\ I}$

E_2 induced differentially methylated genes

Tight Junction

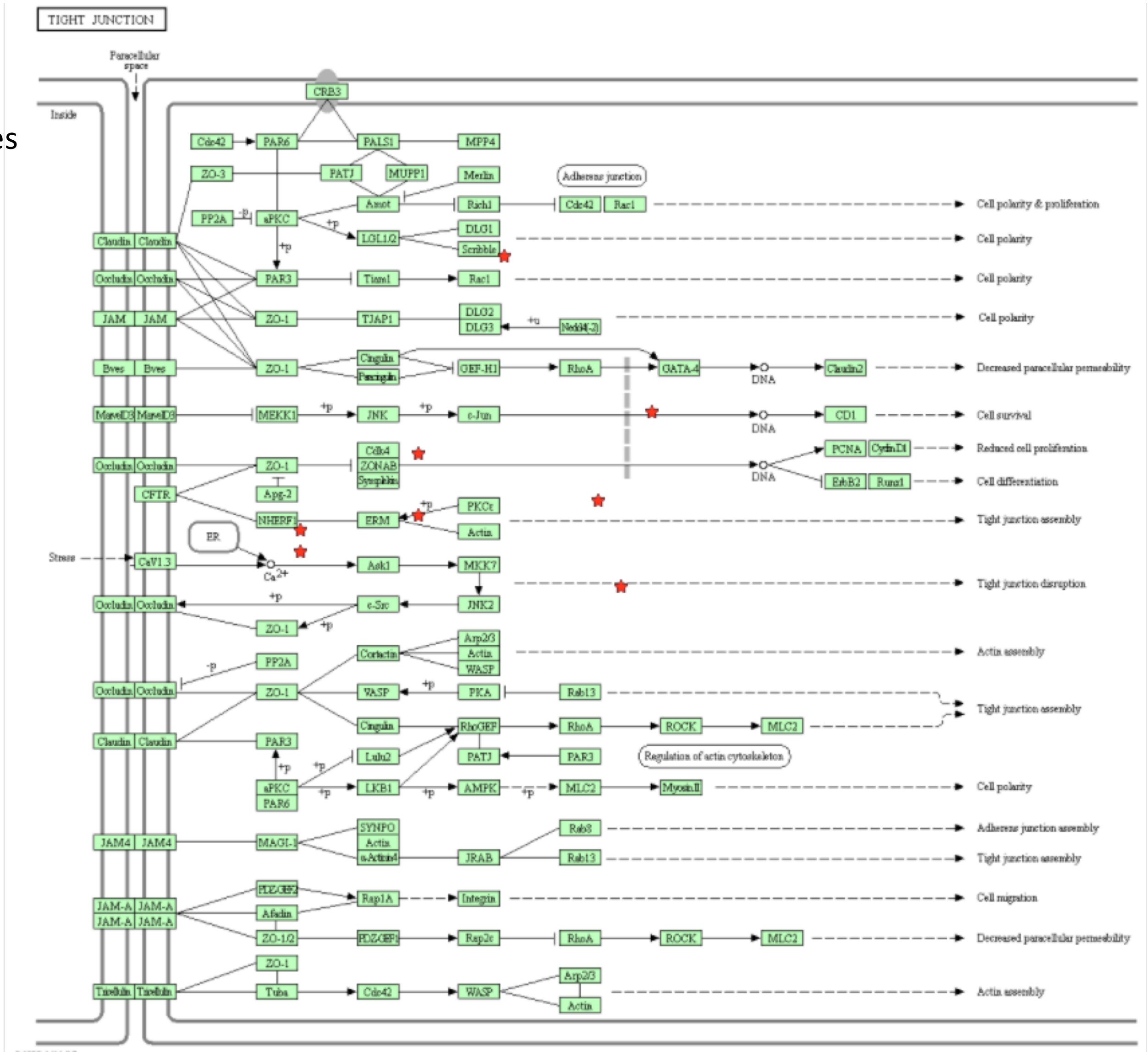

★ Genes differentially methylated

eSF$_{stage\ I}$

E$_2$ induced differentially methylated genes

PI3K-AKT signaling pathway

★ Genes differentially methylated

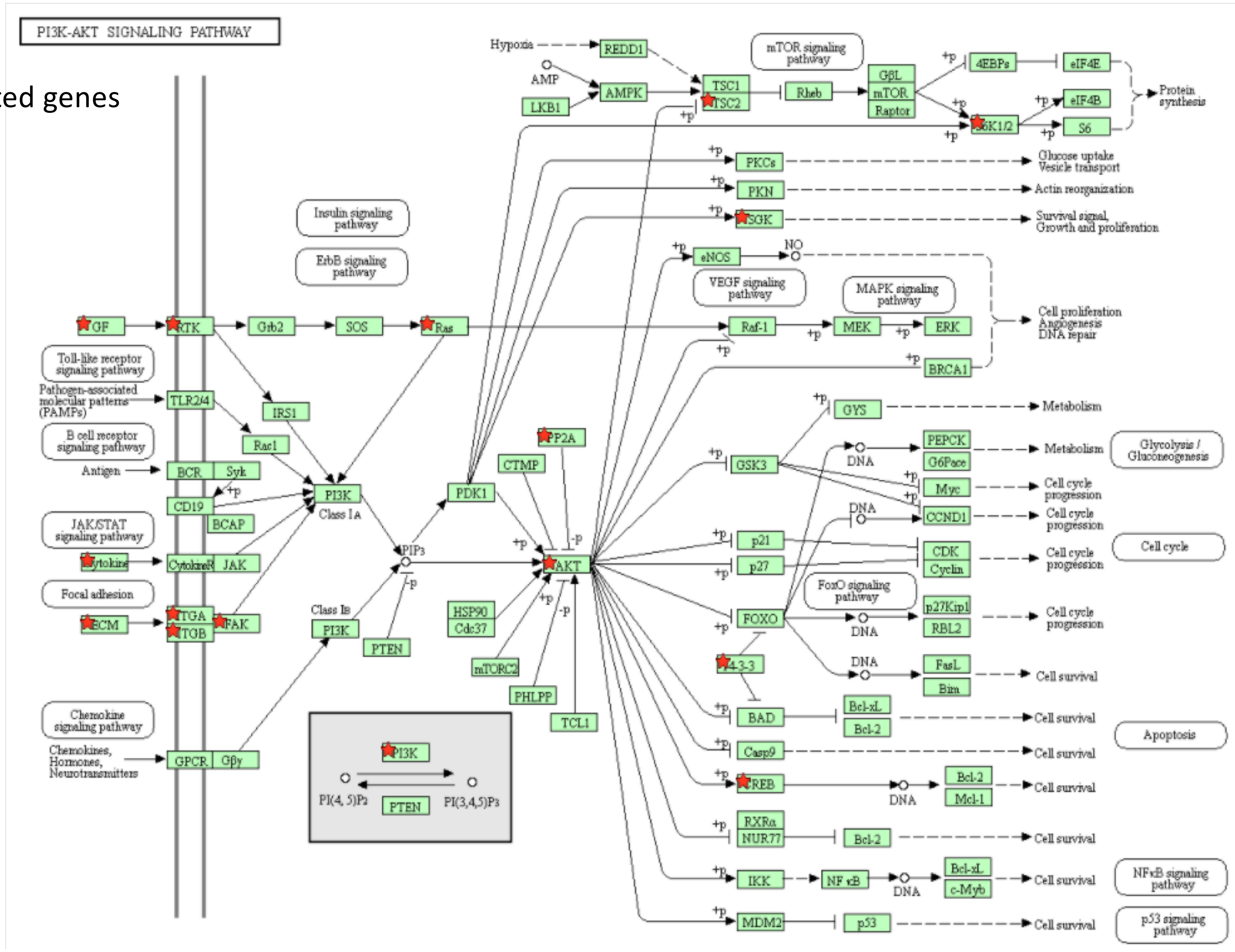

$eSF_{stage\ I}$

E_2 induced differentially methylated genes

Cytokine-Chemokine interaction

★ Genes differentially methylated

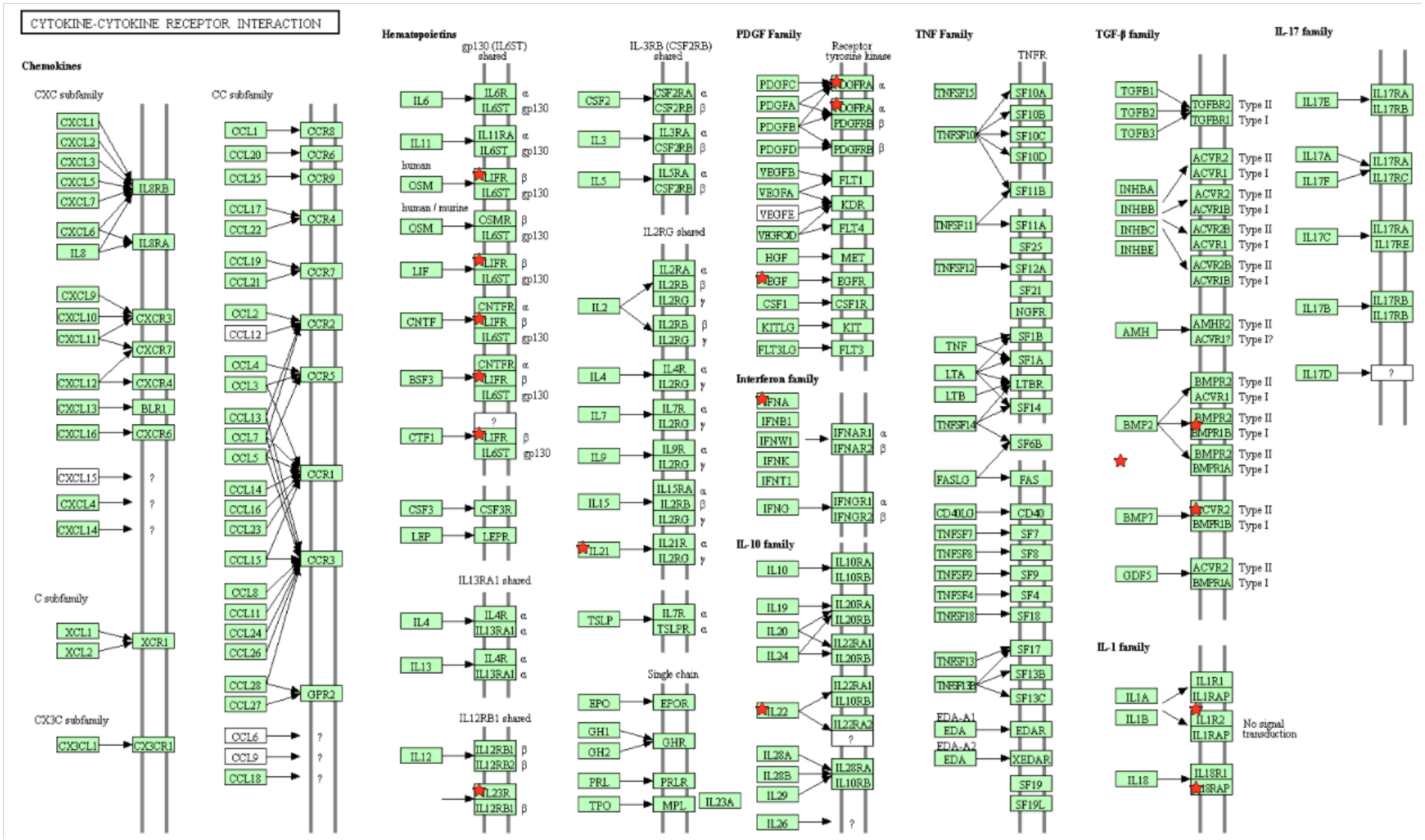

eSF$_{stage\ I}$

E_2 induced differentially methylated genes

RAS signaling pathway

★ Genes differentially methylated

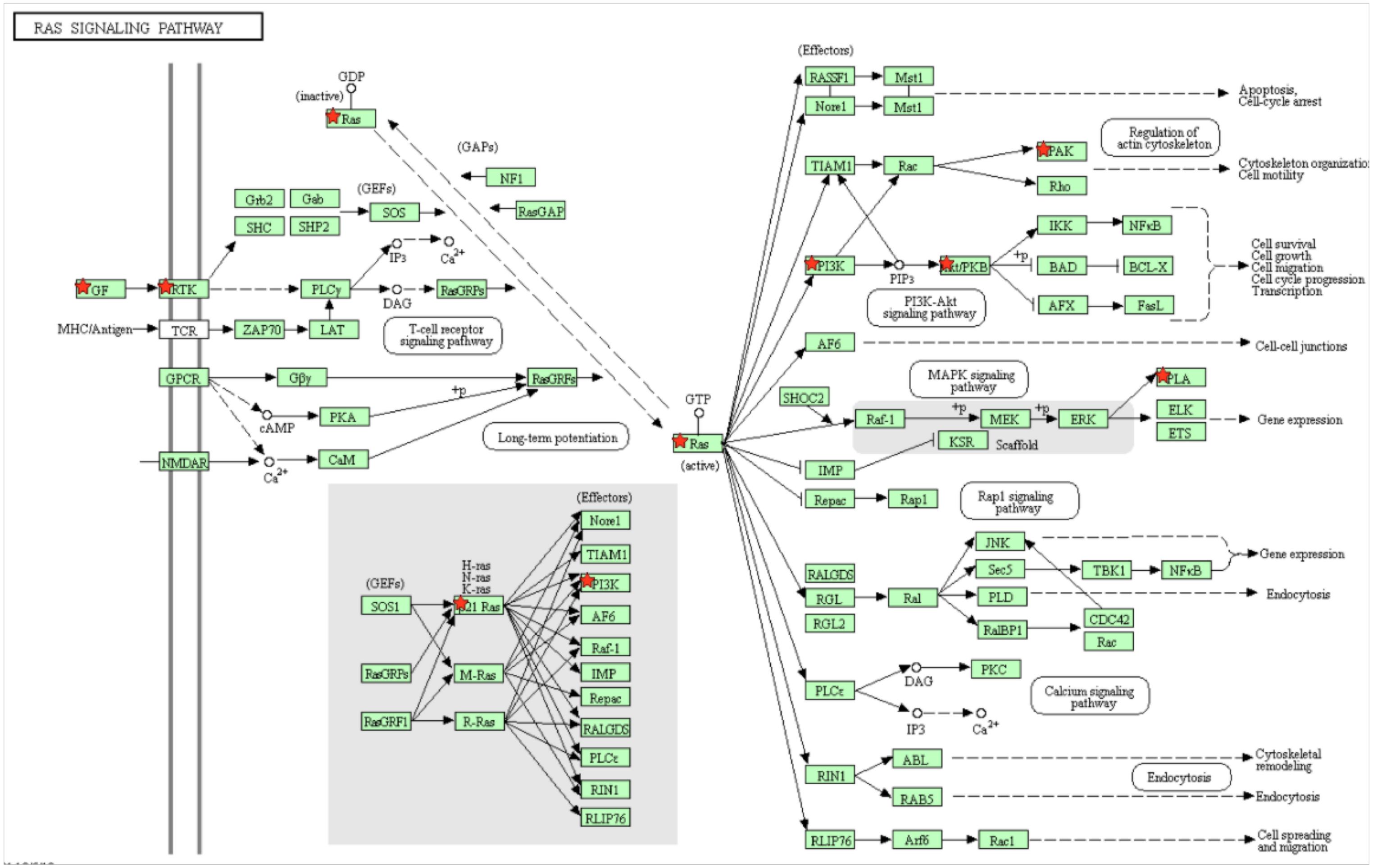

eSF$_{stage\ I}$

E_2 induced differentially methylated genes

Calcium signaling pathway

★ Genes differentially methylated

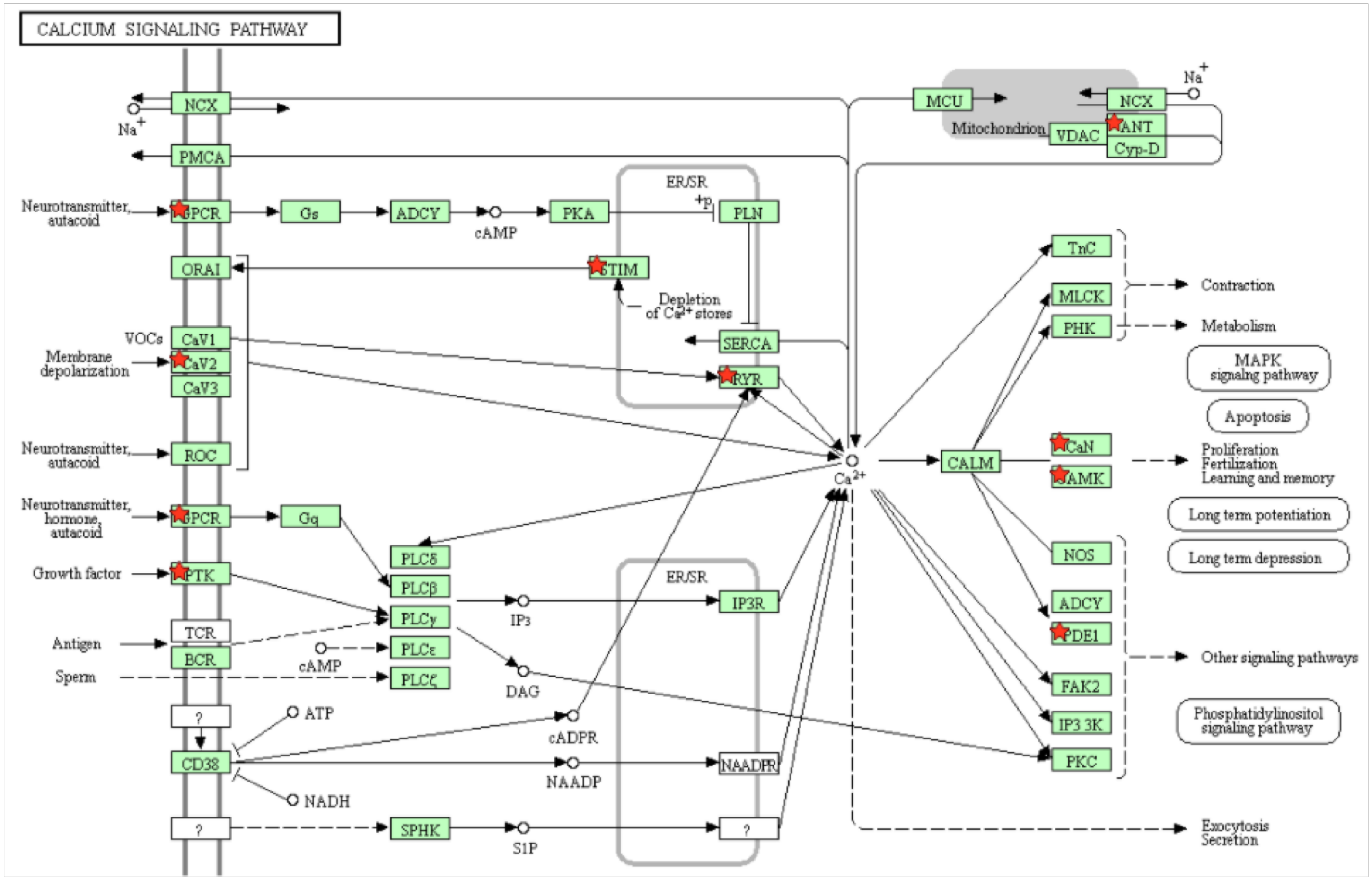

eSF$_{\textbf{stage I}}$

E$_2$ induced differentially methylated genes

GAP Junction

★ Genes differentially methylated

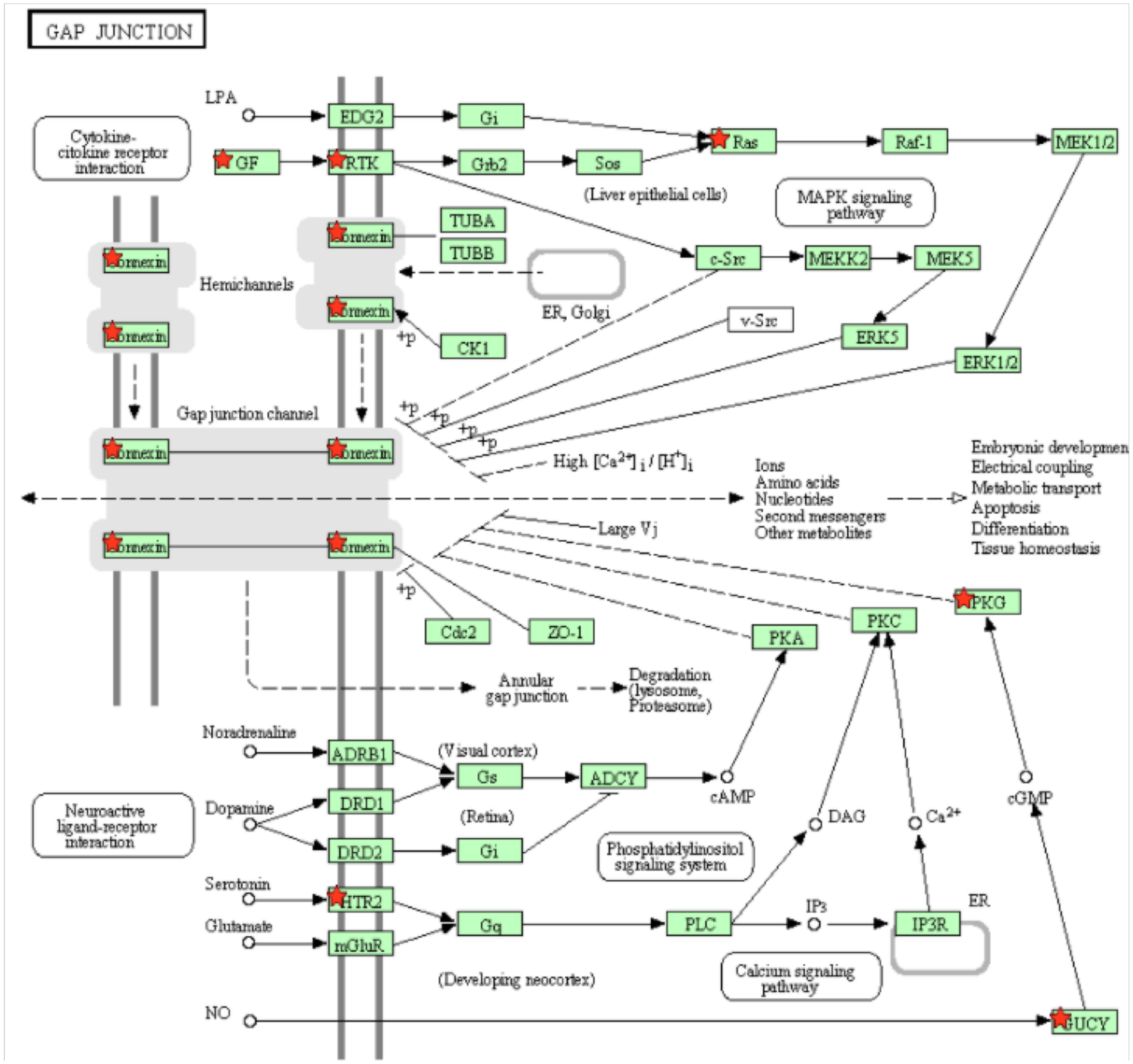

$eSF_{stage\ I}$

E_2 induced differentially methylated genes

MAPK signaling pathway

★ Genes differentially methylated

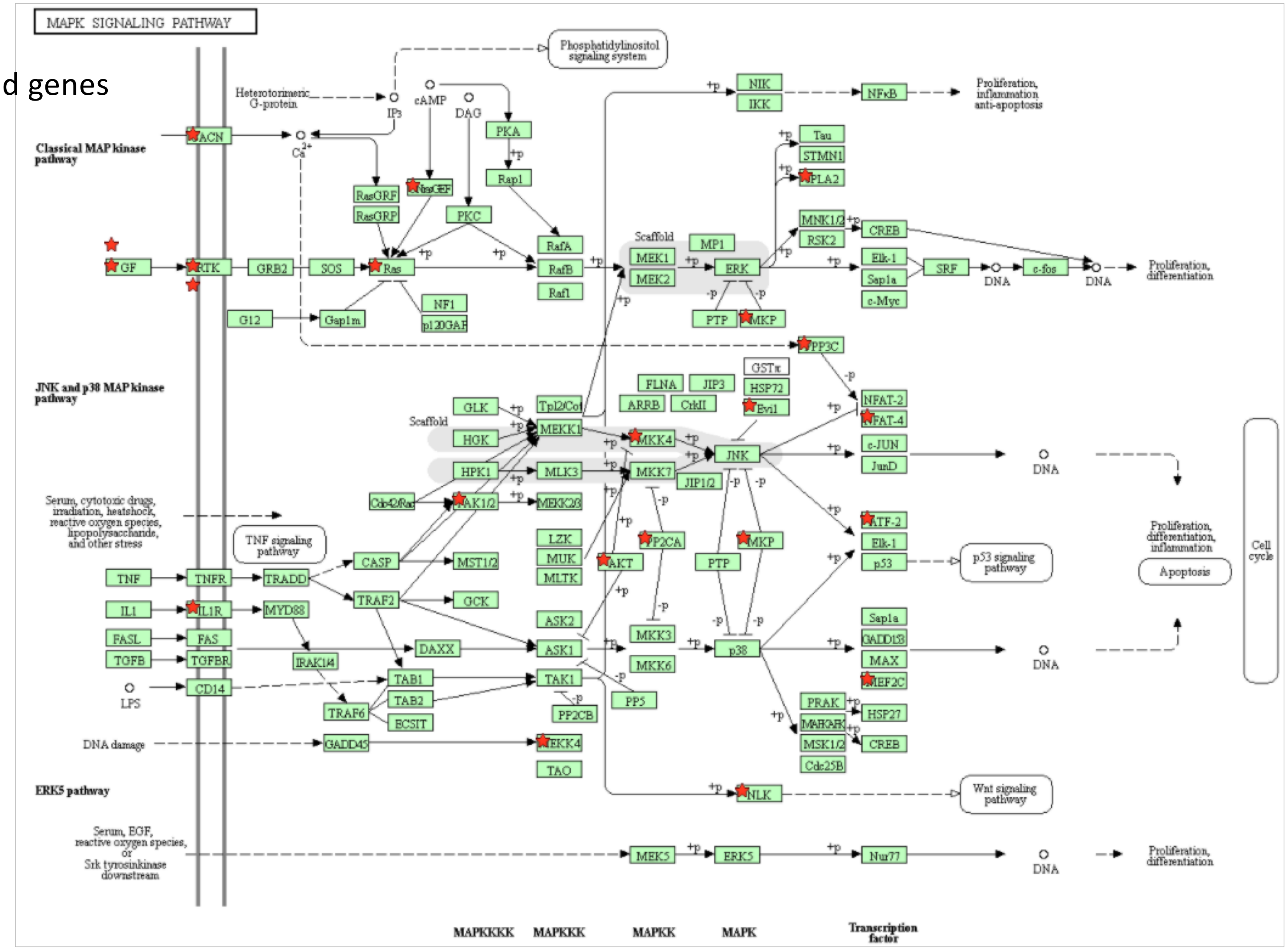

eSF$_{\text{stage I}}$

E$_2$ induced differentially methylated genes

ERBB signaling pathway

★ Genes differentially methylated

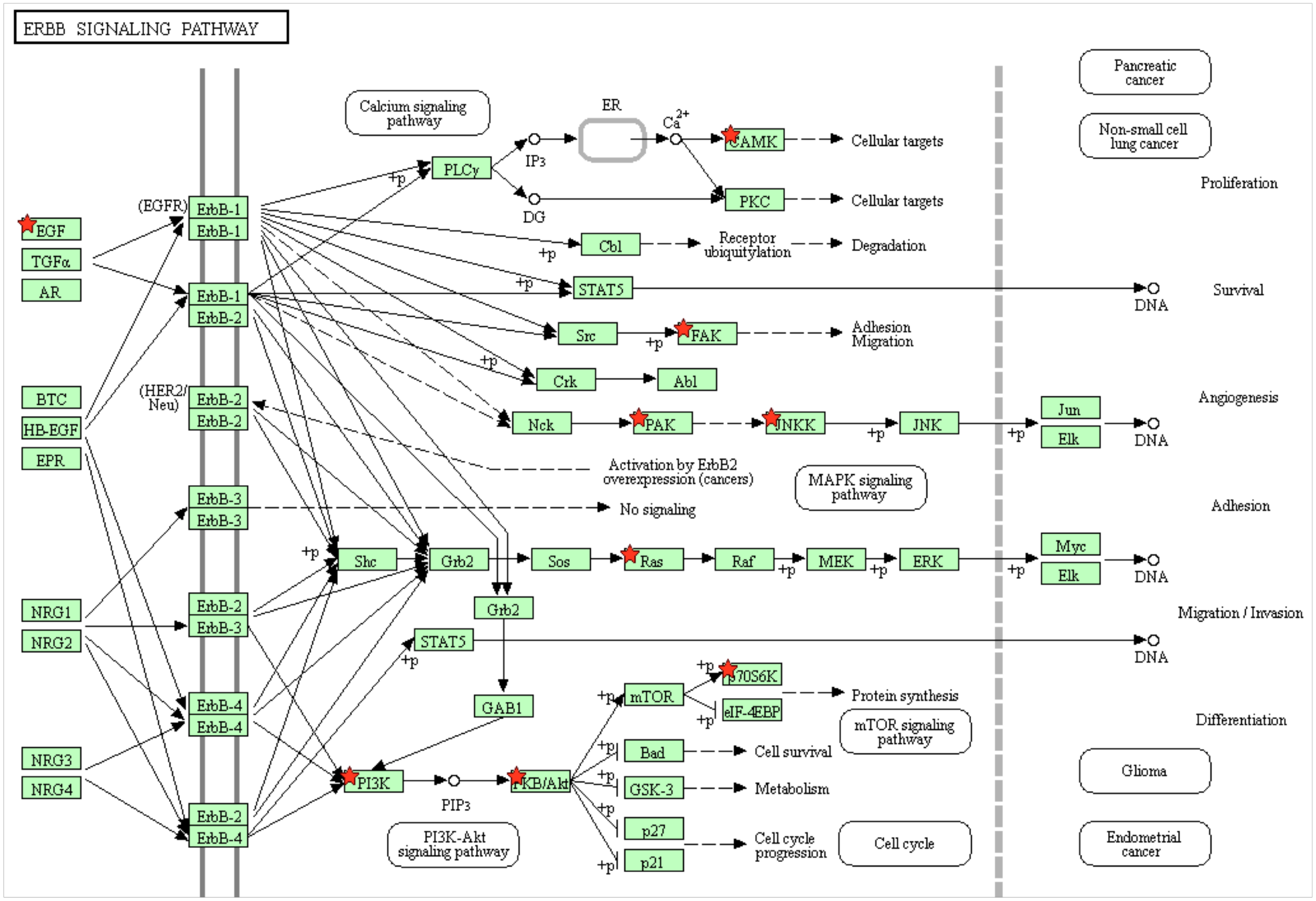

eSF$_{\text{stage I}}$

E_2 induced differentially methylated genes

RAS signaling pathway

★ Genes differentially methylated

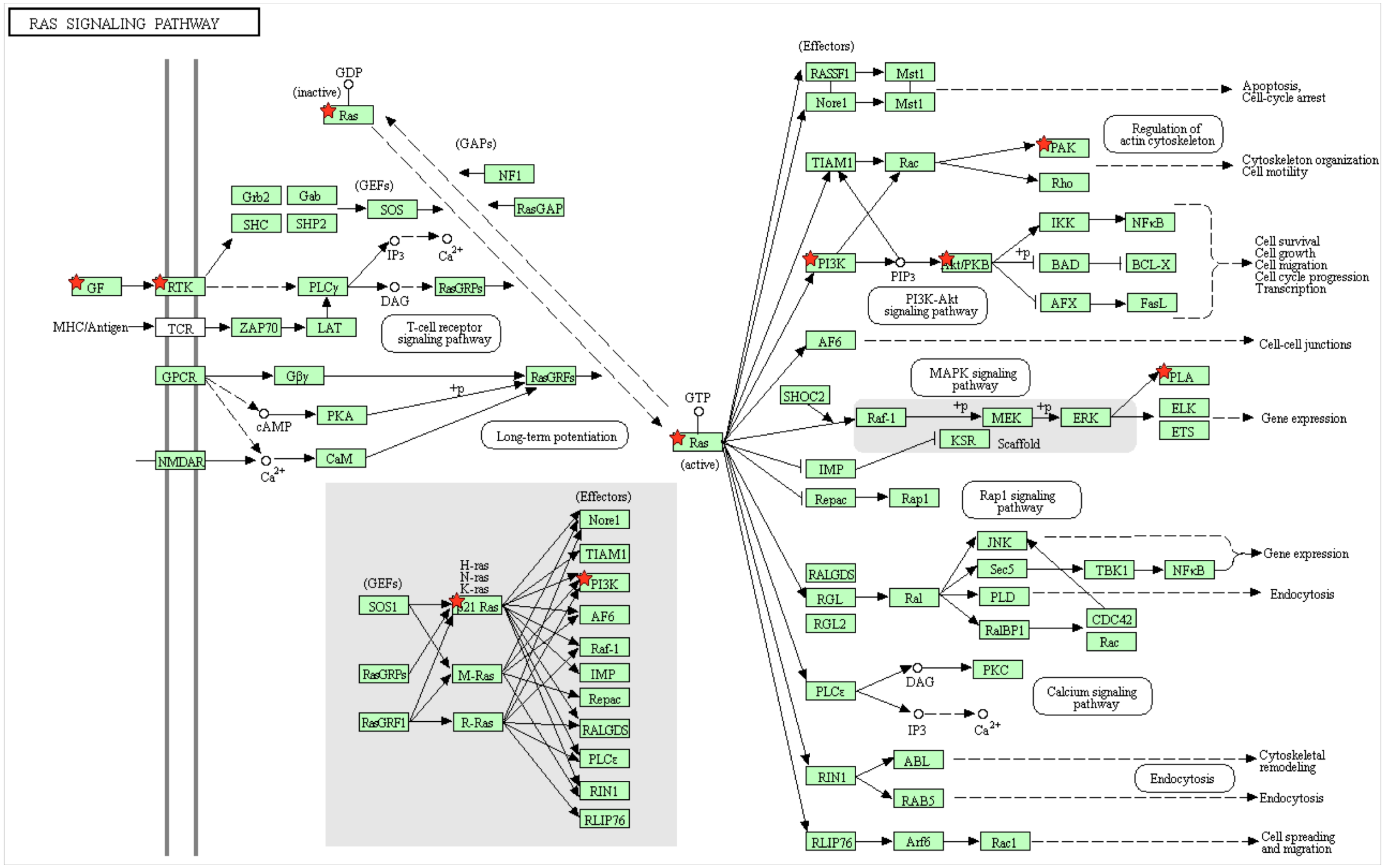

eSF$_{stage\ I}$
E_2 induced differentially methylated genes
VEGF signaling pathway

★ Genes differentially methylated

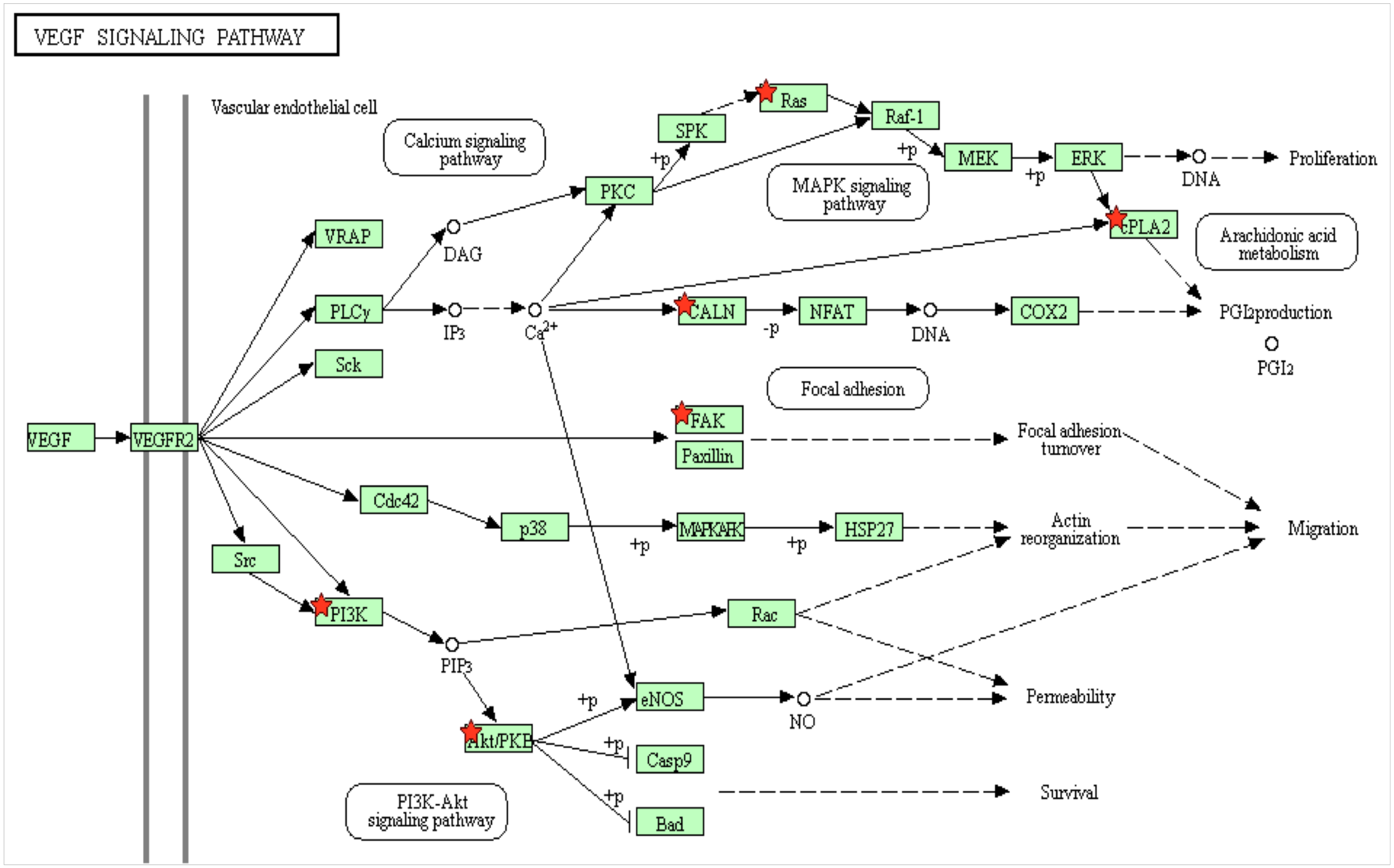

eSF$_{stage\ I}$
E_2 induced differentially methylated genes
JAK-STAT signaling pathway

★ Genes differentially methylated

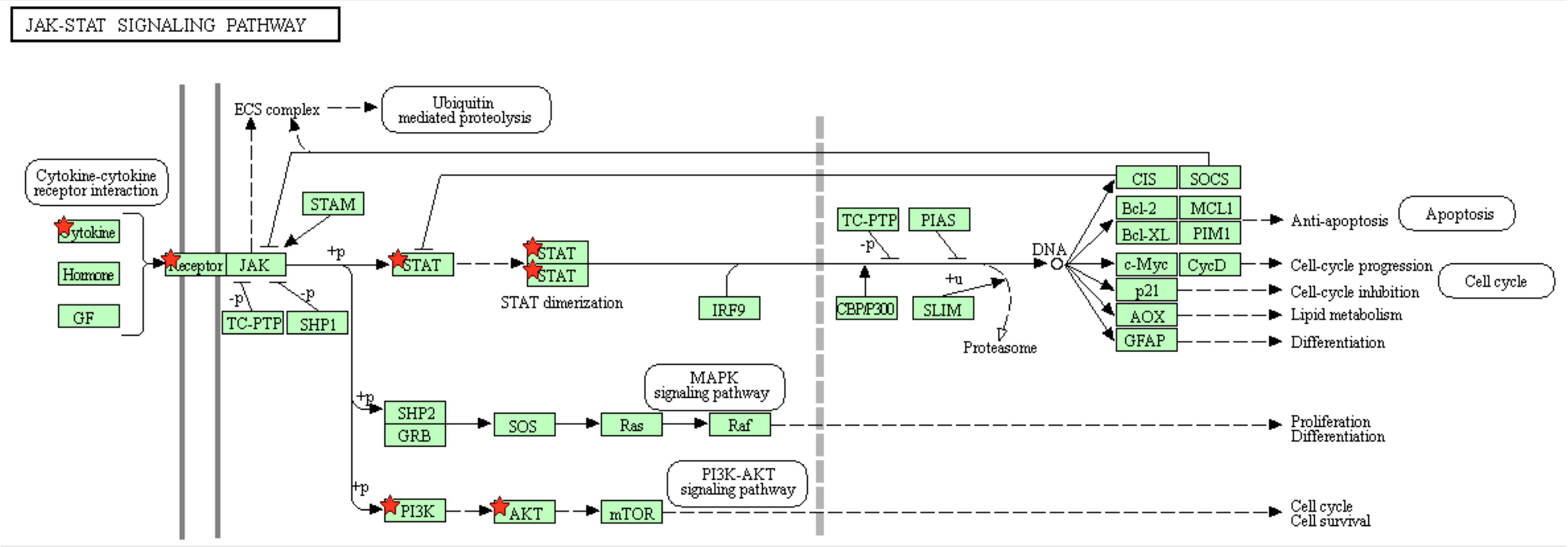

eSF$_{stage\ I}$

E$_2$ induced differentially methylated genes

P53 signaling pathway

★ Genes differentially methylated

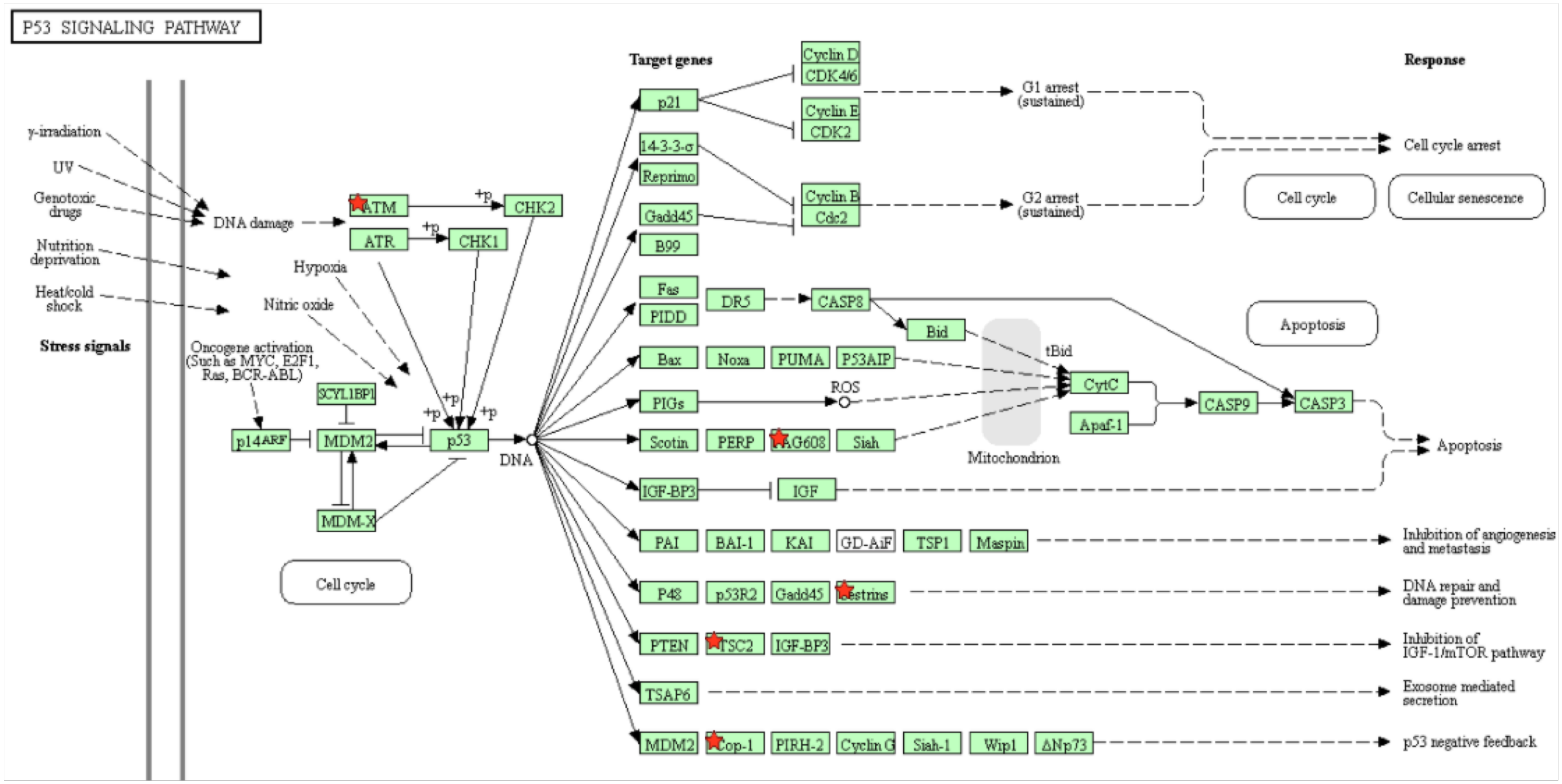

eSF$_{stage\ I}$

E$_2$ induced differentially methylated genes

Focal Adhesion

★ Genes differentially methylated

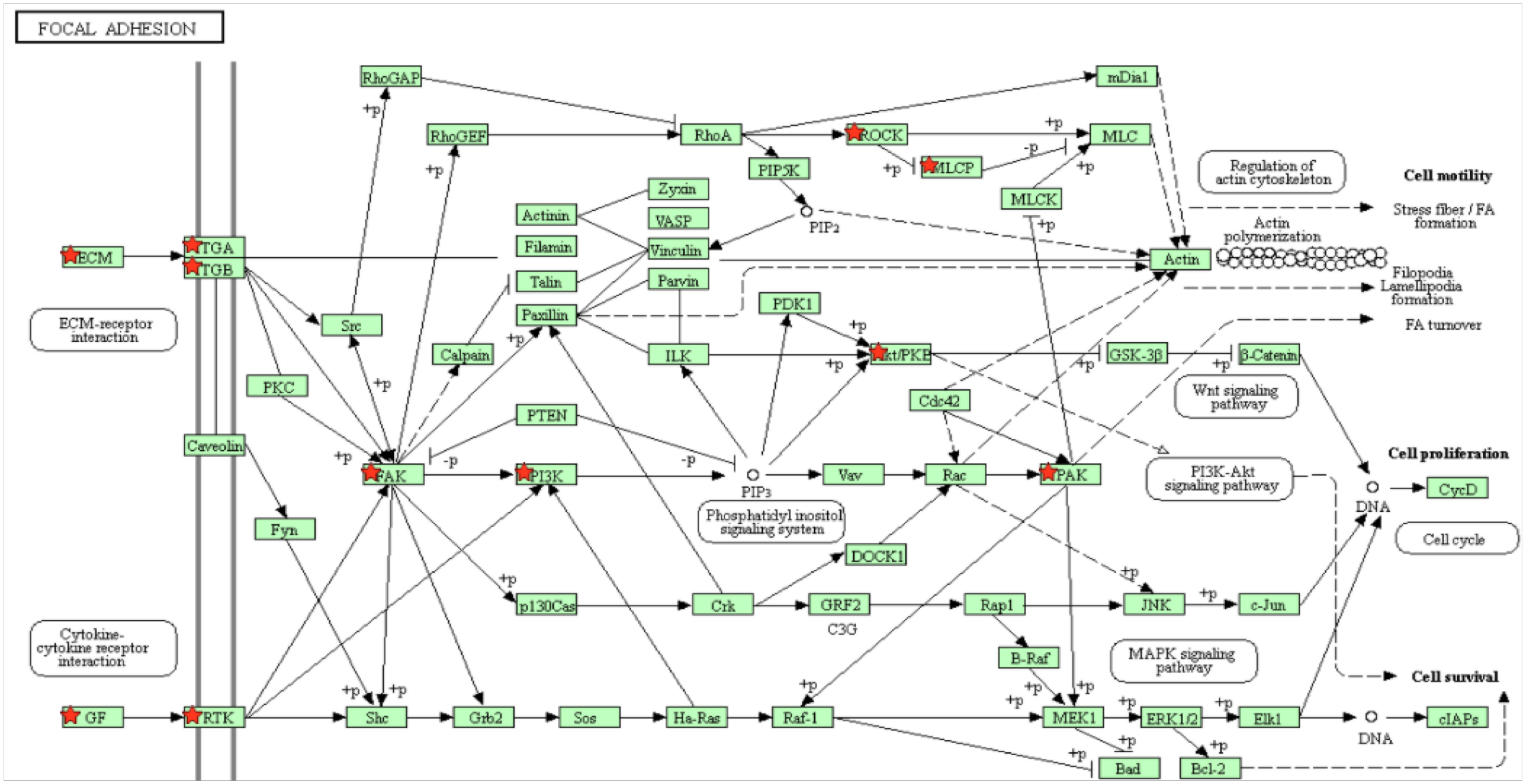

eSF$_{\text{stage I}}$

E$_2$ induced differentially methylated genes

FOXO signaling pathway

★ Genes differentially methylated

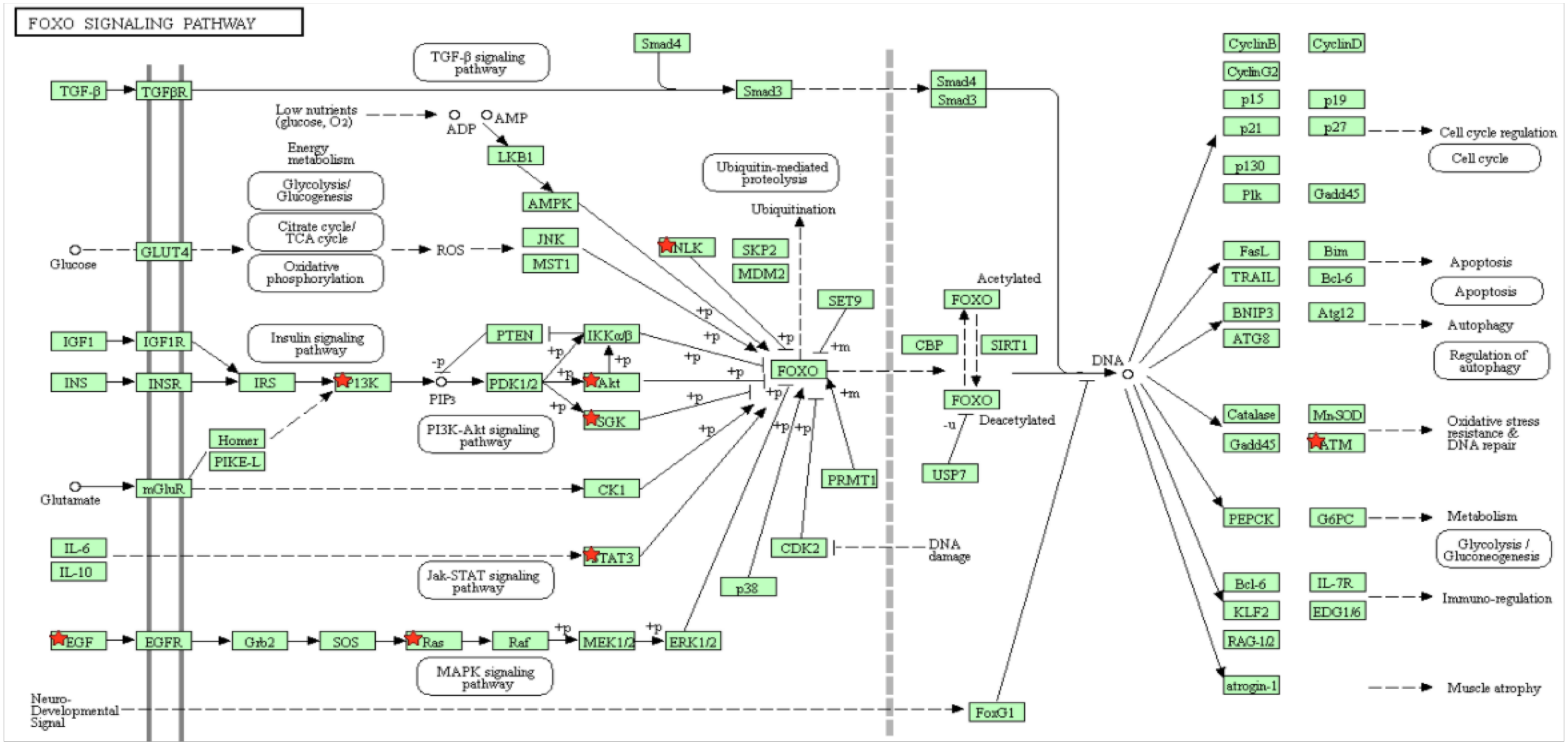

eSF$_{stage\ I}$

E_2 induced differentially methylated genes

Actin Cytoskeleton

★ Genes differentially methylated

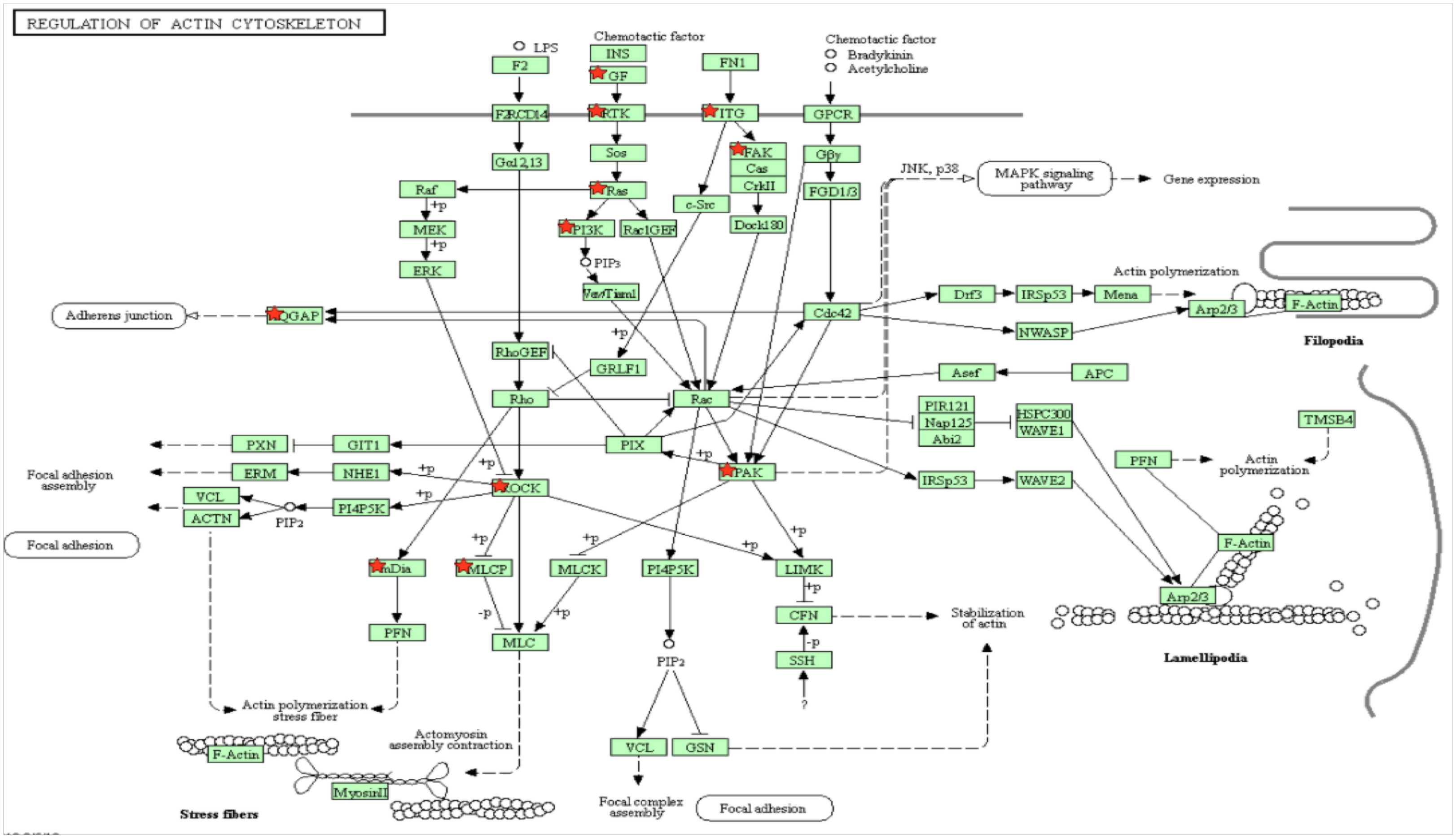

eSF$_{stage\ IV}$

E$_2$ induced differentially methylated genes

Regulation of stem cell pluripotency

★ Genes differentially methylated

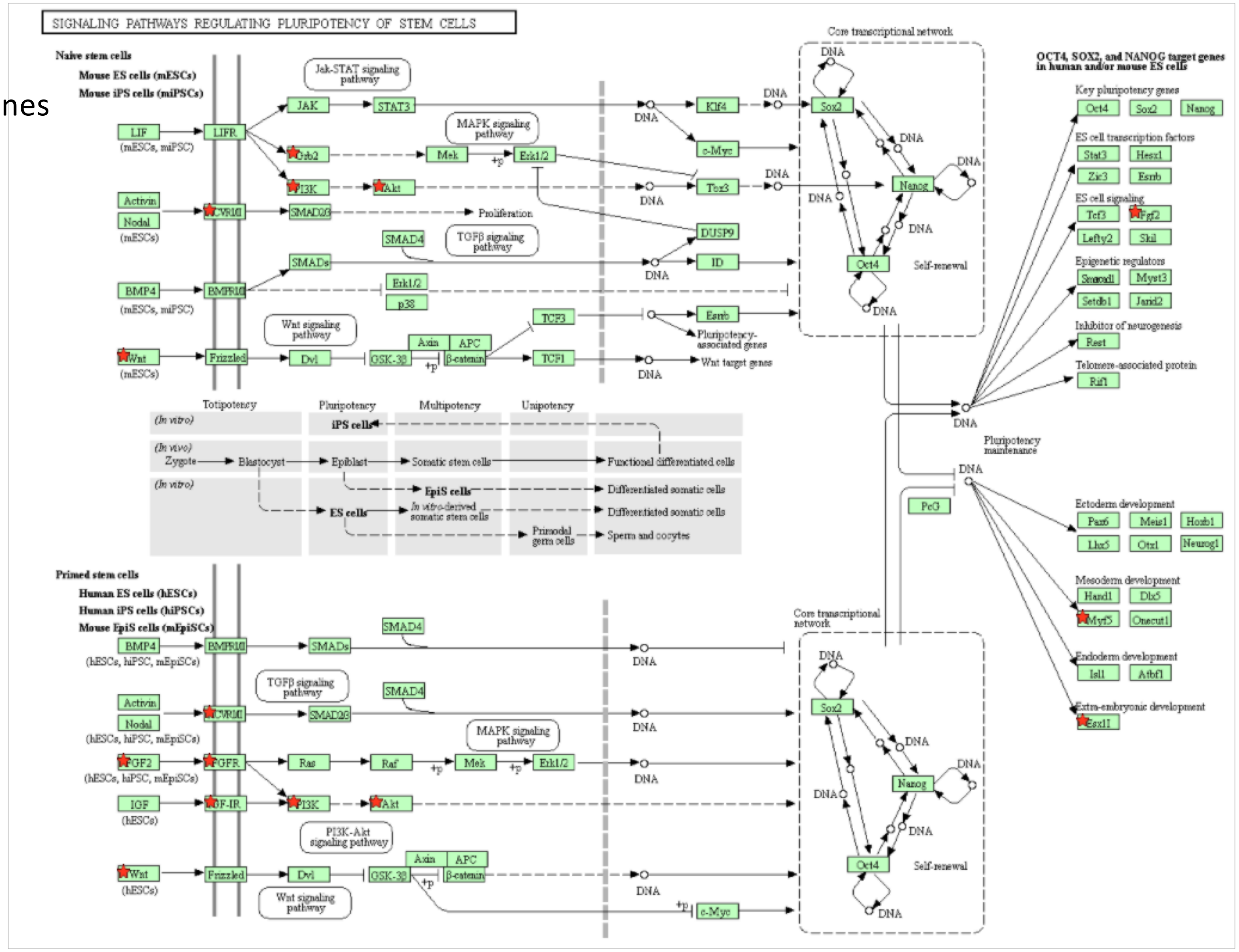

eSF$_{stage\ IV}$

E_2 induced differentially methylated genes

MAPK signaling pathway

★ Genes differentially methylated

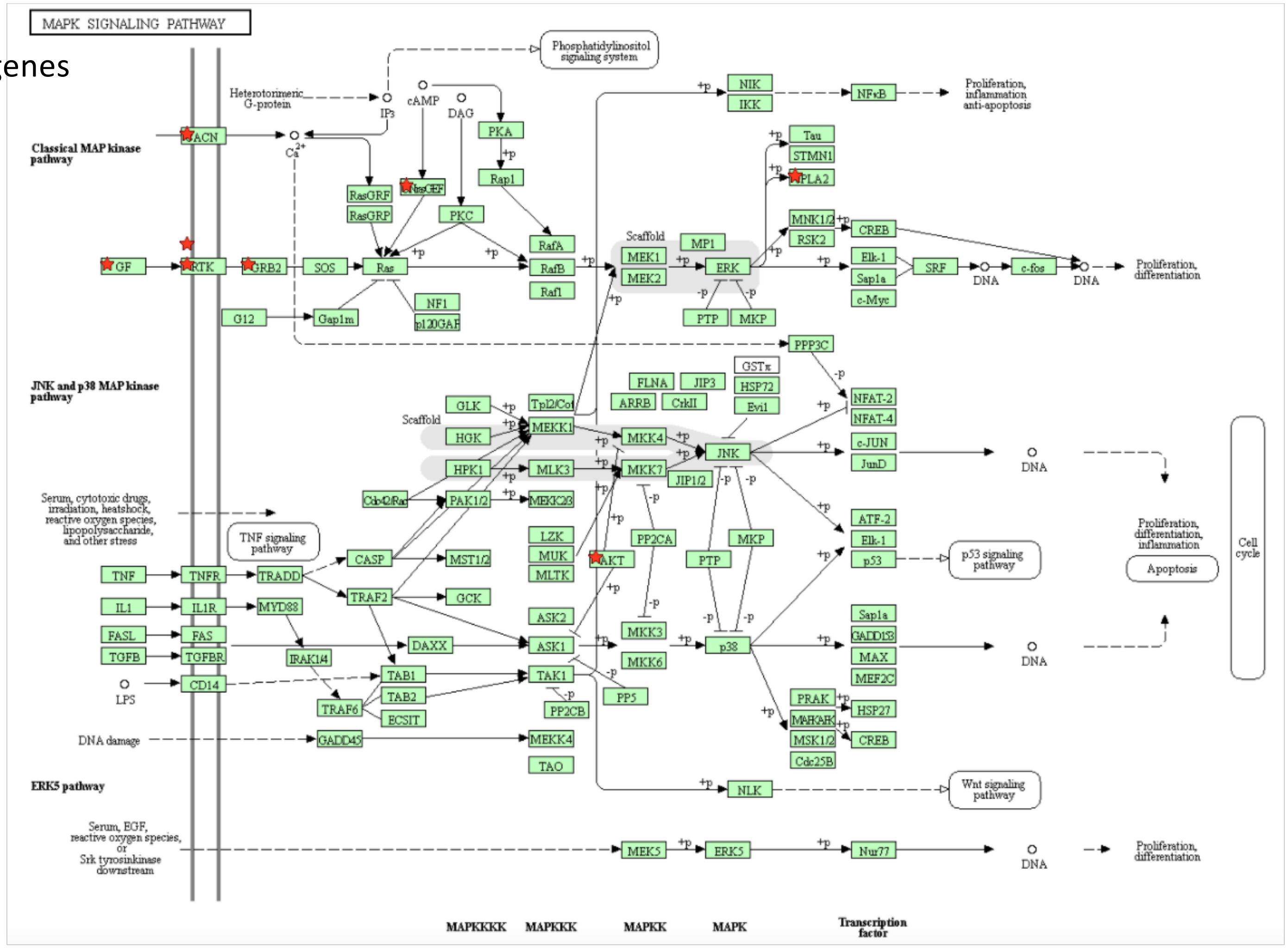

eSF$_{stage\ IV}$

E$_2$ induced differentially methylated genes

Estrogen signaling pathway

★ Genes differentially methylated

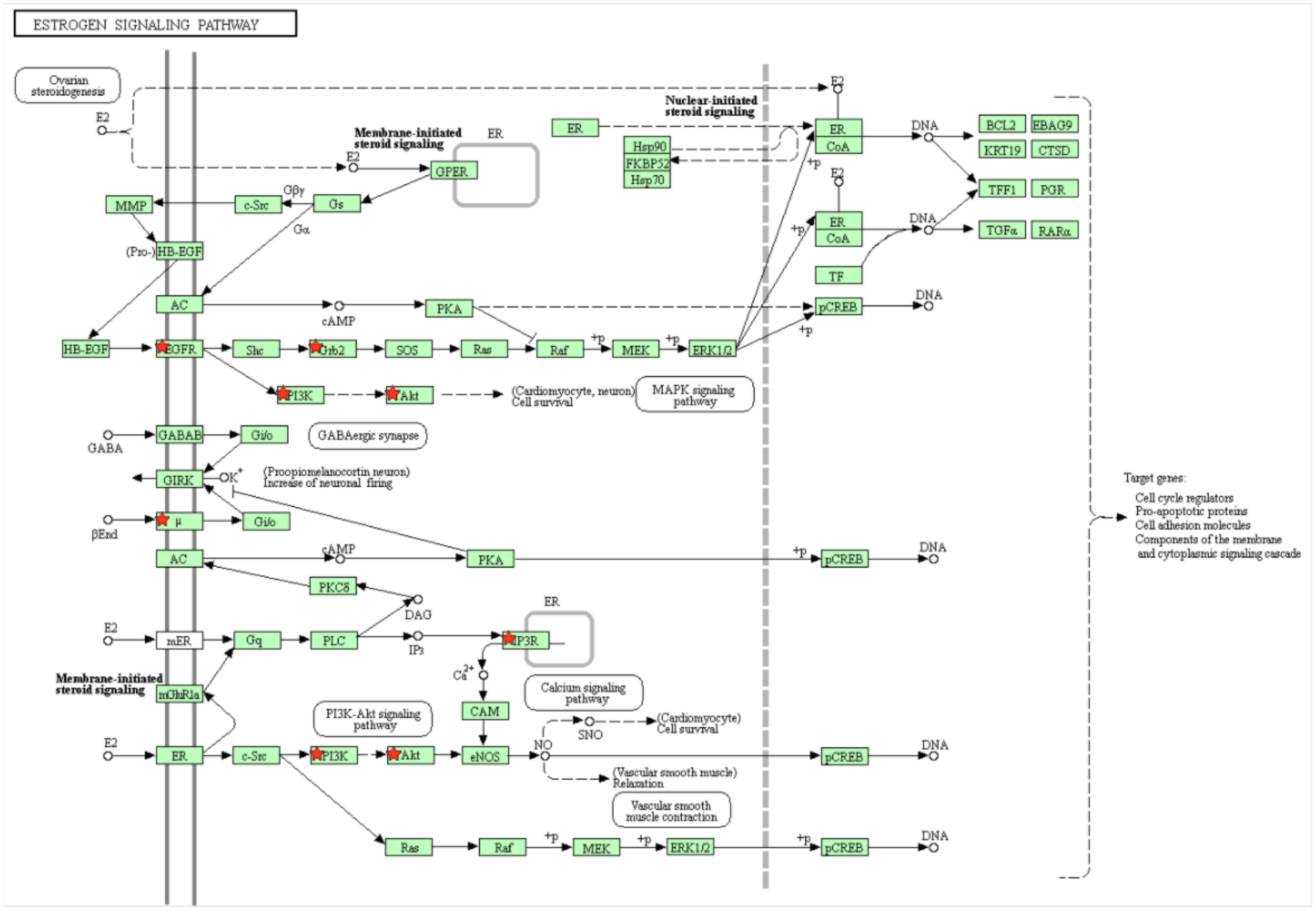

eSF$_{stage\ IV}$

E_2 induced differentially methylated genes

ERBB signaling pathway

★ Genes differentially methylated

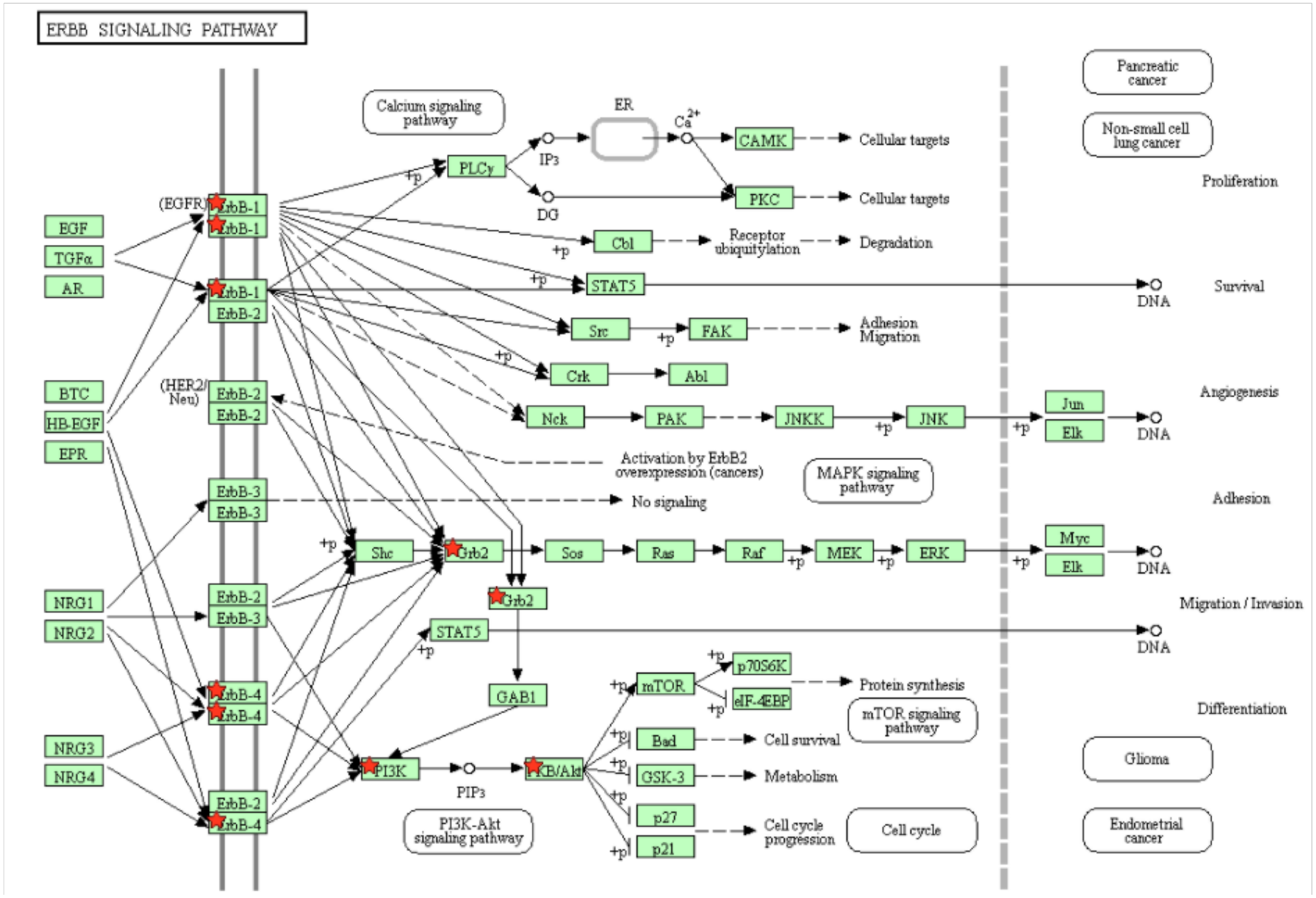

eSF$_{stage\ IV}$

E$_2$ induced differentially methylated genes

RAS signaling pathway

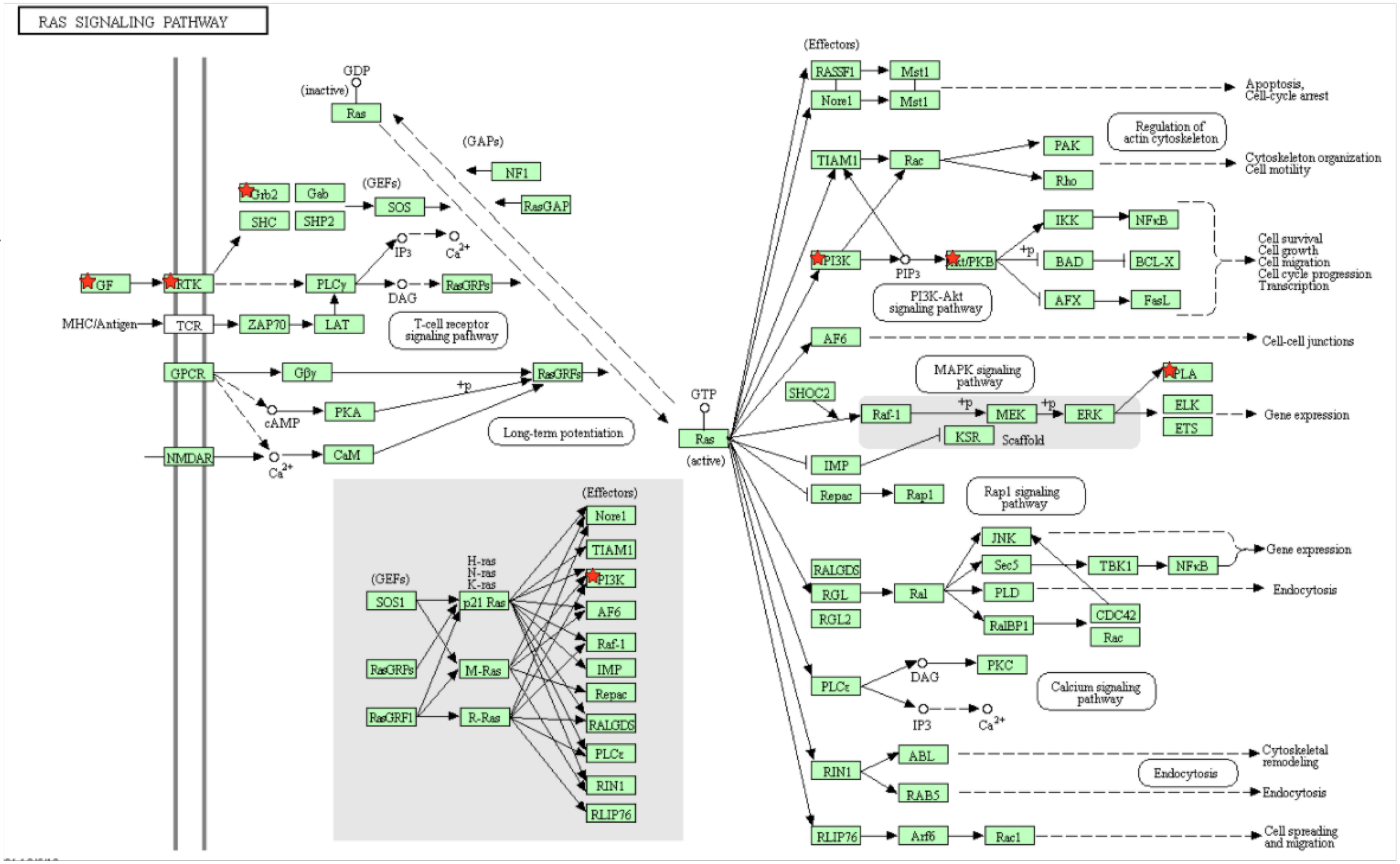

★ Genes differentially methylated

eSF$_{stage\ IV}$

E$_2$ induced differentially methylated genes

FOXO signaling pathway

★ Genes differentially methylated

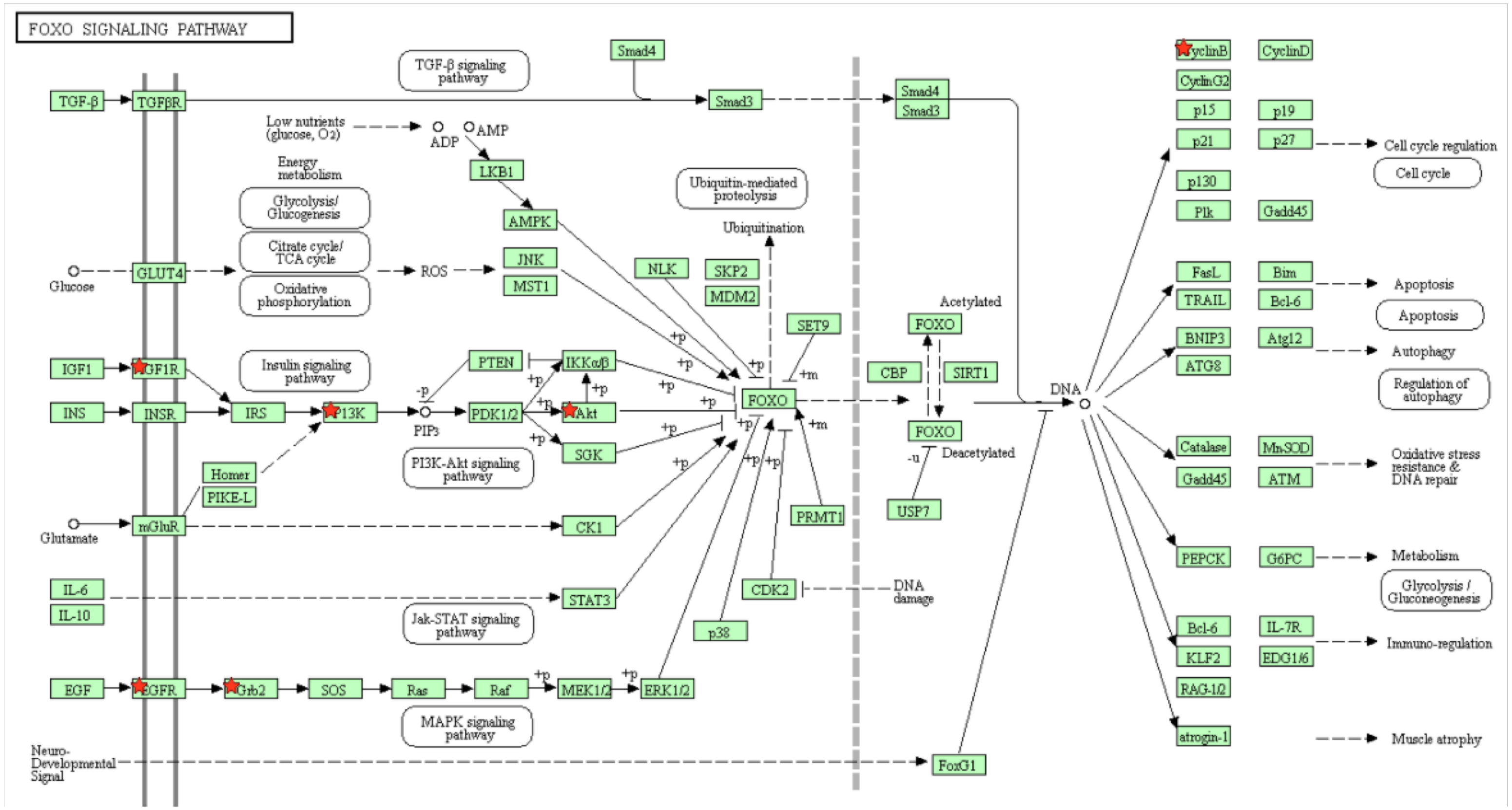

eSF$_{stage\ IV}$

E$_2$ induced differentially methylated genes

RAP1 signaling pathway

★ Genes differentially methylated

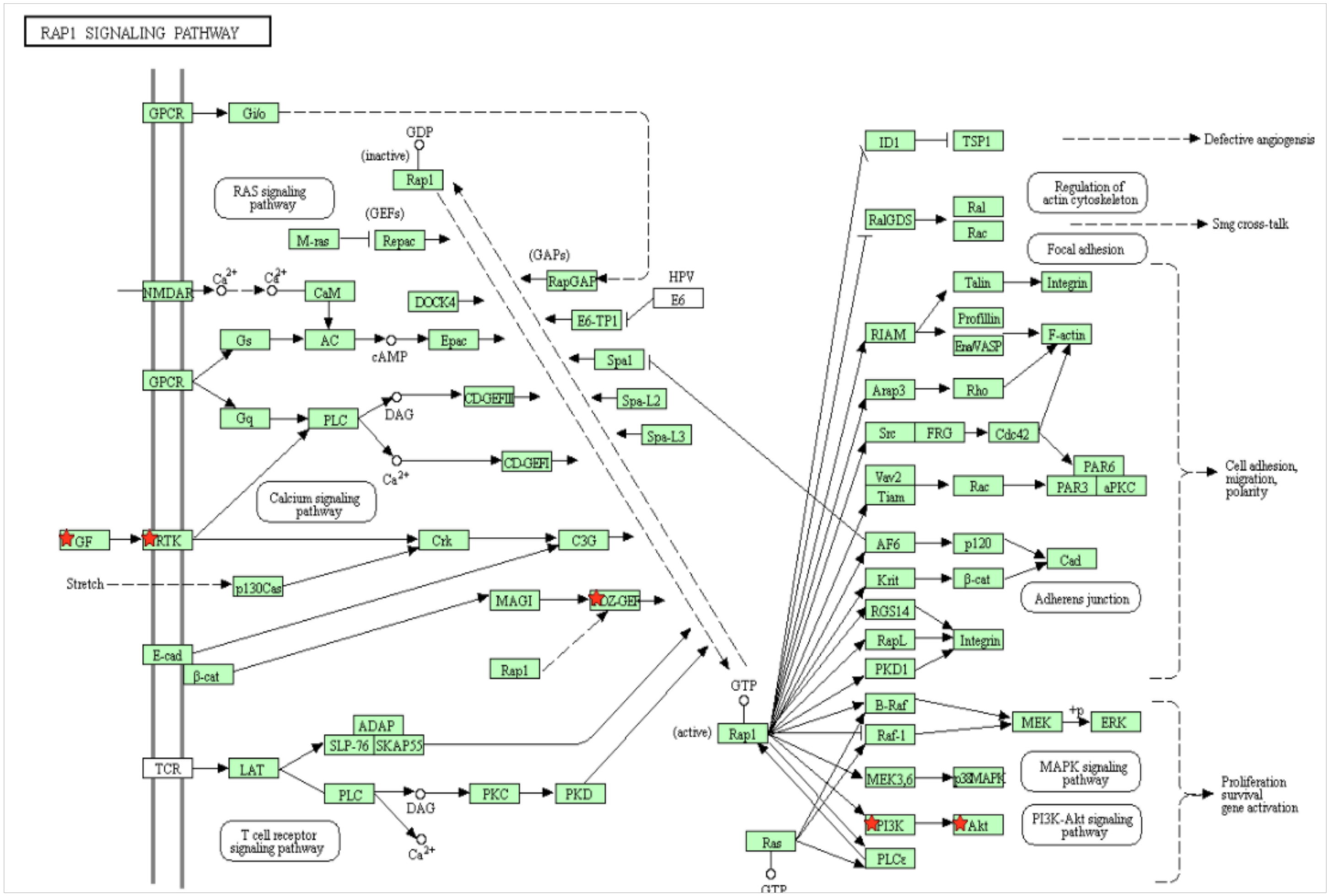

eSF$_{stage\ IV}$
E_2 induced differentially methylated genes
RNA degradation

★ Genes differentially methylated

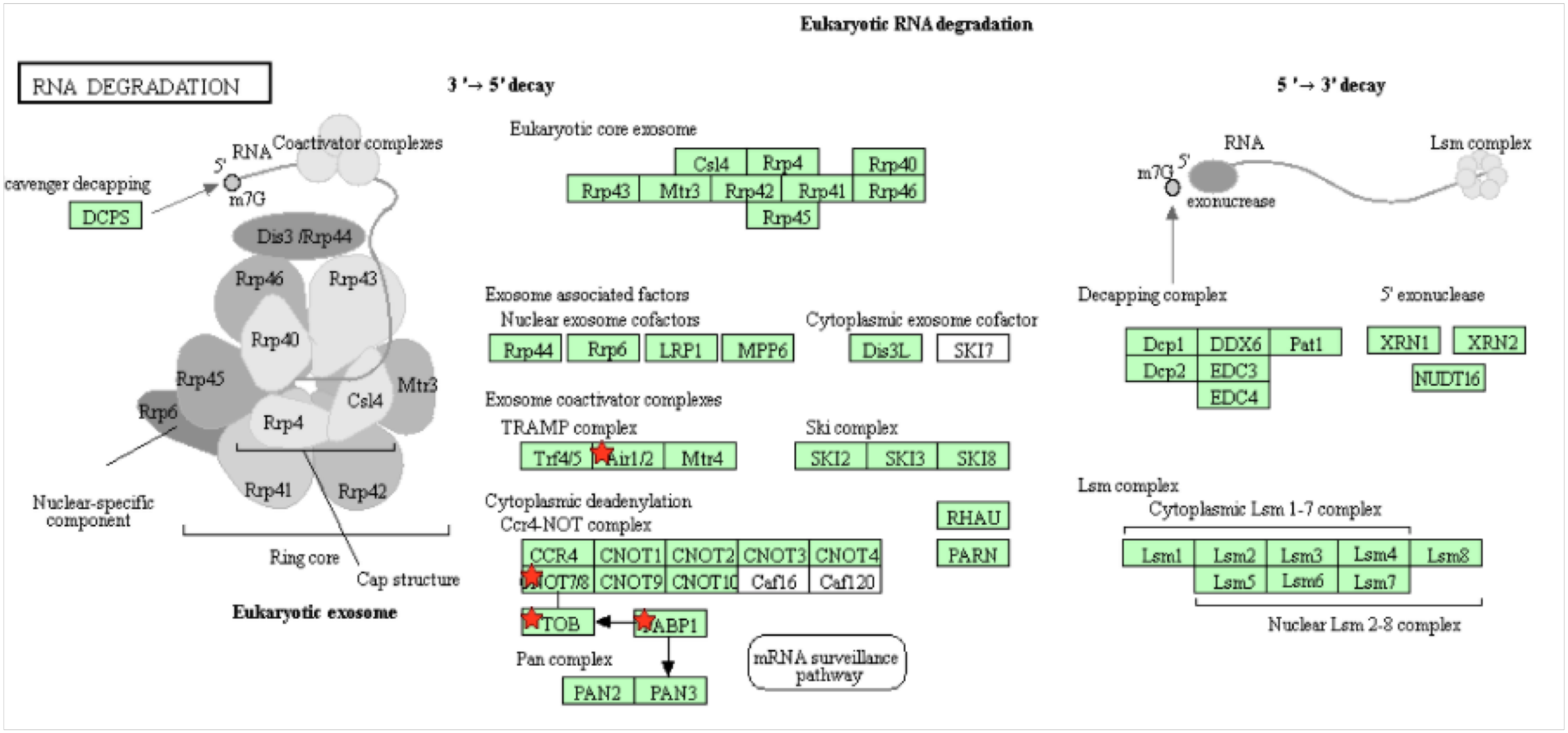

eSF$_{stage\ IV}$

E$_2$ induced differentially methylated genes

Axion guidance

★ Genes differentially methylated

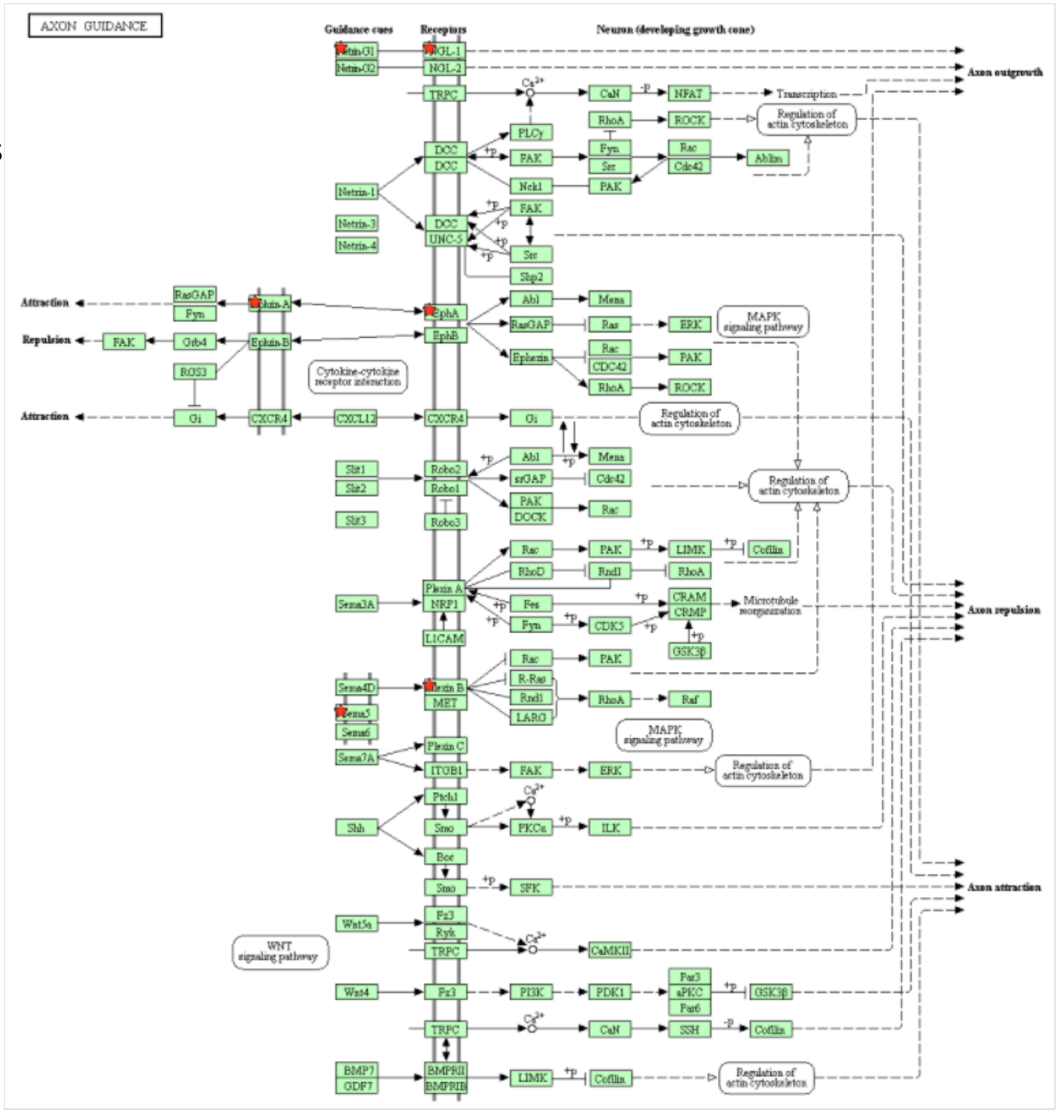

eSF$_{\text{stage IV}}$

E$_2$ induced differentially methylated genes

WNT signaling pathway

★ Genes differentially methylated

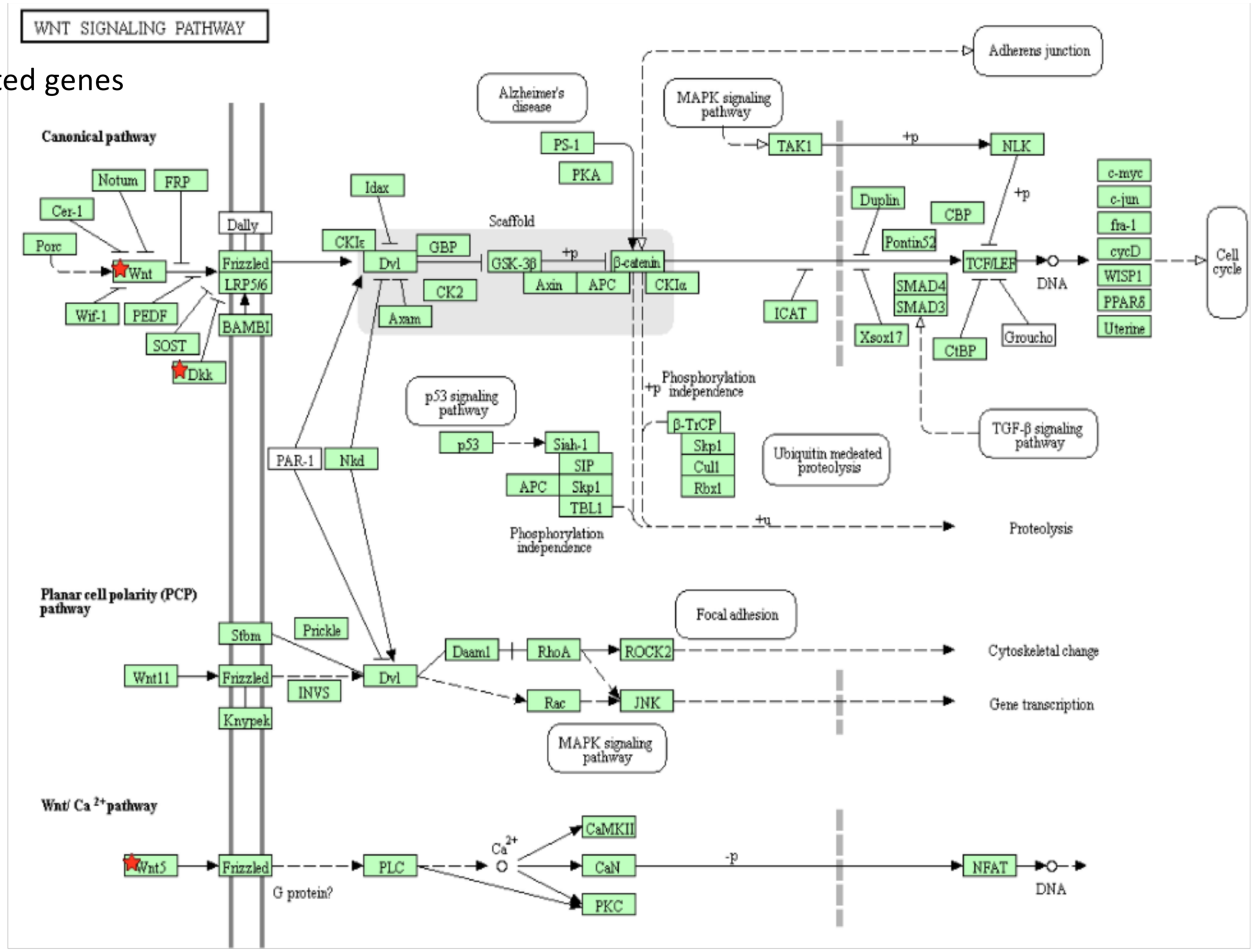

eSF$_{\textbf{stage IV}}$

E$_2$ induced differentially methylated genes

PI3K-AKT signaling pathway

★ Genes differentially methylated

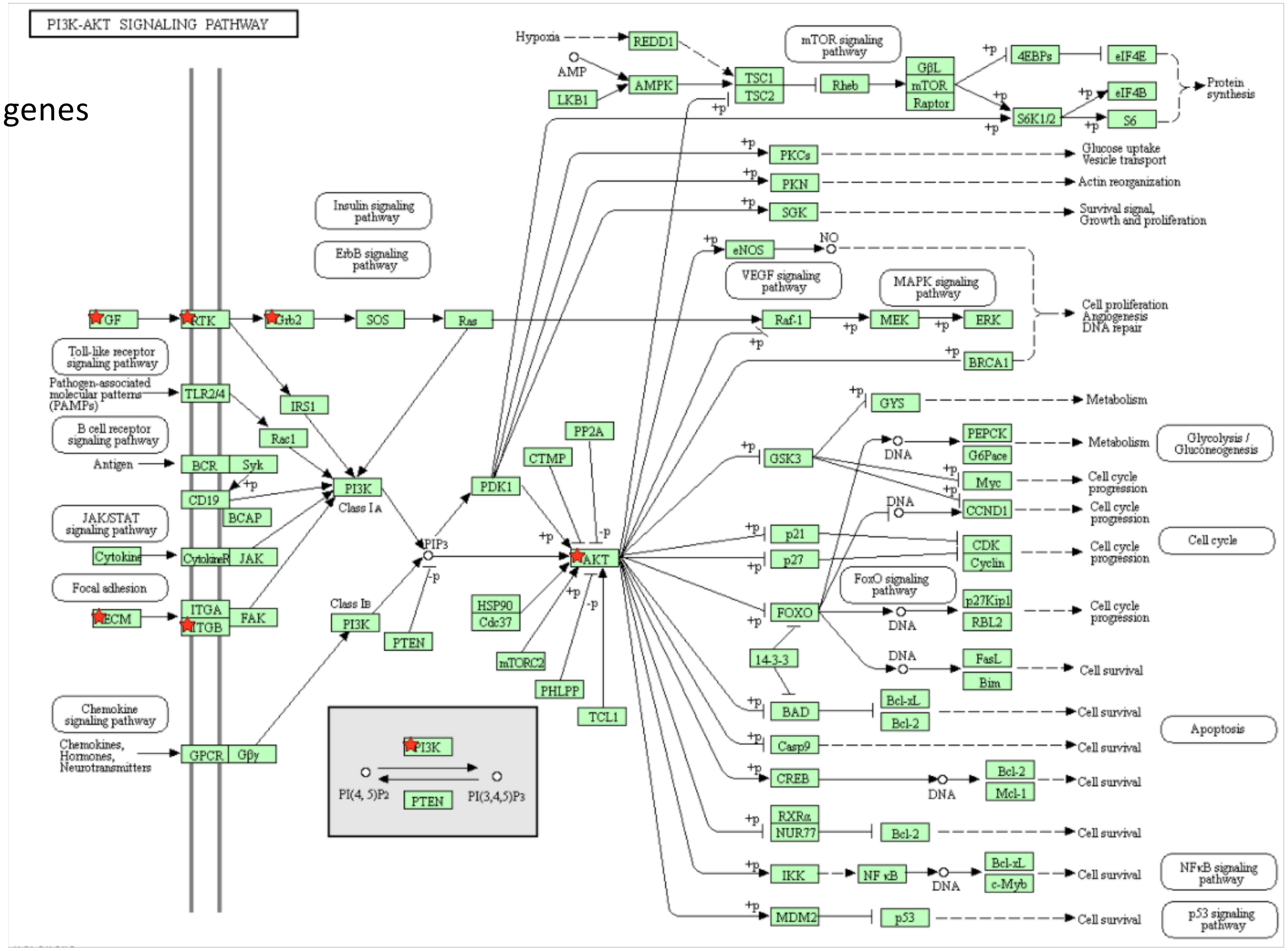

eSF$_{stage\ IV}$
E_2 induced differentially methylated genes
GnRH signaling pathway

★ Genes differentially methylated

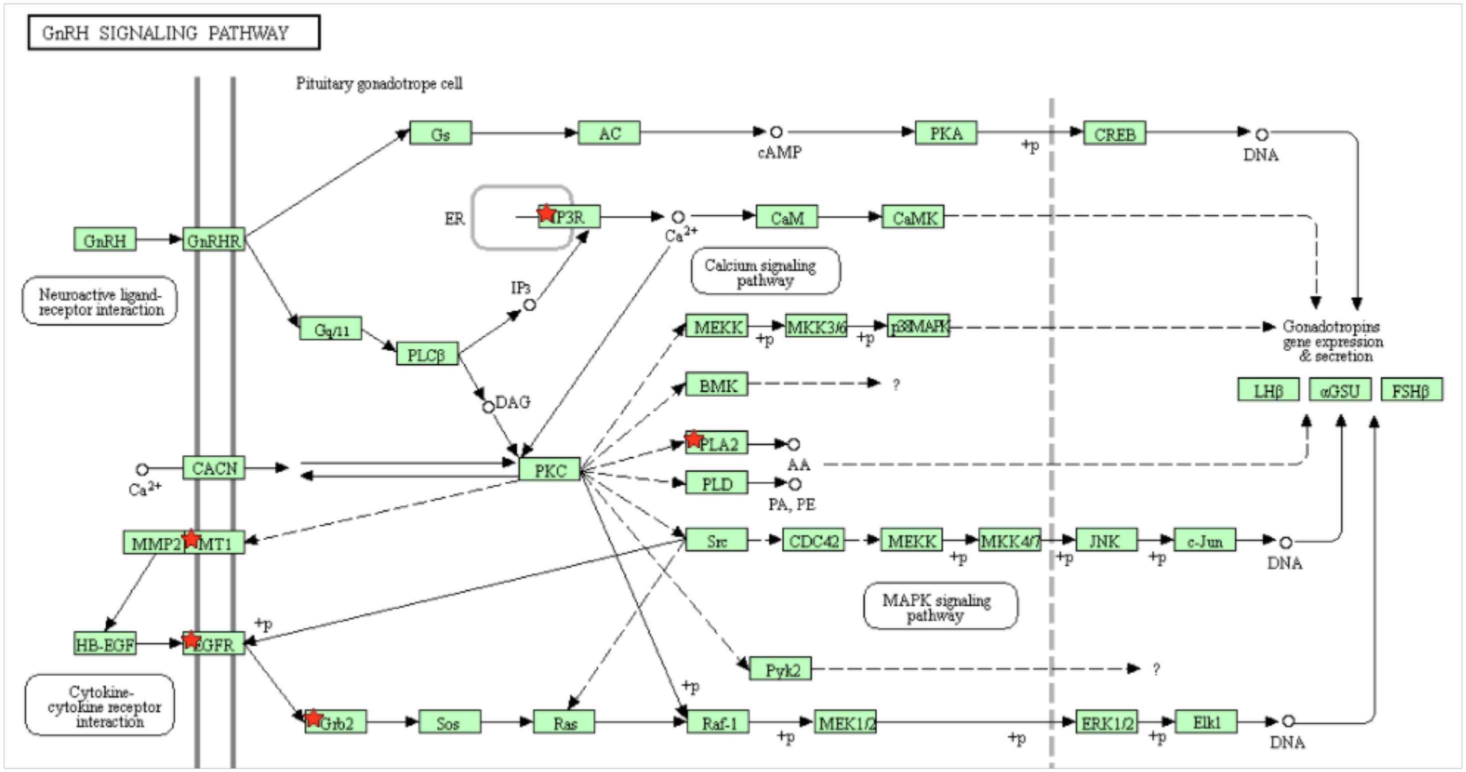

eSF$_{stage\ IV}$

E$_2$ induced differentially methylated genes

Cell adhesion

★ Genes differentially methylated

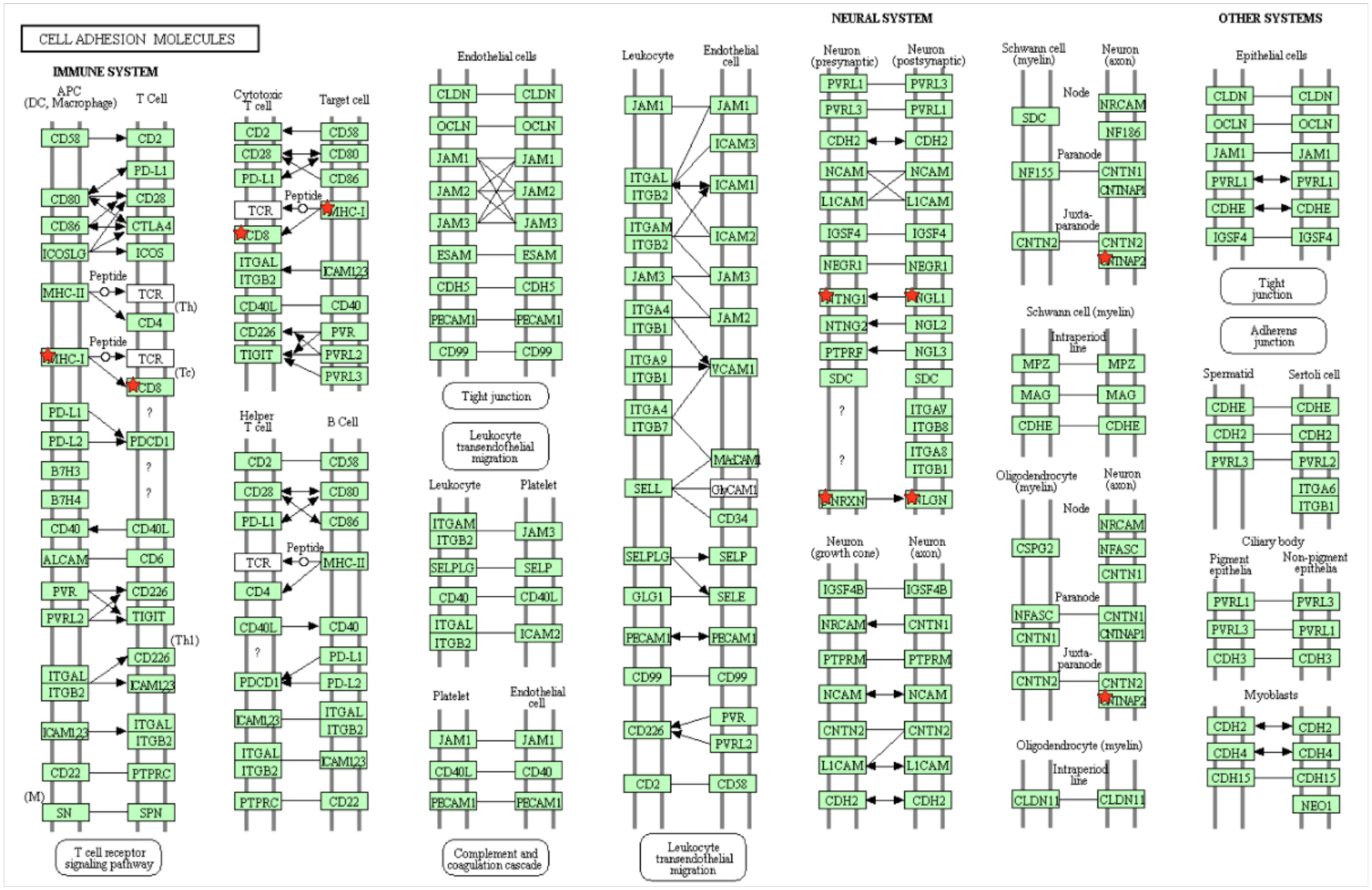

$eSF_{stage\ IV}$

E_2 induced differentially methylated genes

Ovarian Steroidogenesis

★ Genes differentially methylated

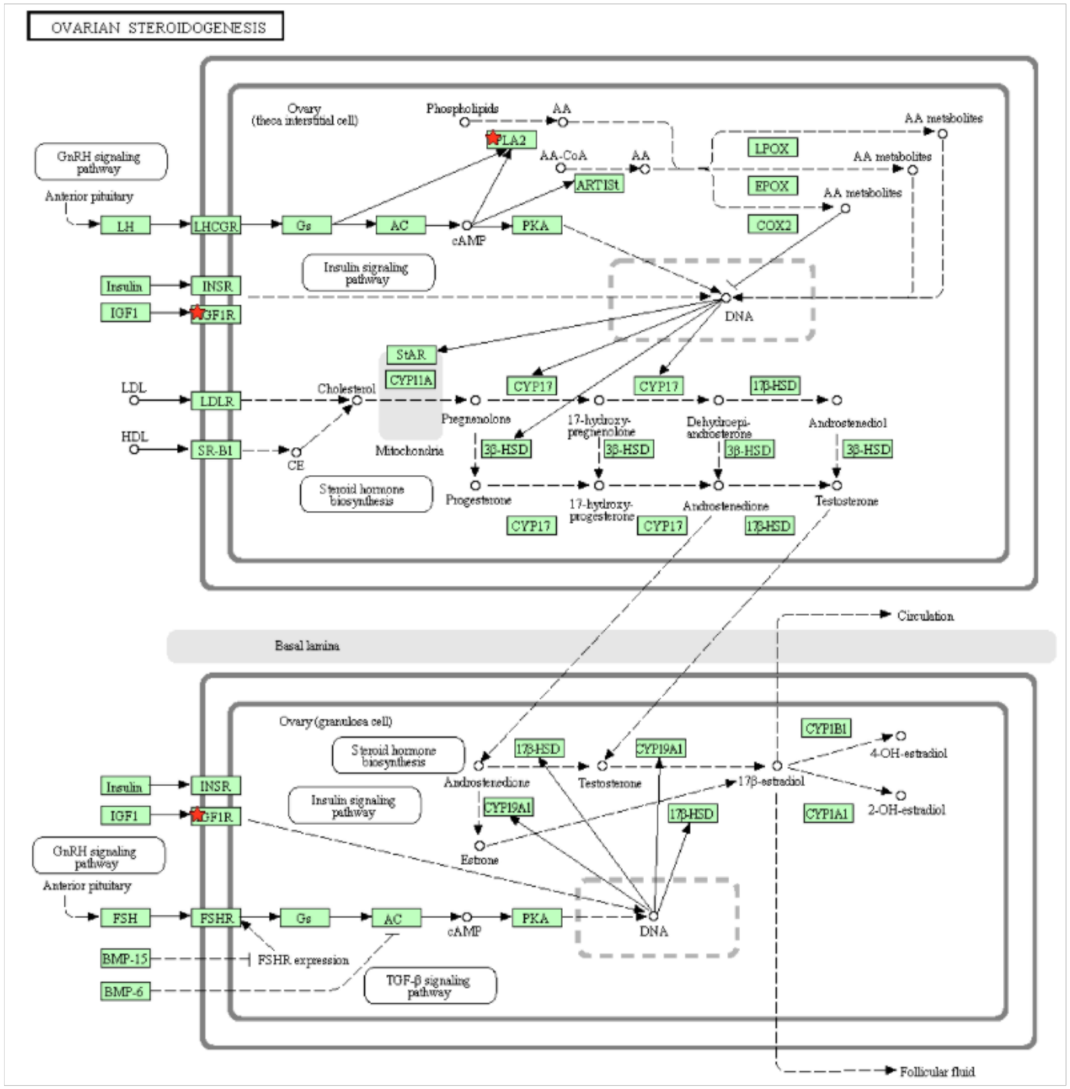

eSF$_{stage\ IV}$
E_2 induced differentially methylated genes
VEGF signaling pathway

★ Genes differentially methylated

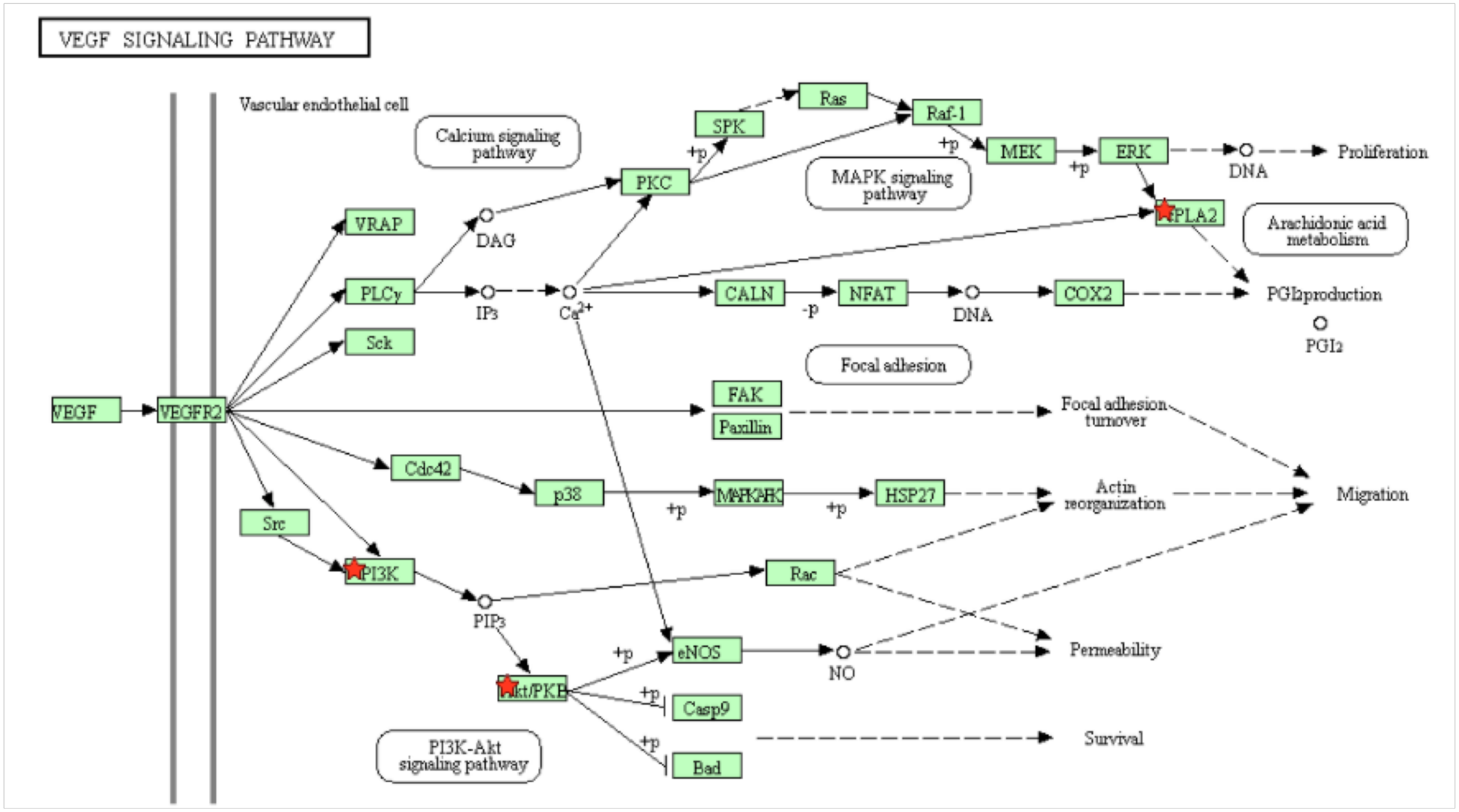

Supplement: S1 Data — (PDF) [file pgen.1008601.s022.pdf]
